# Peptide Marriages: Modular Assembly of Multi‐Agonist Therapeutics

**DOI:** 10.1002/chem.71280

**Published:** 2026-06-18

**Authors:** Kristina A. Kostadinova, Jan L. Venne, Sona Krajcovicova, James Dodgson, Wouter F. J. Hogendorf, Thomas E. Nielsen, David Hymel, Hannah Bolt, Andie Collinson, Jason Day, Jefferson Revell, David R. Spring

**Affiliations:** ^1^ Yusuf Hamied Department of Chemistry University of Cambridge Cambridge UK; ^2^ Department of Organic Chemistry Faculty of Science Palacky University Olomouc Olomouc Czech Republic; ^3^ Biologics Engineering, Oncology R&D AstraZeneca Cambridge UK; ^4^ Therapeutics Discovery Novo Nordisk A/S Måløv Denmark; ^5^ Novo Nordisk US R&D Lexington Massachusetts USA; ^6^ Discovery Sciences, BioPharmaceuticals R&D AstraZeneca Cambridge UK; ^7^ Department of Earth Sciences University of Cambridge Cambridge UK

**Keywords:** amycretin, dual peptide agonists, peptide therapeutics, semaglutide, solid‐phase synthesis

## Abstract

Multi‐receptor peptide agonists represent an effective strategy for obesity treatment, extending the success of incretin‐based therapies through simultaneous engagement of complementary targets. Their development, however, is synthetically demanding, as each receptor combination typically requires *de novo* preparation of large fusion peptides. We report a modular polyethylene glycol (PEG)‐based scaffold that enables orthogonal attachment of up to three functional components, including therapeutic peptides, half‐life–extending units, and other labels, via sequential strain‐promoted azide–alkyne cycloaddition (SPAAC) and copper‐catalysed azide–alkyne cycloaddition (CuAAC). The scaffold is assembled on solid phase without intermediate purification, providing a readily accessible and versatile linker. Using glucagon‐like peptide‐1 (GLP‐1) and amylin receptor agonists as a proof‐of‐concept, dual‐agonist constructs with tuneable valency and functionality were rapidly generated. Lead conjugates displayed balanced, low‐picomolar potency at both receptors in cyclic adenosine monophosphate (cAMP) assays and showed selective receptor‐mediated internalisation in GLP‐1 receptor‐expressing cells. This orthogonal click‐based platform enables rapid and modular multi‐agonist assembly, facilitating systematic exploration of receptor combinations, valency, and payload effects. Beyond incretin biology, it offers a general route to multifunctional peptide therapeutics and diagnostics.

## Graphical abstract

A modular polyethylene glycol (PEG) scaffold enables rapid assembly of dual peptide agonists using strain‐promoted azide–alkyne cycloaddition (SPAAC) and copper‐catalysed azide–alkyne cycloaddition (CuAAC). Glucagon‐like peptide‐1 (GLP‐1) and amylin agonists were combined with controlled valency, yielding low‐picomolar activity and receptor‐selective internalisation, establishing a versatile platform for multifunctional peptide therapeutics.

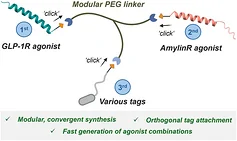

## 1 Introduction

Compounds targeting class B G protein‐coupled receptors (GPCRs), including glucagon‐like peptide‐1 (GLP‐1) and gastric inhibitory peptide (GIP) receptor agonists, have emerged as powerful medicines for treating metabolic disorders [1, 2, 3, 4]. Initially designed to improve glycaemic control in Type 2 Diabetes Mellitus, incretin‐based drugs have also shown substantial benefits in obesity management [5, 6, 7, 8]. Nevertheless, treatment outcomes differ between individuals, with some patients requiring further optimisation to achieve desired results due to biological and tolerability limitations [9]. To address these limitations, next‐generation therapeutics targeting additional receptors, such as the glucagon or amylin receptors, are under active development [10, 11, 12, 13, 14, 15] (Figure 1A; Previous work).

**FIGURE 1:**
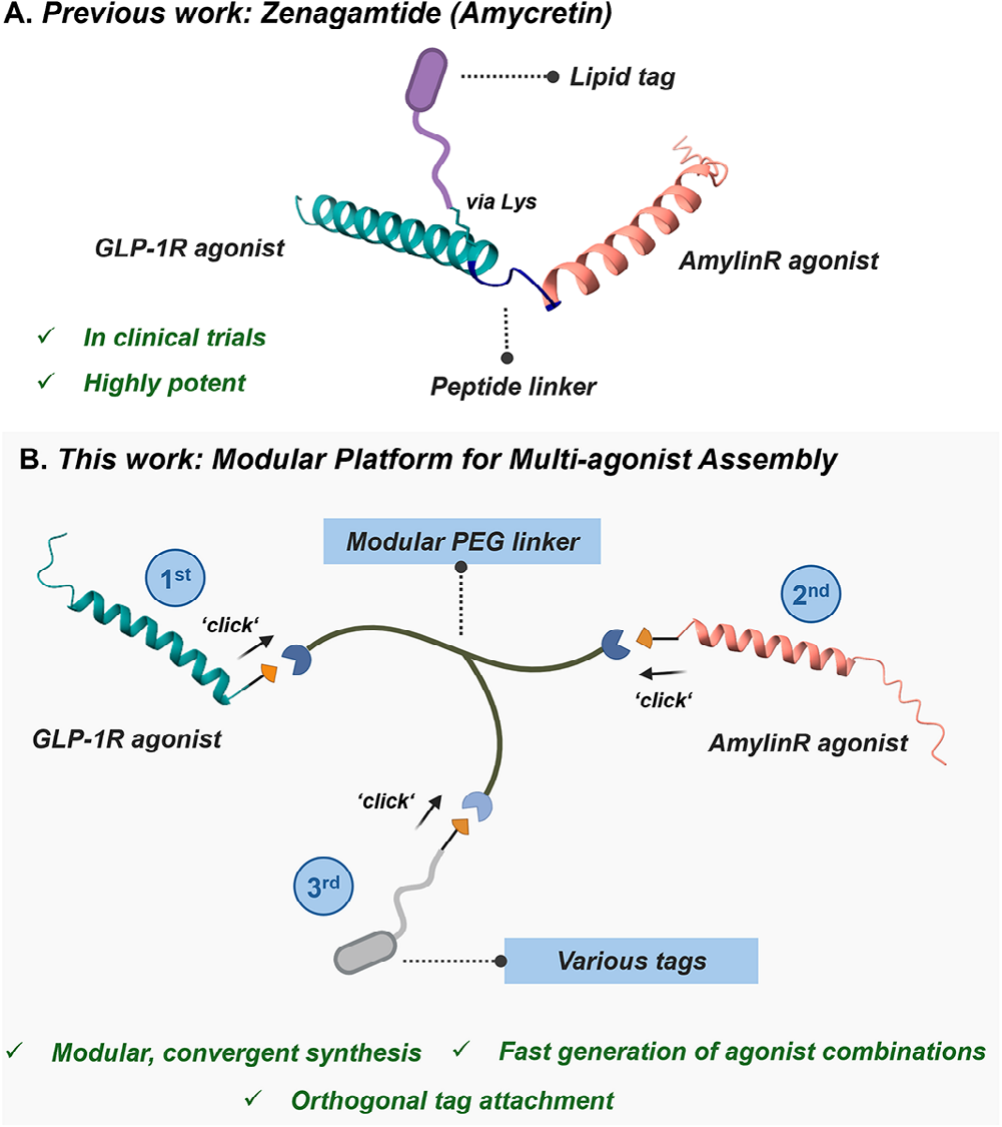
Comparison of previous work that has been done on zenagamtide (amycretin) [16] versus this work. Peptide secondary structures are only illustrative.^1^

The principle of multi‐agonism, whereby several metabolic GPCRs are co‐activated to achieve synergistic metabolic effects, has become a well‐established strategy in the development of next‐generation incretin therapeutics [17, 18, 19, 20, 21, 22]. Unimolecular dual and triple agonists such as tirzepatide (GLP‐1R/GIPR) [4], retatrutide (GLP‐1R/GIPR/Glucagon) [23], and zenagamtide (previously known as amycretin; GLP‐1R/Amylin) [24] have achieved impressive clinical and preclinical outcomes, offering superior metabolic control and weight reduction compared to single‐receptor agents. More recently, tetra‐agonists targeting GLP‐1, GIP, glucagon, and amylin receptors have been reported, highlighting the therapeutic potential of broad multi‐receptor engagement [25]. Agents like zenagamtide [24] (Figure 1A), which acts as a dual agonist, have already shown promising weight reductions [16, 24, 26].

Despite these advances, the optimal combination and stoichiometric balance of receptor targets for maximal efficacy and tolerability remain unclear [27]. Designing such multi‐agonists presents substantial synthetic and pharmacological challenges [28, 29]. The potency of individual peptide agonists does not reliably predict their performance when incorporated into larger unimolecular constructs. For instance, a zenagamtide (amycretin) analogue in which the amylin receptor agonist is replaced by the pramlintide sequence shows much lower potency at the amylin receptor (AmyR3; 606 pM) compared to zenagamtide itself (11.5 pM), despite similar individual potencies of pramlintide (7.8 pM) and the original AmyR3 agonist peptide (5 pM), when tested as individual peptides outside the co‐agonist context [30]. This context‐dependent activity underscores the need for systematic exploration of diverse peptide combinations, which in turn demands synthetic approaches that are both rapid and modular. However, fusion‐type agonists are large (∼70 amino acids for zenagamtide [16, 24]) and challenging to access by solid‐phase peptide synthesis (SPPS), which must be repeated for each new variant [31]. Further complexity arises from auxiliary structural features such as lipid side chains or half‐life–extending elements that strongly influence pharmacokinetic and pharmacodynamic profiles, often in unpredictable ways [12, 32, 33]. Consequently, the design–make–test cycle for multi‐agonist peptides remains slow and resource‐intensive, limiting exploration of receptor balance, valency, and molecular architecture.

To address these challenges, we developed a modular polyethylene glycol (PEG)‐based scaffold capable of orthogonal conjugation of up to three therapeutic peptides, as well as half‐life–extending or imaging payloads (Figure 1B; This work). Using a combination of well‐established strain‐promoted azide–alkyne cycloaddition (SPAAC) and copper‐catalysed azide–alkyne cycloaddition (CuAAC) chemistries, this platform enables rapid assembly of multi‐agonist constructs from pre‐synthesised peptide modules. Integrating orthogonal chemistries within a modular platform overcomes key limitations of unimolecular peptide fusion strategies, enabling rapid construction and optimisation of multi‐agonist peptides with controllable receptor balance and payload composition for fast pre‐clinical de‐risking of potential next‐generation incretin therapeutics.

## 2 Results and Discussion

### 2.1 Design and Synthesis of the Modular Linker Scaffold **4**

The synthesis of the modular PEG‐based linker scaffold **4** commenced with commercially available 2‐chlorotrityl chloride (2‐CTC) resin preloaded with H‐Gly‐OH, followed by amide coupling with a commercially available alkyne‐functionalised lysine amino acid (Scheme 1). On resin solid‐phase synthesis was selected as a practical alternative to conventional solution‐phase methods, as it enables efficient incorporation of PEG chains with quantitative conversions and high crude purity upon cleavage, thereby eliminating the need for intermediate purification.

**SCHEME 1:**
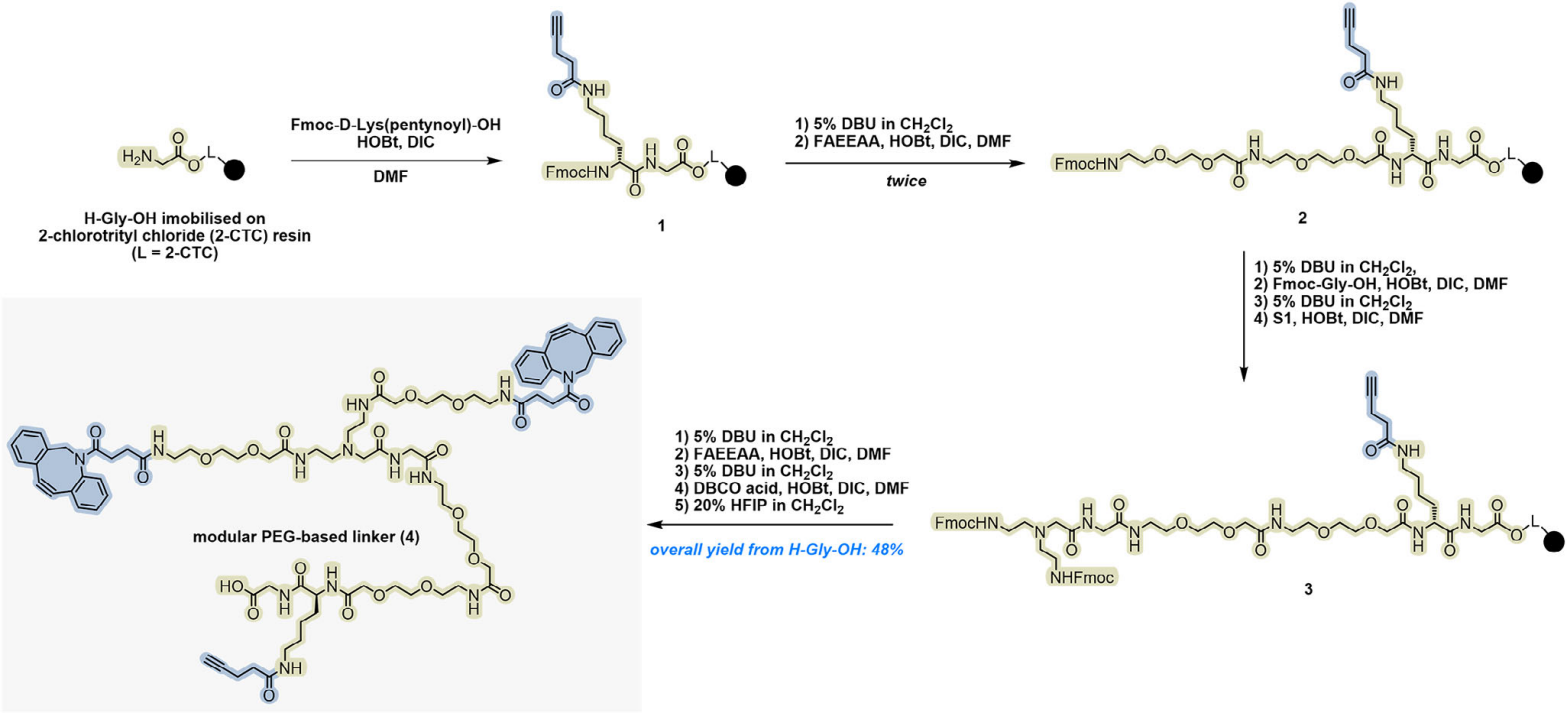
Solid‐phase synthesis of the modular three‐component PEG linker. As the lysine residue serves primarily as a spacer bearing the alkyne functionality, its stereochemistry is not expected to affect the reactivity of the linker DBCO = dibenzocyclooctyne, DBU = 1,8‐diazabicyclo[5.4.0]undec‐7‐ene, DIC = *N, N*‐diisopropylcarbodiimide, DMF = dimethylformamide, FAEEAA = {2‐[2‐(Fmoc‐amino)ethoxy]ethoxy}acetic acid, HFIP = hexafluoroisopropanol, HOBt = 1‐hydroxybenzotriazol, L = 2‐chlorotrityl chloride resin, PEG = polyethyleneglycol, **S1** = key branching amine compound (see Supporting Information Section 1.4.4. for full synthesis).

By maintaining intermediates on resin and removing excess reagents through simple filtration, solid‐phase synthesis streamlines the workflow and accelerates production. The approach minimises hands‐on time and purification steps while requiring only standard laboratory equipment, making it readily accessible to most synthetic laboratories. The 2‐chlorotrityl chloride (2‐CTC) resin was chosen to enable mild HFIP cleavage, avoiding degradation of the acid‐sensitive dibenzocyclooctyne (DBCO) core under strong acid [34]. Notably, for alternative linker architectures or payloads bearing acid‐labile groups, orthogonal protection strategies for DBCO moiety [35] could be implemented to permit the use of other resin types such as Rink amide resin, that requires harsher acidic conditions, thus expanding the applicability of the synthetic route. Deprotection of the Fmoc group from intermediate **1** was carried out using 1,8‐diazabicyclo[5.4.0]undec‐7‐ene (DBU) in CH_2_Cl_2_, followed by iterative cycles of amide coupling and Fmoc removal using commercially available {2‐[2‐(Fmoc‐amino)ethoxy]ethoxy}acetic acid (FAEEAA) to afford intermediate **2**.

To introduce a branched architecture and ensure equal spacing between the two SPAAC handles, Fmoc‐Gly‐OH and our previously reported [36] key branching amine **S1** were coupled to the resin, followed by an additional FAEEAA coupling/deprotection sequence. This modular design was intended to yield symmetrical DBCO termini, allowing predictable reaction stoichiometry and efficient formation of orthogonal bis‐conjugates under controlled SPAAC conditions. The synthesis was completed by attaching DBCO acid to the resin to yield final linker scaffold **4**. The complete assembly could be achieved within three days without intermediate purification, affording the linker in >95% crude purity, as estimated by UV‐HPLC peak‐area integration after final cleavage from the resin (Figure S2, Supporting Information Section 1.4.5).

The isolated linker exhibited excellent storage stability, remaining unchanged as a solid when kept at −4°C for at least six months. Solution stability was evaluated in phosphate‐buffered saline (PBS) at pH 7.4 with approximately 80% of the linker remaining after 24 h at physiological temperature (37°C) and more than 70% integrity retained after six days at ambient temperature (Figure S20, Supporting Information Section 3). Such stability comfortably covers the timescales throughout the pharmacologically relevant window of zenagamtide (amycretin), ranging from the rapid clearance in mice [24] to the more prolonged half‐life observed in human clinical models (up to 100 h) [16]. The minor degradation observed is therefore unlikely to compromise biological performance and is most plausibly attributed to slow hydrolysis of the DBCO amide functionalities (see below). Overall, this streamlined solid‐phase approach not only simplifies linker synthesis but also provides a robust, reproducible platform for rapid preparation of multifunctional scaffolds with high chemical homogeneity.

### 2.2 Design and Assembly of the Multipurpose Dual Agonists Based on Zenagamtide (Amycretin)

With the PEG‐based linker **4** in hand, the feasibility of the modular assembly strategy was evaluated. As a proof‐of‐concept, zenagamtide was selected, a dual agonist with well‐established biological activity [16, 24, 30]. Its constituent peptide components—agonists of the GLP‐1 and amylin receptors—were synthesized via solid‐phase peptide synthesis (SPPS), each bearing a modified lysine with an azido group compatible with the linker's click handles. The azido‐modified lysine (labelled as ‘K^N3^’) was introduced at the C‐ and N‐termini, respectively, allowing for orthogonal conjugation to the central linker (see Supporting Information Section 1.3 for full structures). The dual agonist was assembled in a stepwise fashion via SPAAC, beginning with the conjugation of the GLP‐1 agonist **5** (Figure 2A). Pleasingly, only a single SPAAC reaction was observed under these conditions, using linker **4** in 2.5‐fold molar excess relative to the agonist **5**. The selectivity of the first SPAAC reaction was confirmed by LC–MS analysis of the crude mixture (Figure S18, Supporting Information Section 2.1), which showed exclusive formation of the mono‐functionalised product, consistent with our previous observations using an analogous bis‐DBCO system [36]. We hypothesise that high selectivity can be achieved using a modest 2.5 equivalents excess of linker, as the steric bulk of the conjugated peptide moieties is expected to suppress a second SPAAC reaction. The central linker scaffold **4** exhibited excellent chemical stability in PBS at ambient temperature for at minimum of 24 h (Figure S19, Supporting Information Section 3). For compound **6,** the remaining DBCO moiety was found to be sensitive to extended exposure to aqueous conditions; however, this behavior was readily managed through appropriate handling and minimising water during the reaction and work‐up. The second SPAAC step (Figure 2A), introducing the amylin receptor agonist **7**, proceeded without observable hydrolysis of the final dual conjugate. To further demonstrate the versatility of the approach and evaluate the impact of additional modifications on agonist performance, a third functionality was introduced via CuAAC (Figure 2A).

**FIGURE 2:**
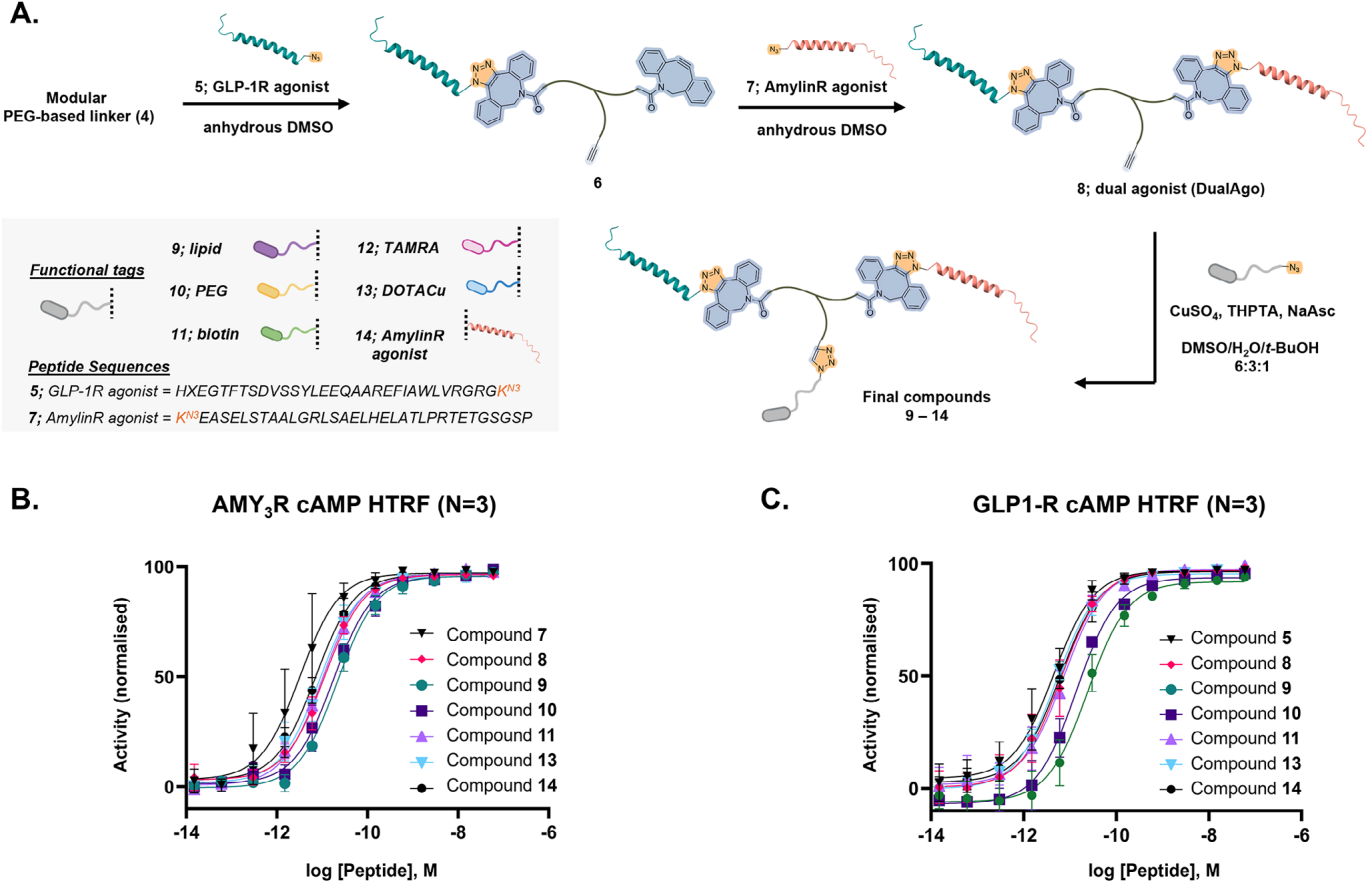
(A). Final assembly of the multipurpose dual agonists **9–14**. See Supporting Information Section 1.3 for full structures and possible regioisomers. Yields upon HPLC purification: **5** = 23% (overall peptide synthesis); **6** = 33%; **7** = 7% (overall peptide synthesis); **8** = 57%; **9** = 48%; **10** = 42%; **11** = 37%; **12** = 22%; **13** = 40%; **14** = 40%. Linker cartoons were created in BioRender (https://BioRender.com/gwhb1a5); (B). HTRF cAMP Gs Dynamic Detection assay comparing AmylinR agonist **7**, Compounds **8–11**, **13–14** in cAMP HunterTM CHO‐K1 CALCRL‐RAMP3 Gs cells. (C). HTRF cAMP Gs Dynamic Detection assay comparing GLP1‐R agonist **5**, Compounds **8–11**, **13–14** in CHO‐GLP1R cells. See Supporting Information Section 3.1 for comparison to davalintide (positive control), and DMSO (negative control). Data are presented as mean ± SD from three independent experiments (*n* = 3), each performed in technical triplicate. Some error bars may not visible due to being smaller than the data point symbols on the graph. Curves were fitted by nonlinear regression using a four‐parameter logistic model. No statistical comparisons were performed. CALCRL = Calcitonin Receptor‐Like; cAMP = Cyclic Adenosine Monosphosphate, DMSO = dimethyl sulfoxide (anhydrous purchased from Thermo‐Fisher Scientific cat. no. 348441000), DOTACu = dodecane tetraacetic acid with chelated copper, Gs = G stimulatory, HTRF = Homogeneous Time Resolved Fluorescence, NaAsc = sodium ascorbate, RAMP3 = Receptor Activity Modifying Protein 3, THPTA = tris(3‐hydroxypropyltriazolylmethyl)amine, *t*‐BuOH = *tert*‐butanol, TAMRA = 5‐carboxytetramethylrhodamine, X = 2‐Aminoisobutyric acid (Aib).

This orthogonality arises from the fact that the terminal alkyne remains inert unless activated by copper catalysis. In addition to the lipid‐based half‐life extension group derived from zenagamtide and semaglutide, a variety of functional tags were incorporated, each selected to highlight different applications: biotin for affinity‐ based enrichment and further modification; tetramethylrhodamine (TAMRA) as a fluorescent label; and dodecane tetraacetic acid (DOTA), a macrocyclic chelator commonly used in diagnostic and therapeutic radiopharmaceuticals [37]. All appended moieties (resulting in final compounds **9–13**) were either commercially available or readily prepared on resin. Moreover, to demonstrate control over agonist stoichiometry and the potential to enhance activation of one of the receptors, a conjugate **14** with a 2:1 amylin‐to‐GLP‐1 ratio was synthesized by sequential attachment of a second amylin receptor agonist via CuAAC. Given that residual copper can pose challenges in biological applications, copper levels after CuAAC were quantified by inductively coupled plasma mass spectrometry (ICP‐MS) analysis, which enables sensitive detection of trace metal contaminants. Pleasingly, the copper content in the final conjugates **9–12** after the standard preparative HPLC purification (Table S4) was well below both the daily oral and parenteral exposure to copper^2^, assuming similar doses to zenagamtide [16]. Compounds **13** and **14** contained modestly higher copper levels, while remaining well below the daily oral exposure threshold. This is readily rationalized by the presence of a DOTA chelator in compound **13** and by the increased coordination capacity of the larger peptide scaffold in compound **14**. Notably, the observed copper levels did not interfere with in vitro biological evaluation, and if required for specific downstream applications, further reduction could be straightforwardly achieved using established chelation strategies such as ethylenediaminetetraacetic acid (EDTA) [38].

### 2.3 Biological Validation of the Zenagamtide‐Based Final Constructs

Final constructs were evaluated in cellular cyclic adenosine monosphosphate (cAMP) accumulation assays to assess agonist activity at both the GLP‐1 and amylin receptors (Figure 2B,C; Table 1). GLP‐1 receptor activation was measured using Chinese hamster ovary (CHO) cells stably expressing the human GLP‐1 receptor, while amylin receptor activity was assessed in cAMP Hunter cells expressing the AmyR3 heterodimer [10, 39]. Such cAMP accumulation assays are widely used functional readouts for assessing GLP‐1R agonist potency and amylin receptor activation, and are therefore appropriate for evaluating the agonist activity of the final GLP‐1/amylin constructs. Human GLP‐1(7–37) and davalintide were used as positive controls for GLP‐1R and Amy3R activation, respectively, while DMSO served as the negative control. Davalintide was selected as the Amy3R positive control because it is a well‐established amylin receptor agonist. Selectivity control experiments using the individual parent agonists, as well as a 1:1 mixture of compounds **5** and **7**, confirmed that the observed cAMP responses were driven primarily by the corresponding receptor‐specific agonist component (Figure S22, Supporting Information Section 5.1). Overall activity remained within a pharmacologically relevant range, underscoring the scaffold's ability to support dual engagement (see Supporting Information Section 3.1). Compound **8** demonstrated potent and balanced dual agonism, with GLP‐1 receptor activity closely matching that of compound **5**. Compounds **9–11, 13,** and **14** also retained activity, with EC_50_ values ranging from 4.99 pM (**13**) to 24.2 pM (**9**; Table 1). To demonstrate the feasibility of attaching a third peptide agonist, a second amylin agonist was conjugated to yield compound **14**. Notably, compound **14** displayed strong and balanced activation of both receptors, indicating that fine‐tuning agonist valency can further optimise receptor‐specific responses.

**TABLE 1:** cAMP accumulation receptor assay results. CI stands for confidence interval. See Supporting Information Section 1.3 for full structures.

Entry	Compound	Further compound description	Receptor	EC_50_ (pM)	95% CI (pM)
*1*	**5**	GLP‐1 receptor agonist peptide	GLP‐1	4.47	3.29 – 6.02
*2*	**8**	DualAgo	GLP‐1	6.34	4.82 – 8.38
*3*	**9**	DualAgo with a lipid tag	GLP‐1	24.2	18.3 – 31.9
*4*	**10**	DualAgo with a PEG tag	GLP‐1	13.8	10.9 – 17.4
*5*	**11**	DualAgo with a biotin tag	GLP‐1	7.33	5.64 – 9.56
*6*	**13**	DualAgo with a DOTACu tag	GLP‐1	4.99	4.34 – 5.75
*7*	**14**	DualAgo with a 2:1 amylin‐to‐GLP‐1 ratio	GLP‐1	6.07	4.99 – 7.39
*8*	**7**	Amylin receptor agonist peptide	Amylin	3.23	1.97 – 5.33
*9*	**8**	DualAgo	Amylin	11.1	9.3 – 13.3
*10*	**9**	DualAgo with a lipid tag	Amylin	21.6	18.5 – 25.1
*11*	**10**	DualAgo with a PEG tag	Amylin	17.6	14.3 – 21.6
*12*	**11**	DualAgo with a biotin tag	Amylin	9.90	8.82 – 11.1
*13*	**13**	DualAgo with a DOTACu tag	Amylin	8.77	7.00 – 10.9
*14*	**14**	DualAgo with a 2:1 amylin‐to‐GLP‐1 ratio	Amylin	7.09	5.96 – 8.46

Interestingly, however, compound **14** did not exhibit higher potency than compound **8**, despite incorporating an additional amylin receptor agonist. This outcome may reflect steric constraints arising from the size and structural complexity of the AmyR3 heterodimer, which could limit the ability of **14** to engage both receptors simultaneously or to induce the rearrangements required for optimal activation. Compound **9**, which includes a lipid moiety, showed slightly reduced activity at both receptors. This slight shift in potency likely reflects albumin‐binding effects rather than a loss of intrinsic receptor affinity. Given that benchmarks like semaglutide show significant potency shifts (20–40‐fold) in protein‐rich media [39], the presence of 0.1% bovine serum albumin (BSA) in the assay's medium likely accounts for the 3–5‐fold reduction in observed activity for this lipidated analogue. Compound **10** maintained GLP‐1 receptor potency while showing a modest reduction at the amylin receptor, a trend that may reflect the influence of PEGylation on receptor access (Table 1).

Overall, these results confirm that the modular scaffold is highly effective in generating potent dual agonists with receptor activity profiles, while offering the flexibility to incorporate a wide range of functional modifications. Internalisation of fluorescently labelled compound **12** in GLP1R‐CHO cells was assessed, using wild‐type CHO as a negative control (Figure 3).

**FIGURE 3:**
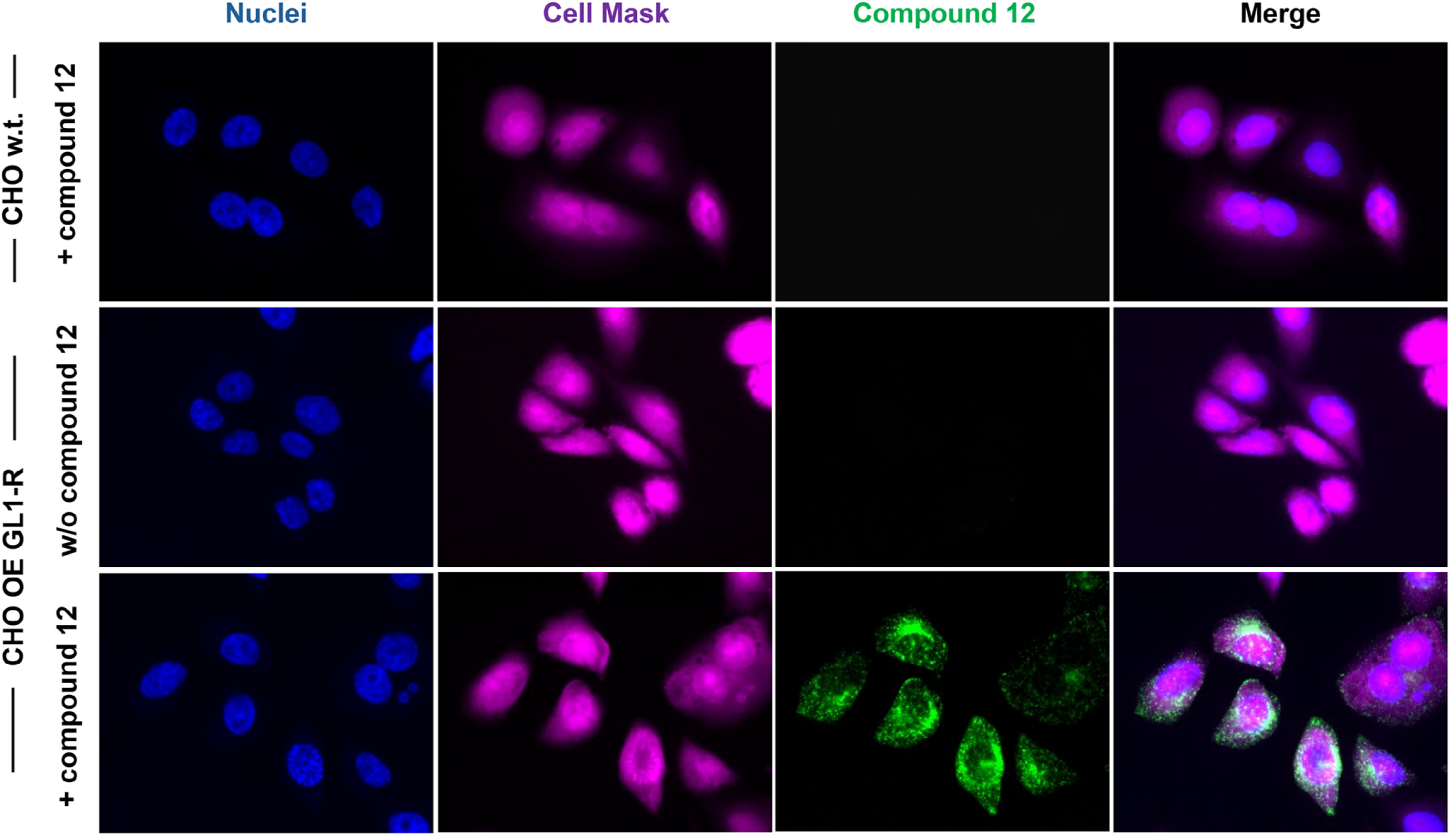
Fluorescent microscopy analysis of receptor‐dependent cellular uptake of compound **12**. TAMRA‐labelled compound **12** showed a clear cell‐associated fluorescent signal in CHO‐GLP1R cells, whereas minimal signal was observed in CHO WT cells. Images are representative 2D confocal fluorescence micrographs used for qualitative assessment of receptor‐dependent cellular uptake. CHO = Chinese hamster ovary cells, WT = Wild‐type, OE = Overexpressing.

Compound **12** (visualised in green) was rapidly and selectively internalised by GLP1R‐CHO cells, with no discernible uptake in wild‐type cells, confirming receptor‐dependent uptake. Blue nuclear and pink membrane staining confirmed its intracellular localisation. These findings complement the cAMP potency data by confirming GLP‐1 receptor–mediated cellular trafficking of the modular scaffold bearing a payload. This observation is particularly important given that our platform was designed for late‐stage functionalisation, enabling the modular incorporation of diverse functional moieties. Demonstrating receptor‐dependent internalisation therefore provides a critical proof‐of‐concept for downstream applications such as targeted cellular imaging and intracellular drug delivery. This establishes a strong foundation for subsequent optimisation towards productive cytosolic delivery. Overall, these results show that the multi‐agonist scaffold efficiently exploits GLP‐1 receptor–mediated endocytosis, underscoring the potential of this modular platform to deliver functional cargos selectively into target cells through receptor‐guided uptake mechanisms [40].

## 3 Conclusion

In summary, a robust PEG‐based modular scaffold **4** was developed, enabling the orthogonal conjugation of up to three peptide or functional payloads through sequential SPAAC and CuAAC reactions. The solid‐phase synthesis of **4** was achieved rapidly, without intermediate purification, and with high crude purity and broad tolerance for diverse chemical modifications. Through this approach, multi‐agonist constructs were assembled combinatorially from pre‐synthesized peptides, eliminating the need for repeated synthesis of large fusion sequences.

When applied to dual GLP‐1 and amylin receptor agonists, the platform yielded a panel of constructs displaying potent activity at both receptors (EC_50_ values in the low‐picomolar range). Compound **8** highlights the platform's capability to generate potent dual agonists. The modularity of the design allowed systematic interchange of peptide and non‐peptide payloads without altering the core scaffold. For example, conjugation of a lipid moiety in compound **9**, despite a modest decrease in potency, demonstrated the ability of the platform to accommodate pharmacokinetically relevant modifications alongside biologically active or imaging tags. Similarly, compound **14** illustrates how linker branching can be used to alter receptor valency, a feature that is difficult to achieve using conventional synthetic approaches. Selective GLP‐1 receptor‐mediated internalisation was observed for fluorescently labelled construct **12**, indicating potential for targeted intracellular delivery.

Our system introduces several unique capabilities relative to classical unimolecular constructs. First, it decouples peptide design from construct assembly, enabling rapid generation and comparison of dual or triple agonists using identical peptide modules, without the need for complete resynthesis. This format innovation allows fine‐tuning of receptor balance and geometry, facilitating structure–activity relationship studies on signaling bias, potency, and trafficking. Second, it provides tuneable control over receptor activity ratios, permitting systematic exploration of valency effects, which is an aspect difficult to achieve in unimolecular systems where receptor stoichiometry is hardwired. Third, the modular platform supports late‐stage functionalization with pharmacokinetic enhancers (e.g., lipid chains, PEGs) or analytical probes (e.g., biotin, TAMRA, DOTA), enabling parallel optimisation of bioactivity, stability, and imaging potential.

Overall, this modular synthetic strategy offers a rapid and flexible route for constructing and optimising multi‐agonist peptide assemblies. It complements existing unimolecular fusion approaches by enabling systematic exploration of receptor combinations, stoichiometry, and functional payloads, ultimately accelerating the development of next‐generation multi‐incretin therapeutics for obesity and related metabolic diseases. Further application of the concept to other incretin‐based therapies is currently a subject of our research.

## Conflicts of Interest

The authors declare no conflicts of interest.

## Supporting information



The authors have cited additional references within the Supporting Information [36, 43, 44, 45].

## Data Availability

The data that supports the findings of this study are available in the supplementary material of this article.

## Endnotes

^1^
Peptide structures have been generated with AlphaFold DB by swapping any unnatural amino acids for their natural equivalents and drawn by ChimeraX [41, 42]. The linker cartoons were created in BioRender (https://BioRender.com/gwhb1a5).

^2^
According to the International Council for Harmonisation's guideline on Elemental Impurities (R3).
