# Supplementary material for: Peptide Marriages: Modular Assembly of Multi‐Agonist Therapeutics
Source: Chemistry. 2026 Jun 18;32(37):e71280. doi: 10.1002/chem.71280 (PMC13628533; doi:10.1002/chem.71280)
Supplement: Supplementary file 1 [file CHEM-32-e71280-s001.pdf]

Supporting Information

Peptide Marriages: Modular Assembly of Multi-Agonist Therapeutics

Kristina A. Kostadinova, Jan L. Venne, Sona Krajcovicova, James Dodgson, Wouter F. J. Hogendorf, Thomas E. Nielsen, David Hymel, Hannah Bolt, Andie Collinson, Jason Day, Jefferson Revell, and David R. Spring

Table of Contents

1. Experimental Procedures 3

1.1. General Information 3

1.2. Instrumentation 4

1.3. Manuscript related full peptide structures 6

1.4. Experimental Procedures 11

1.4.1. General procedure A: Amide coupling on resin 11

1.4.2. General procedure B: Manual Fmoc deprotection 11

1.4.3. General procedure C: CuAAC reaction 11

1.4.4. Key branching amine **S1** 12

1.4.5. Synthesis of the modular PEG-based linker **4** 13

1.4.6. Synthesis of peptide **5** (GLP-1R agonist) 15

1.4.7. Synthesis of compound **6** 15

1.4.8. Synthesis of peptide **7** (AmylinR agonist) 16

1.4.9. Synthesis of DualAgo **8** 16

1.4.10. Synthesis of lipidic chain **S9** 17

1.4.11. Synthesis of final compound **9** 19

1.4.12. Synthesis of final compound **10** 19

1.4.13. Synthesis of final compound **11** 20

1.4.14. Synthesis of final compound **12** 20

1.4.15. Synthesis of DOTA tag **S11** 21

1.4.16. Synthesis of final compound **13** 22

1.4.17. Synthesis of final compound **14** 23

2. Analytical HPLC chromatograms and LRMS analysis 24

2.1. Analytical UV-LCMS-HPLC of crude reaction mixtures 30

3. Compounds stability studies 31

4. ICP-MS analysis 32

5. Biological evaluation 33

5.1. cAMP accumulation results 35

6. References 37

1. Experimental Procedures

1.1. General Information

All reagents were of reagent grade and used without further purification. Solvents and chemicals were purchased from Sigma-Aldrich (US), Acros Organics (BE), Fluorochem (UK) or Iris Biotech (DE). Anhydrous solvents were stored as received from commercial suppliers. Software used: chemical structures (ChemDraw), UV-LCMS (MassLynx), analytical HPLC (Prism), figures (MS PowerPoint and BioRender), citations (Zotero).

Reactions in solution were performed in round-bottom flasks fitted with rubber septa under positive pressure of nitrogen, in ace-pressure tubes (up to 10 mL) or in conical vials (up to 4 mL). All reactions were monitored by UV-LCMS analysis. Thin-layer chromatography (TLC) plates using aluminium plates precoated with silica gel (silica gel 60 F254, Merck, US) impregnated with a fluorescent indicator were visualised by exposure to ultraviolet light (λ = 254 nm). Column chromatography was performed using silica gel (60 Å, 230–400 mesh, Sigma-Aldrich).

Automated peptide synthesis was carried out on a CEM Liberty Automated Microwave Peptide Synthesiser using Rink Amide resin (loading 0.42 mmol/g). The scale for resin-bound compounds was 0.1 mmol. All automated amide couplings were performed with Fmoc-protected amino acids (0.2 M), HOBt (1 M) and DIC (1 M) as coupling reagents in DMF. After automated synthesis all resin-bound compounds were transferred to plastic syringes each equipped with a porous disk, washed with CH_2Cl_2 (5 × 10 mL), DMF (2 × 10 mL) and CH_2Cl_2 again (2 × 10 mL) and stored dried in a freezer. Fmoc deprotection was performed with 20% piperidine in DMF. Protecting groups used during solid-phase peptide synthesis: *t*-Bu (Asp, Glu, Tyr, Ser, Thr); Boc (Lys, His, Trp); Pbf (Arg); Trt (Gln, Asn).

For manual solid-phase synthesis reactions were carried out in plastic reaction vessels (syringes, each equipped with a porous disk from Torviq, US, www.torviq.com), using 2-chlorotrityl chloride resin pre-modified with H-Gly-OH (loading 0.63 mmol/g) or Rink Amide resin (0.36 mmol/g). All external reagents were dissolved in a glass beaker in an appropriate solvent and added to the resin at once, unless stated otherwise. The volume of used solvent was 10 mL per 1 g of resin, unless stated otherwise. All manual solid-phase reactions were performed at ambient temperature, unless stated otherwise. Resin slurry has been shaken on a shaker at 400 – 600 rpm. For manual washing, resin slurry was shaken with the fresh solvent for at least 1 min before changing the solvent. UV-LCMS analysis was performed after each reaction step (except for Fmoc cleavage) to ensure completeness of the synthesis, unless stated otherwise.

Upon solid-phase synthesis, UV-LCMS analysis was performed as follows: analytical sample of a resin (~5 mg) was transferred into a plastic Eppendorf tube, treated with a cleavage cocktail (**Rink amide:** TFA, 1 mL; **2-Cl-Trt:** CH_2Cl_2/HFIP 2:1, 1.5 mL, v/v; unless stated otherwise) and shaken for 1–3 hours at ambient temperature. For peptides containing a trityl protecting group, 100 µL of TIPS was added to a cleavage cocktail with TFA as a quencher. The cleavage cocktails were then evaporated

under a stream of nitrogen, cleaved compounds extracted into DMSO, filtered, and submitted to UV-LCMS analysis to determine purity of the prepared compounds.

For full cleavage of linker 4 from the resin a cleavage cocktail of CH_2Cl_2/HFIP (2:1, 20 mL, v/v) was used and the cleavage time was prolonged to 4–5 hours (usually 2 × 2-hour cycles were performed). Cleavage cocktails were evaporated under reduced pressure, and crude product was carried on without further purification.

For the full cleavage of peptides from the resin prior to HPLC purification a cleavage cocktail of TIPS/TFA/H_2O (0.25:4.5:0.25 v/v, 30 mL; unless stated otherwise) was used.

1.2. Instrumentation

UV-LCMS analysis was carried out using Waters ACQUITY H-Class UPLC with an ESCi Multi-Mode Ionisation Waters SQ Detector 2 spectrometer using ACQUITY UPLC® CSH C18 (2.1 mm × 50 mm, 1.7 µM, 130 Å) column at 40 °C and flow rate 0.6 mL/min. Mobile phase was (A) 2 mM ammonium acetate in water/MeCN (95:5), (B) MeCN and (C) 2% aq. formic acid, linearly programmed from 5–95% B over 5 min with constant 5% C over 1 min. The ESI source operated at a discharge current of 5 µA, vaporiser temperature of 350 °C and capillary temperature of 200 °C. Chromatographs were monitored by UV absorbance using a photodiode array detector at a wavelength range of 210–800 nm, interval 1.2 nm.

NMR spectra were recorded on Bruker Avance III 500 MHz HD Smart Probe with operating frequencies 500.16 MHz (for ^{1}H) and 125.77 MHz (for ^{13}C) at 23 °C, respectively. Chemical shifts (δ) are reported in parts per million (ppm) and coupling constants (*J*) are reported in Hertz (Hz). The ^{1}H and ^{13}C NMR chemical shifts (δ in ppm) were referenced to the residual signals of DMSO-d_6 [2.50 (^{1}H) and 39.52 (^{13}C)]. Abbreviations in NMR spectra: app – apparent, br – broad, d – doublet, dd – doublet of doublets, h – heptet, m – multiplet, me – multiplicity edited, s – singlet, t – triplet.

Preparative HPLC was carried out on an Agilent 1260 Infinity using a Supelcosil ABZ+PLUS column (250 mm × 21.2 mm, 5 µM) eluting with a linear gradient system with mobile phases (A) 0.1% TFA in water (v/v) and (B) 0.05% TFA in MeCN (v/v) over 20 min at a flow rate of 20 mL/min.

Preparative and analytical UV-LCMS-HPLC was carried out on Waters AutoPurification mass-directed HPLC system with photodiode array detector, SQ Detector 2 and at-column dilution. The analytical runs used an X-Select CSH C18 column (4.6 × 150mm, 5 µM particle size) and flow rate of 1.2 mL/min. The preparative runs used an X-Select CSH Prep C18 OBD column (19 × 150mm, 5 µM particle size) and flow rate of 20 mL/min. The machine used a linear gradient of solvent B in solvent A run over 15 min with 1 min isocratic hold before the gradient (analytical) or over 15 min with 1.4 min isocratic hold (preparative). For acidic runs, solvent A was water with 0.1% (v/v) formic acid and solvent B was MeCN with 0.1% (v/v) formic acid. For neutral runs solvent A was water (no additives) and solvent B was MeCN (no additives).

Analytical HPLC was carried out using Agilent 1260 or 1200 Infinity systems with a reversed-phase Supelcosil™ ABZ+PLUS column (150 mm × 4.6 mm, 3 µM), Agilent Eclipse XDB C18, (5 µm, 4.6 x 150 mm) column, or Agilent AdvanceBio Peptide Mapping (120Å pore size, 4.6 x 150 mm, 2.7 µm particle size) column eluting with a linear gradient system (solvent A: 0.05% (v/v) TFA in water, solvent B: 0.05% (v/v) TFA in MeCN) over 15 minutes, at a flow rate of 1 mL/min. Analytical HPLC was monitored by UV absorbance at 220, 254 and 280 nm.

Peptide co-agonist constructs LCMS was performed on a Xevo G2-S TOF mass spectrometer coupled to Waters Acquity UPLC system using an Acquity UPLC BEH300 C4 column (1.7 µm, 2.1 × 50 mm). H_2O with 0.1% formic acid (solvent A) and 95% MeCN and 5% H_2O with 0.1% formic acid (solvent B) were used as the mobile phase at a flow rate of 0.2 mL/min. The gradient was programmed as follows: 95% A for 0.93 min, then a gradient to 100% B over 4.28 min. The column was then washed 100% B for 1.04 minutes, then a gradient to 95% A over 1.04 min. The electrospray source was operated with a capillary voltage of 2.0 kV and a cone voltage of 190 V. Nitrogen was used as the desolvation gas at a total flow of 850 L/h. Total mass spectra were reconstructed from the ion series using the MaxEnt 1 algorithm preinstalled on MassLynx software (v4.2 from Waters) according to the manufacturer's instructions. For each compound, for expected ions, the *monoisotopic* m/z has been calculated for the ion as a sum of the accurate masses of the lightest isotopes of each atom. In addition, due to the large size of the molecules, in many cases, the ion composed of the lightest isotopes would not be the most abundant observed (higher probability of at least one or more ^{13}C to be present in a molecule of the compound).[1] Therefore, a calculated accurate (exact) mass to charge ratio of the predicted most abundant ion is also given. ChemDraw has been used to perform the above calculations.

1.3. Manuscript related full peptide structures

The key structures related to the main manuscript (please see below for experimental procedures and characterisation). To improve clarity, some bonds may have been extended.

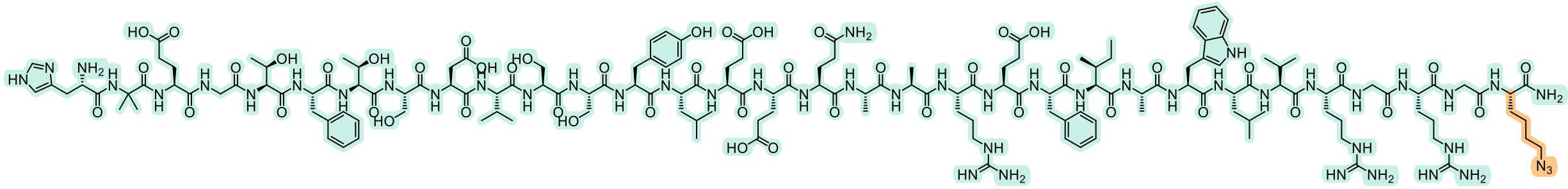

5; GLP-1R agonist

His-Aib-Glu-Gly-Thr-Phe-Thr-Ser-Asp-Val-Ser-Ser-Tyr-Leu-Glu-Glu-Gln-Ala-Ala-Arg-Glu-Phe-Ile-Ala-Trp-Leu-Val-Arg-Gly-Arg-Gly-Lys(N_3)

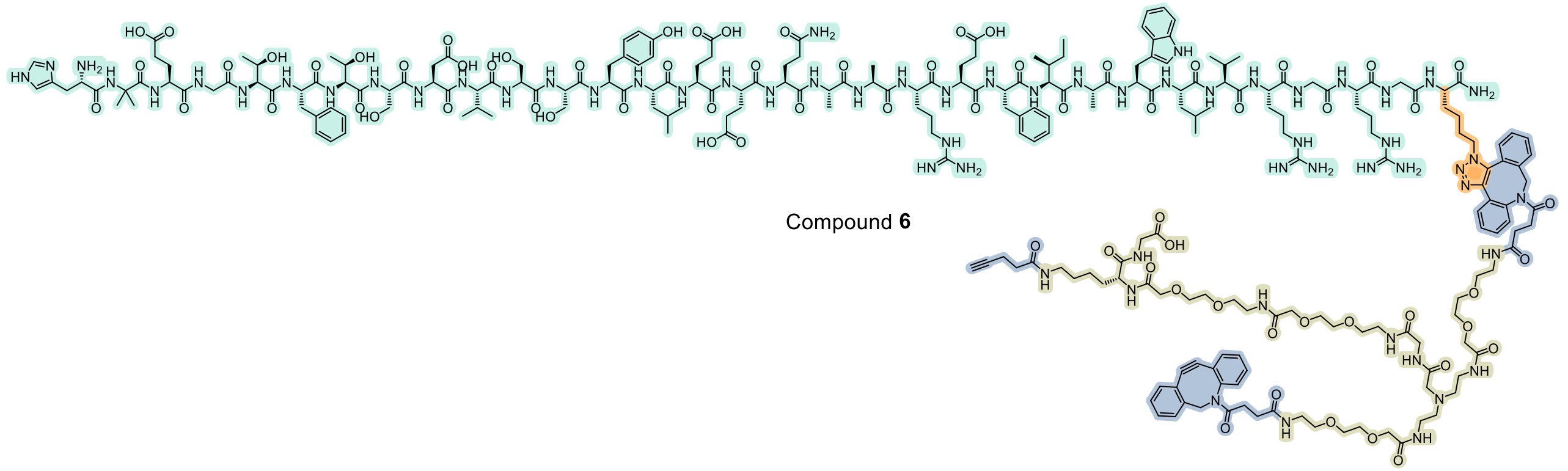

Compound **6**

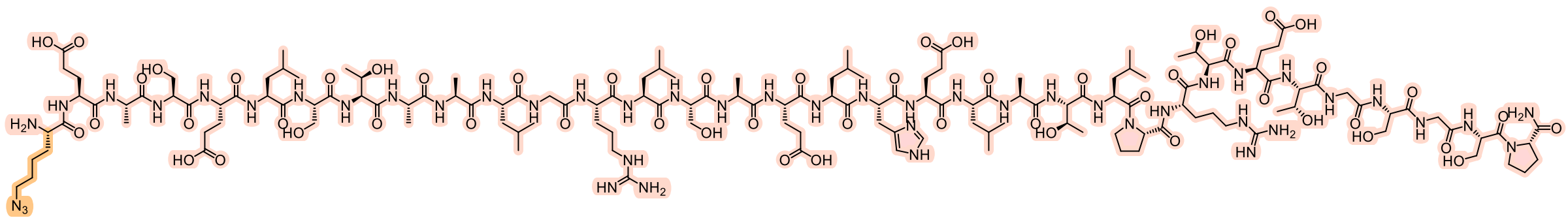

7; AmylinR agonist

Lys(N_3)-Glu-Ala-Ser-Glu-Leu-Ser-Thr-Ala-Ala-Leu-Gly-Arg-Leu-Ser-Ala-Glu-Leu-His-Glu-Leu-Ala-Thr-Leu-Pro-Arg-Thr-Glu-Thr-Gly-Ser-Gly-Ser-Pro

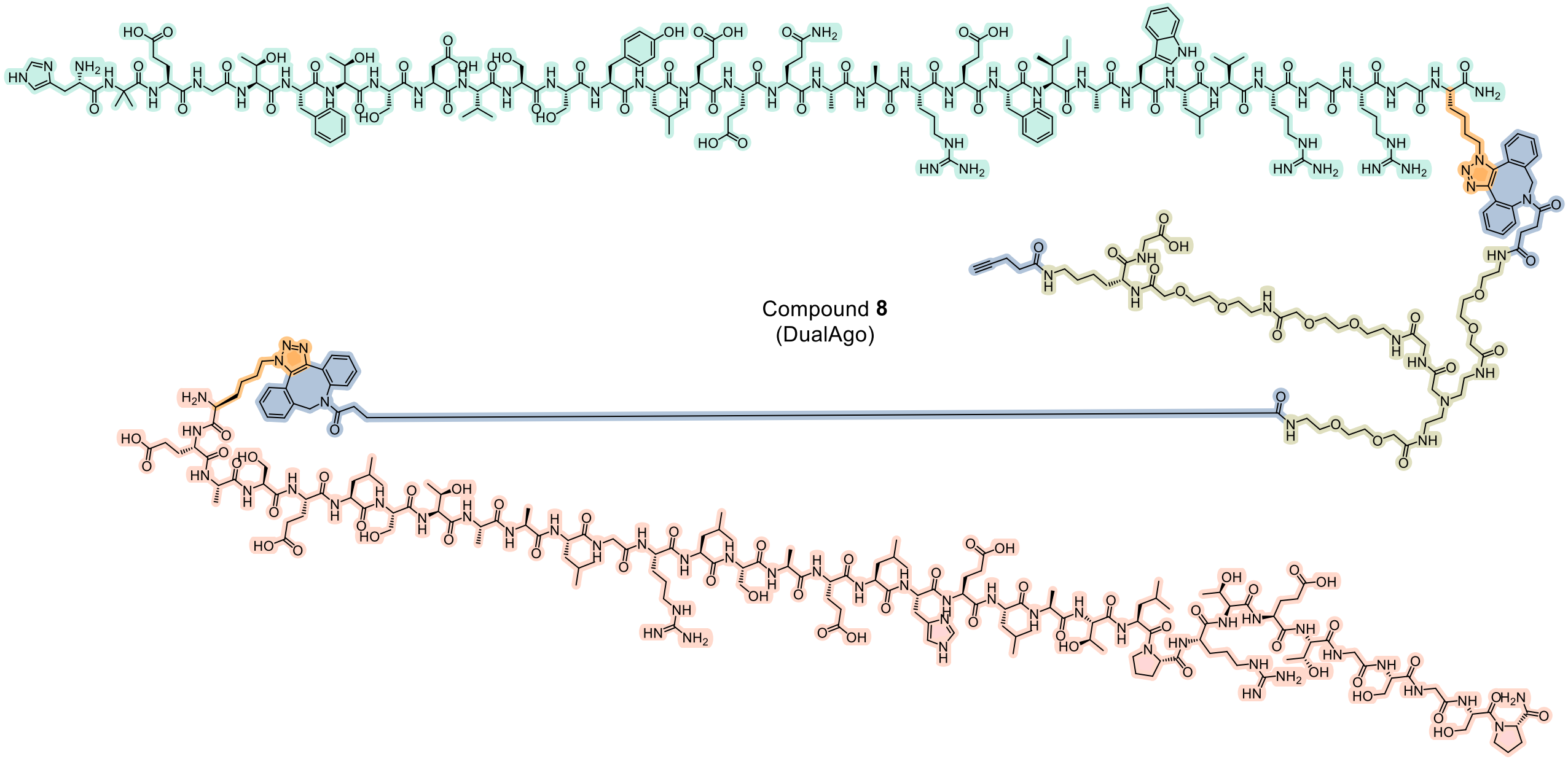

Compound **8** (DualAgo)

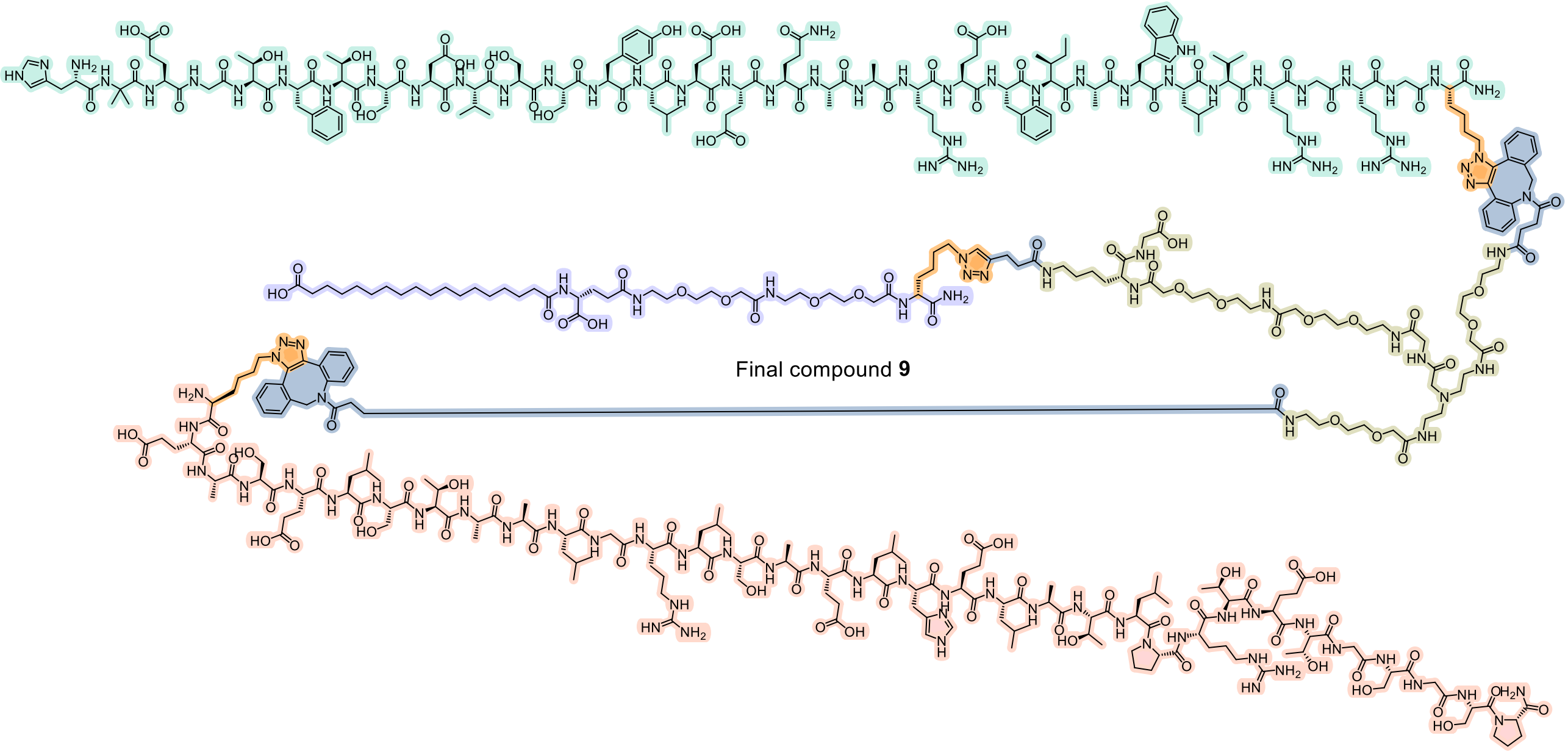

Final compound **9**

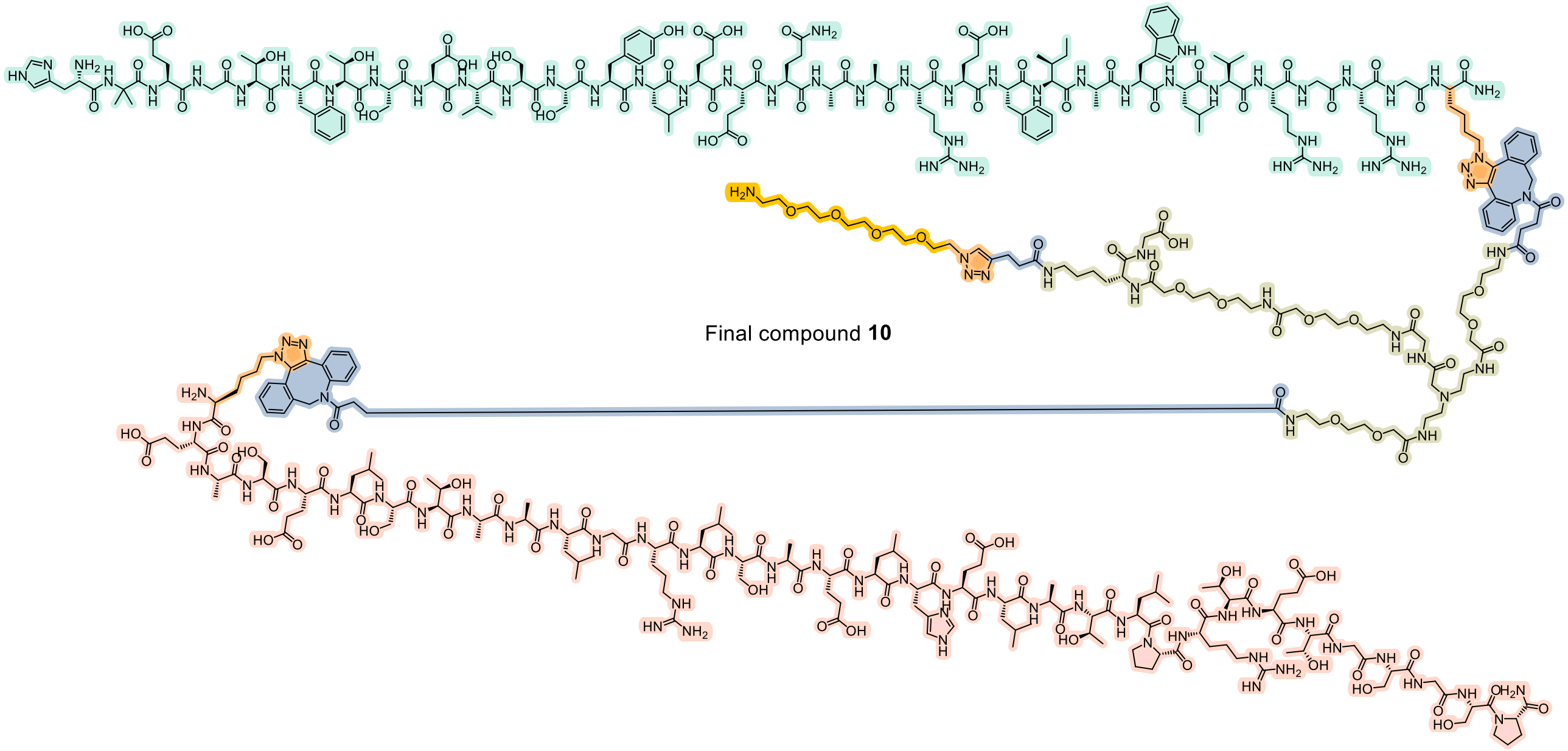

Final compound **10**

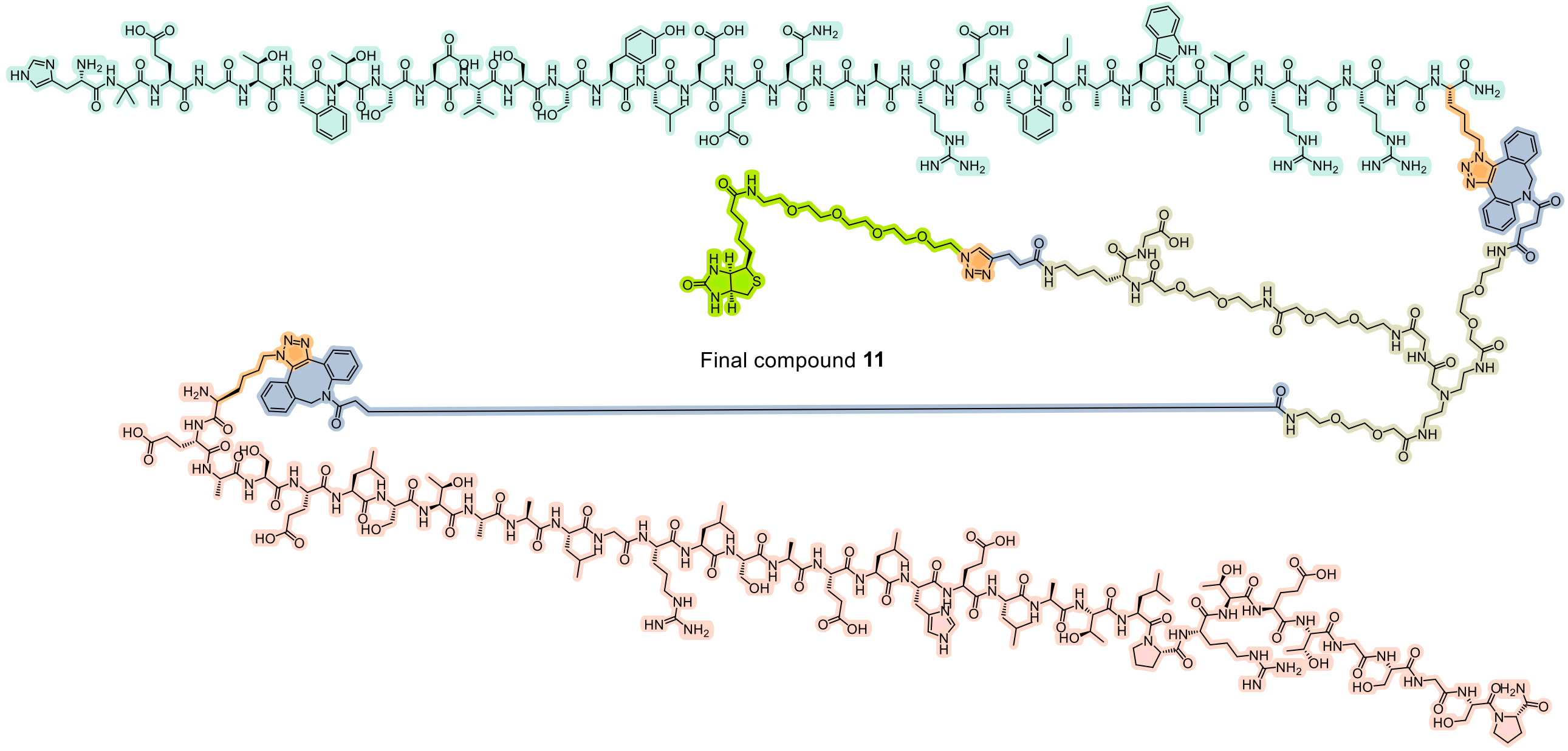

Final compound **11**

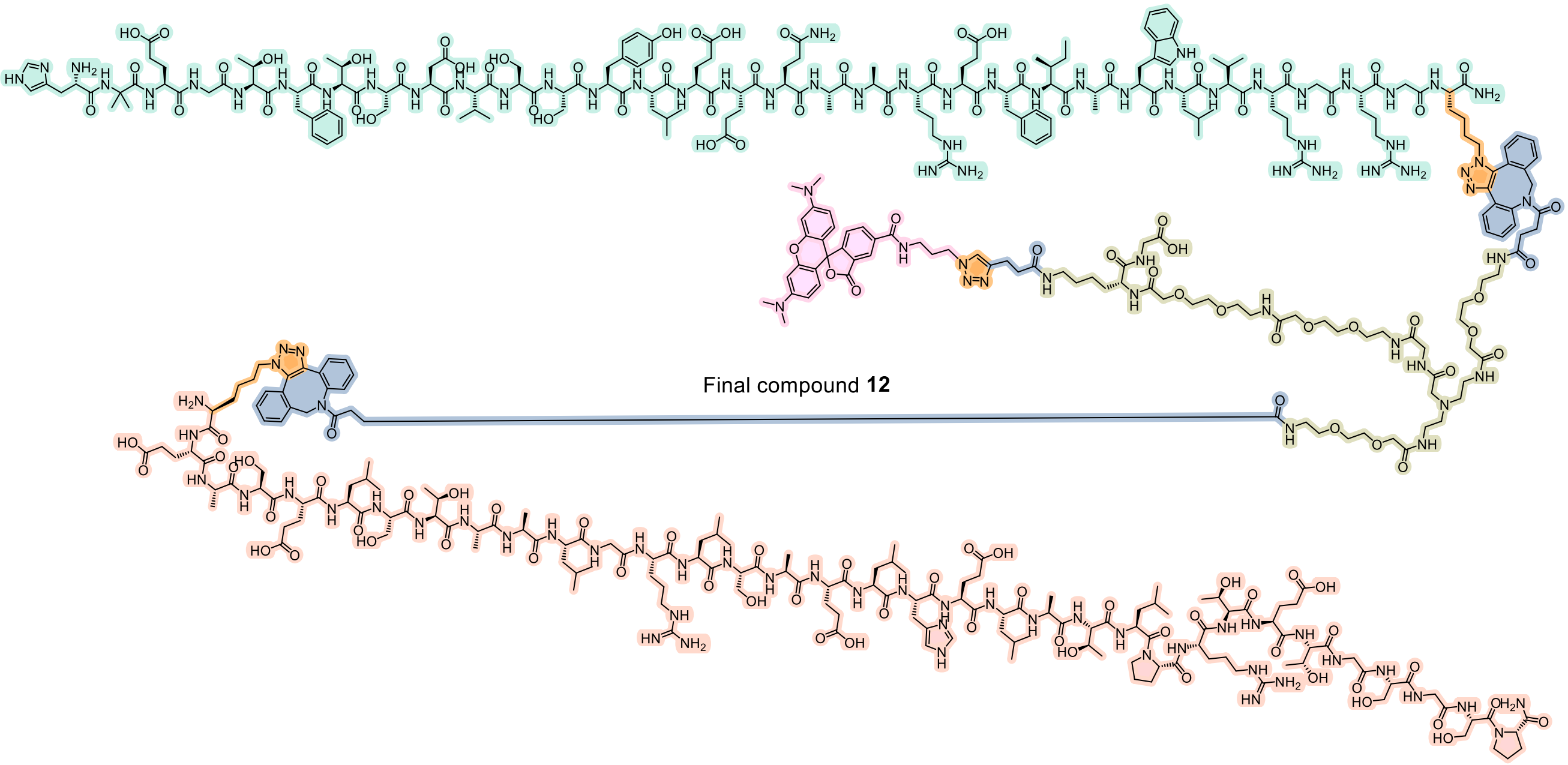

Final compound **12**

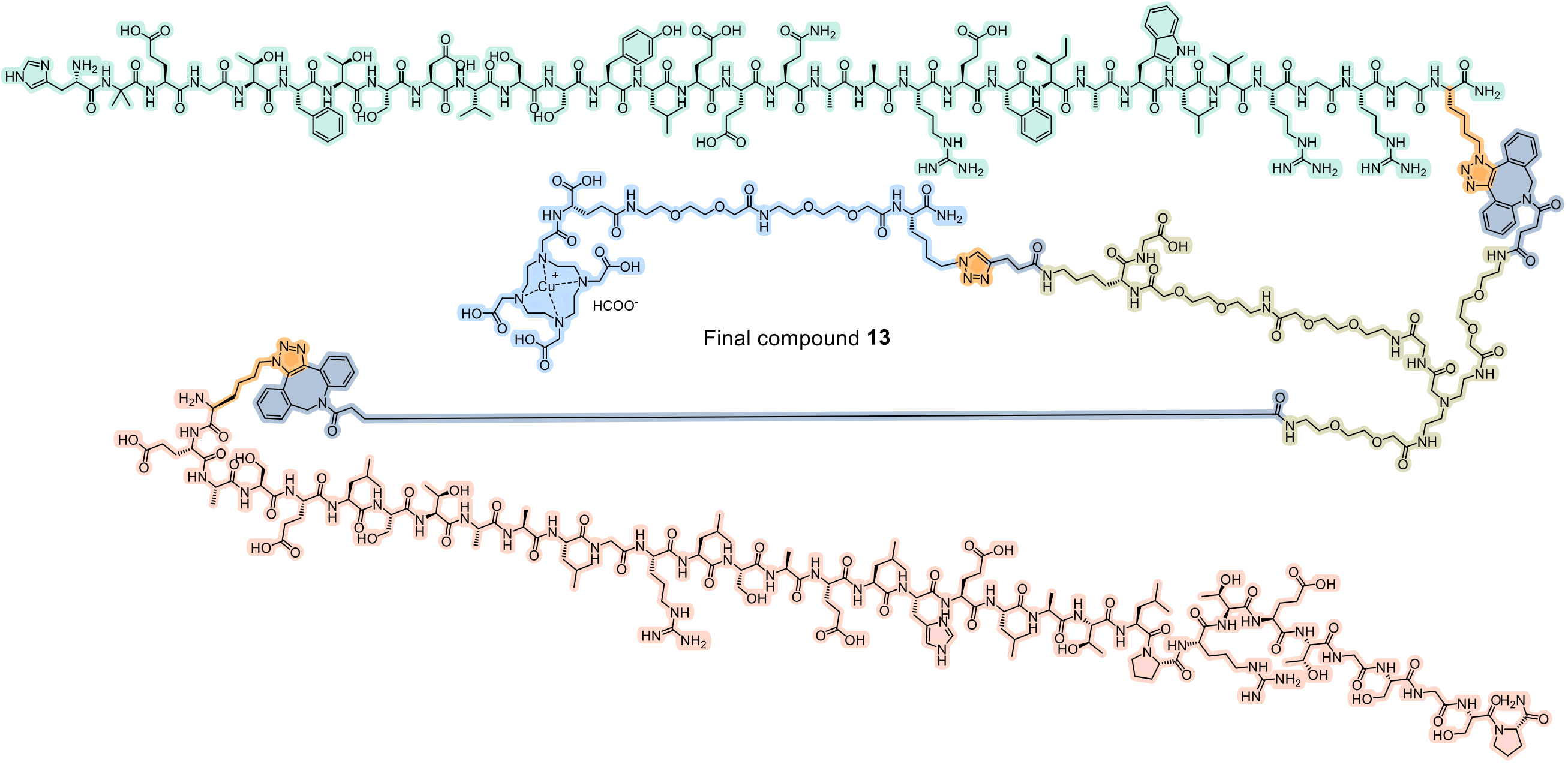

Final compound **13**

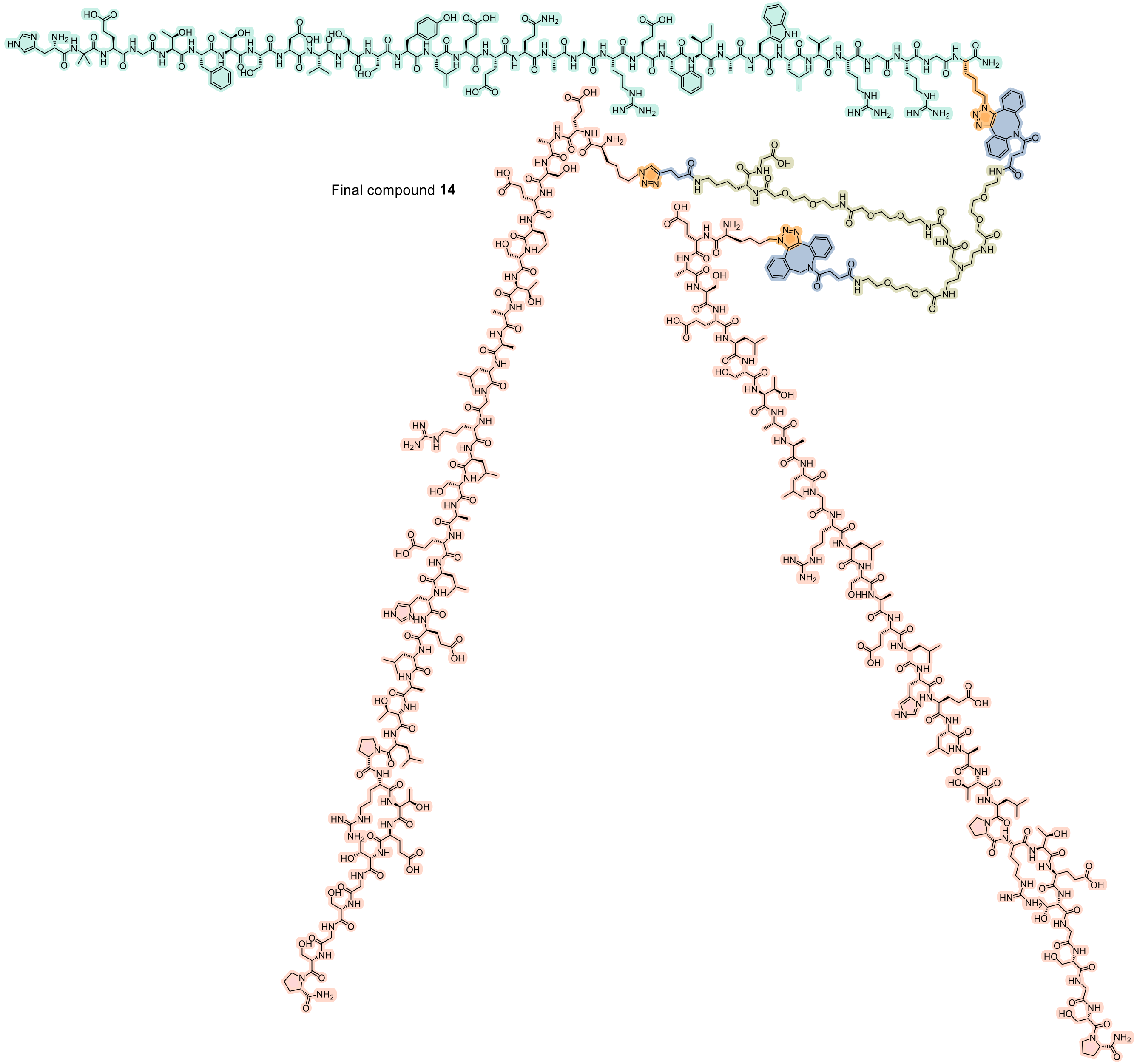

Final compound **14**

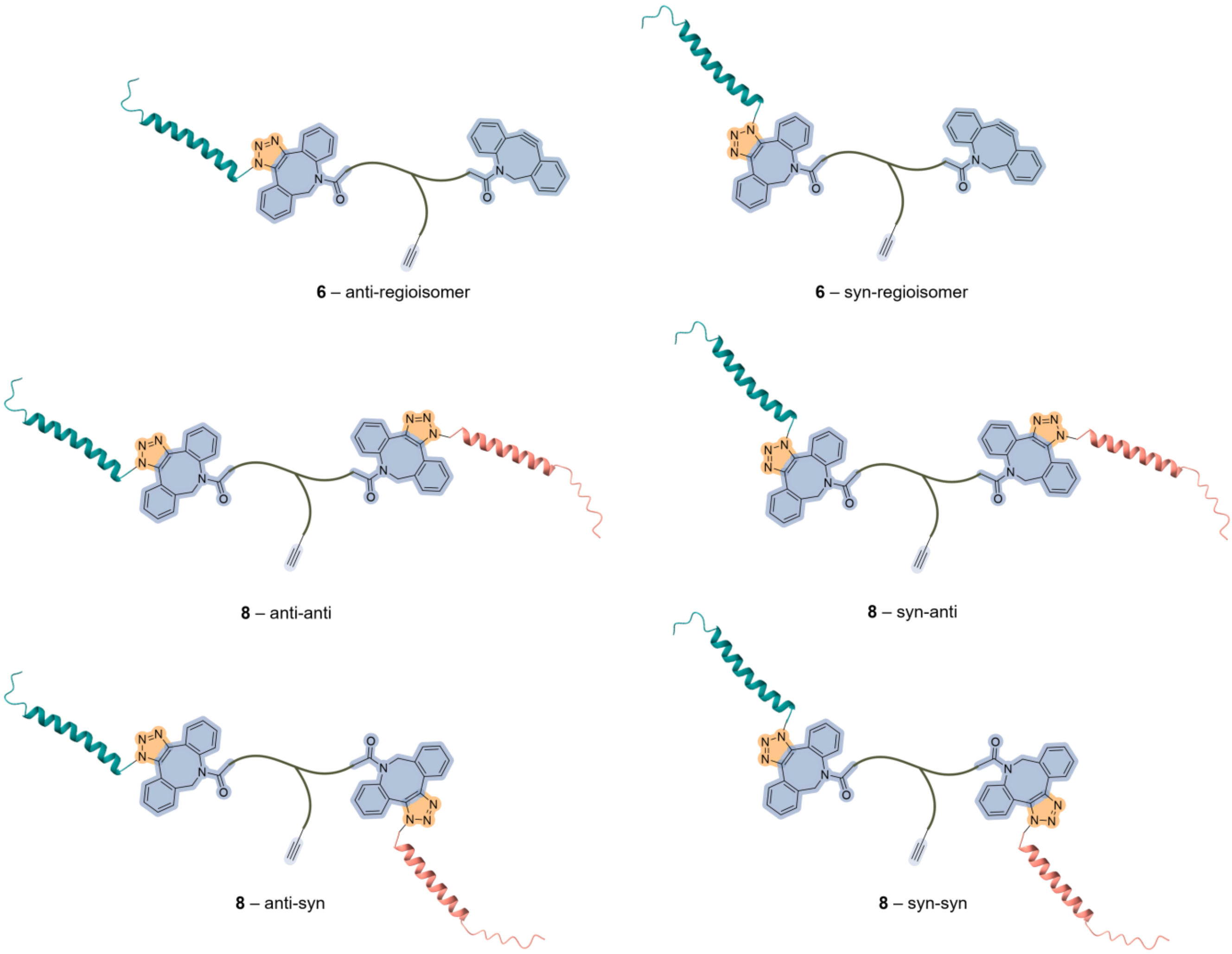

Figure S1. Possible regioisomers of compounds **6** and **8** obtained after SPAAC reaction

1.4. Experimental Procedures

1.4.1. General procedure A: Amide coupling on resin

Corresponding Fmoc deprotected resin was washed with CH_2Cl_2 (3 × 10 mL). Corresponding carboxylic acid and HOBt were dissolved in DMF, followed by addition of DIC prior to the addition to the resin. The reaction mixture was added to a plastic syringe with the resin at once and the slurry was shaken for 1–3 hours. The resin was washed with CH_2Cl_2 (3 × 10 mL), DMF (3 × 15 mL) and CH_2Cl_2 (5 × 10 mL) and small portions were cleaved and analysed by UV-LCMS.

1.4.2. General procedure B: Manual Fmoc deprotection

Corresponding resin was washed with CH_2Cl_2 (3 × 10 mL). Manual Fmoc deprotection was performed with 5% DBU in CH_2Cl_2 in a plastic syringe at ambient temperature for 15 minutes. The resin was washed with CH_2Cl_2 (3 ×10 mL), DMF (3 ×15 mL) and CH_2Cl_2 (5 ×10 mL) and used in the next step without further analysis.

1.4.3. General procedure C: CuAAC reaction

To a solid peptide **8** (1.0 eq.), a 400 µL solution of the respective tag (1.5 eq., unless otherwise stated) in 3:1 DMSO/water was added. The mixture was thoroughly degassed by bubbling nitrogen through it for at least 4 minutes and then stirred. Separately, $CuSO_4{\cdot}5H_2O$ (10 eq.) and tris(3-hydroxypropyltriazolylmethyl)amine (THPTA, 20 eq.) were dissolved in 100 µL of 1:1 water/^{t}BuOH, and the solution was degassed as above. Sodium ascorbate (100 eq.) was then added, and the solution was again degassed thoroughly. This catalyst solution was added to the peptide–tag mixture, and the reaction was stirred at room temperature under an inert atmosphere for 6 hours (unless otherwise stated). The crude product was purified by preparative UV-LCMS-HPLC.

<u>Note:</u> *where small amounts of reagents were required, stock solutions with higher volumes and amounts were prepared.*

1.4.4. Key branching amine S1

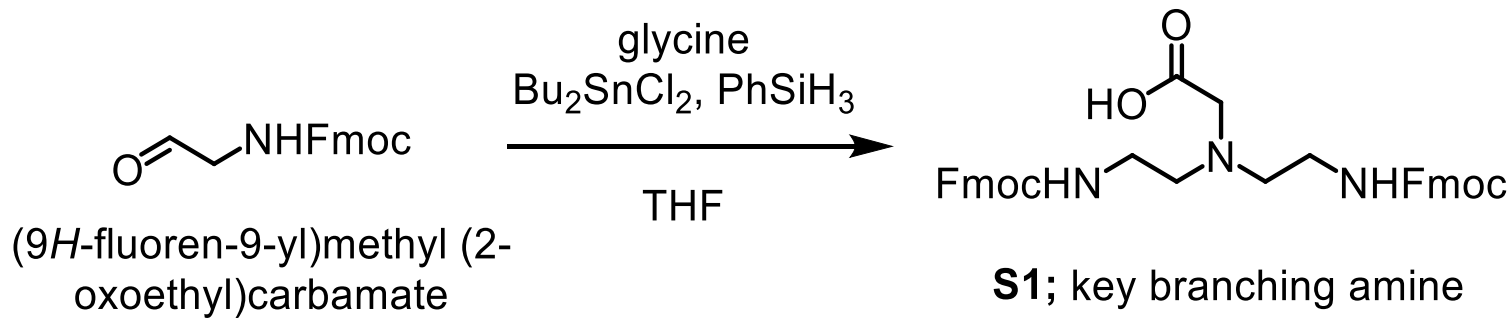

S1 was obtained according to the previously reported procedure.[2]

Briefly, commercially available (9H-fluoren-9-yl)methyl (2-oxoethyl)carbamate (3 g, 10.65 mmol, 5 eq) was dissolved in anhydrous THF (20 mL), followed by addition of glycine (159 mg, 2.13 mmol, 1 eq) and Bu_2SnCl_2 (645 mg, 2.13 mmol, 1 eq). The mixture was vigorously stirred for 20 min at ambient temperature and then phenyl silane (1.31 mL, 10.65 mmol, 5 eq) was slowly added. The reaction mixture was heated up to reflux and stirred for 22 h after which UV-LCMS analysis confirmed full conversion to product **S1**. The mixture was cooled down to ambient temperature, diluted with aq. NH_4Cl (500 mL) and extracted with EtOAc (3 × 600 mL). Organic extracts were combined, dried over $MgSO_4$, filtered and evaporated under reduced pressure. The crude product was purified by column chromatography (CH_2Cl_2/MeOH 12.5:1 to 1:2, v/v) to afford compound **S1** as a white amorphous solid (1.27 g, 2.09 mmol, 98%).

LCMS R_t = 2.45 min (5–95% B over 5 min with constant 5% C over 1 min)

^{1}H NMR (500 MHz, DMSO-d$_6$): δ 7.87 (d, *J* = 7.5 Hz, 4H), 7.66 (d, *J* = 7.5 Hz, 4H), 7.39 (t, *J* = 7.5 Hz, 4H), 7.30 (td, *J* = 7.5, 1.2 Hz, 4H), 7.22 (t, *J* = 5.7 Hz, 2H), 4.27 (d, *J* = 7.1 Hz, 4H), 4.19 (t, *J* = 7.0 Hz, 2H), 3.31 (s, 2H), 3.06 (q, *J* = 6.4 Hz, 4H), 2.67 (t, *J* = 6.7 Hz, 4H) ppm.

^{13}C NMR (126 MHz, DMSO-d$_6$): δ 173.1, 163.5, 156.6, 144.3, 141.1, 128.0, 127.5, 125.5, 120.5, 65.8, 53.8, 47.1 ppm.

All analytical data agree with that previously reported.[2]

1.4.5. Synthesis of the modular PEG-based linker 4

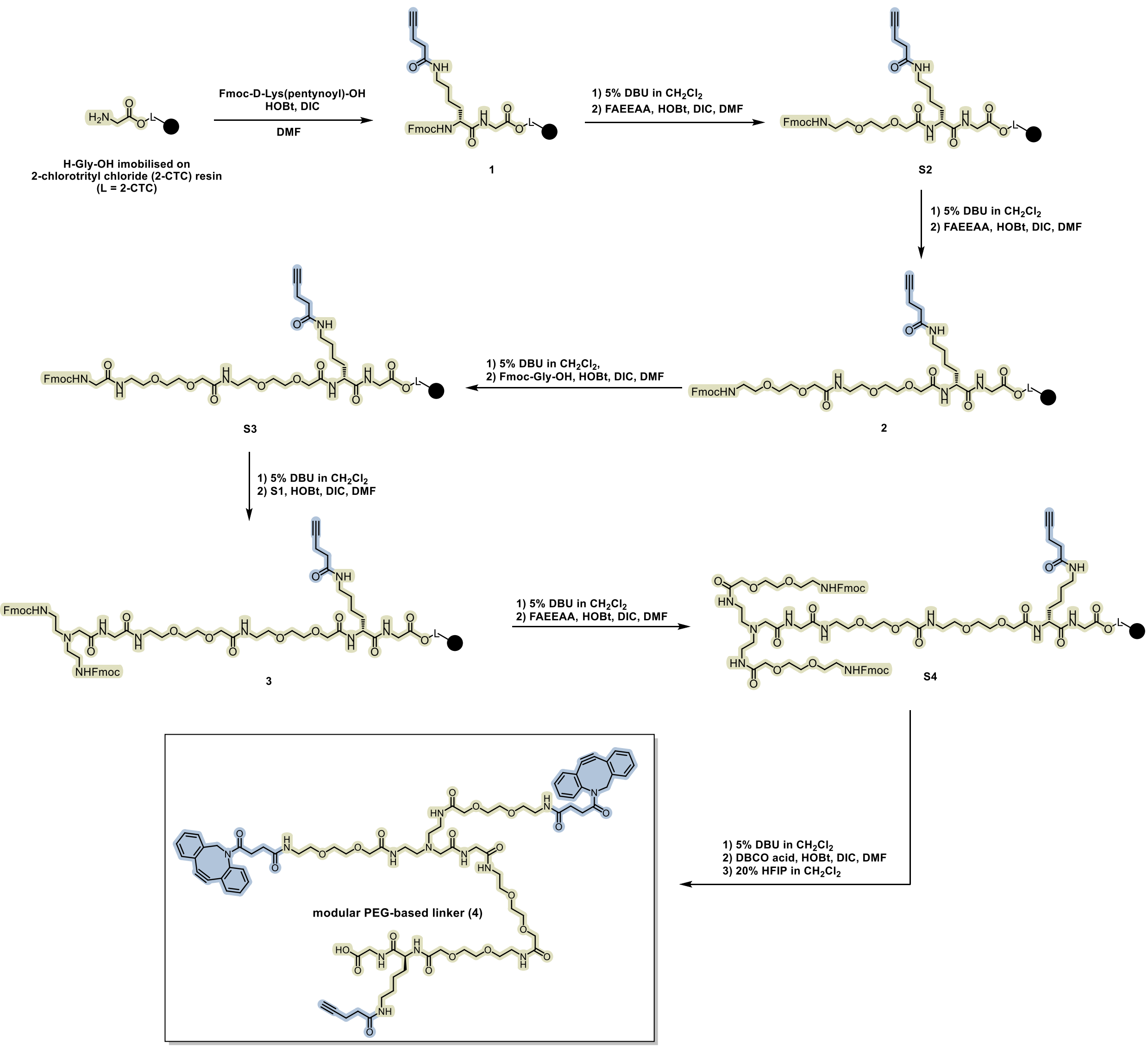

Table S1. Reagents table for linker 4 solid-phase synthesis

Cmpd.	Carboxylic acid used	CAS (carboxylic acid)	MW	Conc. [M]	n [mmol]	m [mg]	Calcd. *m/z* [M+H]+
**1**	Fmoc-D-Lys(pentynoyl)-OH	2576508-18-2	448	0.2	0.6	269	506.2
**S2**	{2-[2-(Fmoc-amino)ethoxy]ethoxy}acetic acid (FAEEAA)	166108-71-0	385	0.2	0.6	231	652.3
**2**	FAEEAA	166108-71-0	385	0.2	0.6	231	796.3
**S3**	Fmoc-Gly-OH	29022-11-5	297	0.2	0.6	178	853.9
**3**	**S1**	-	605	0.2	0.6	363	1218.5
**S4**	FAEEAA	166108-71-0	385	0.2	0.6	231	1508.7
**4**	DBCO acid	1353016-70-2	305	0.2	0.6	183	1638.7

Resin scale 250 mg (H-Gly-OH 2-CTC, loading 0.63 mmol/g). Compounds have been prepared according to the [*1.3.1. General Procedure **A** (amide coupling)*] and [*1.3.2. General Procedure **B** (manual Fmoc deprotection).*] Intermediates have been analysed after every amide coupling by small scale cleavage by CH_2Cl_2/HFIP 4:1 (see *1.1. General information*) to determine purity. DBCO acid, albeit commercially available, has been prepared according to the previously published procedure.[3] For all

amide couplings have been used HOBt (0.2 M; 0.6 mmol, 92 mg), DIC (0.2 M; 0.6 mmol, 91 µL), and DMF (3 mL). Overall yield of **4** from H-Gly-OH 2-CTC: 125 mg, 0.076 mmol, 48%.

- ***Crude UV-LCMS traces of linker intermediates (upon cleavage from the resin)***

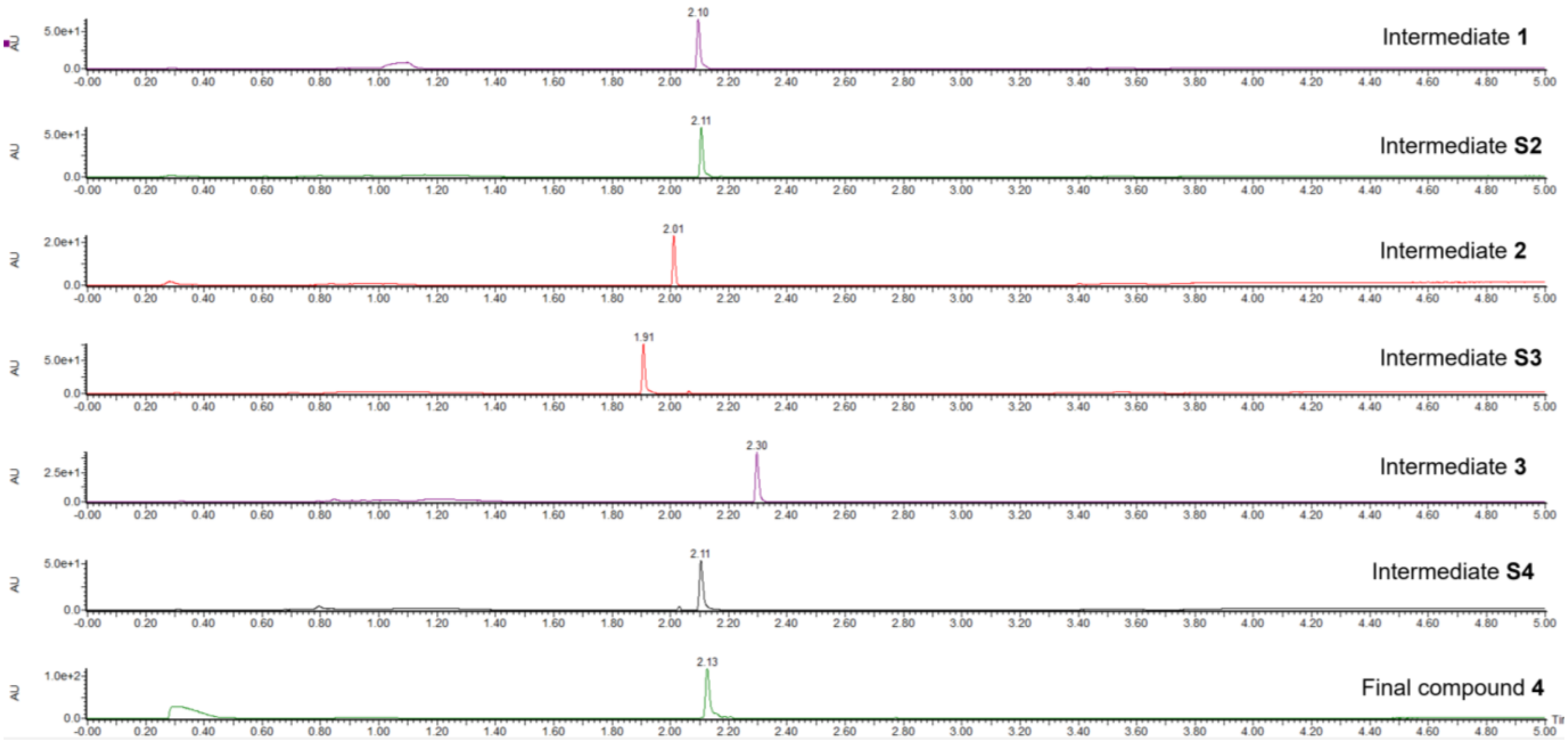

Figure S2

- ***LRMS traces of linker intermediates (upon cleavage from the resin)***

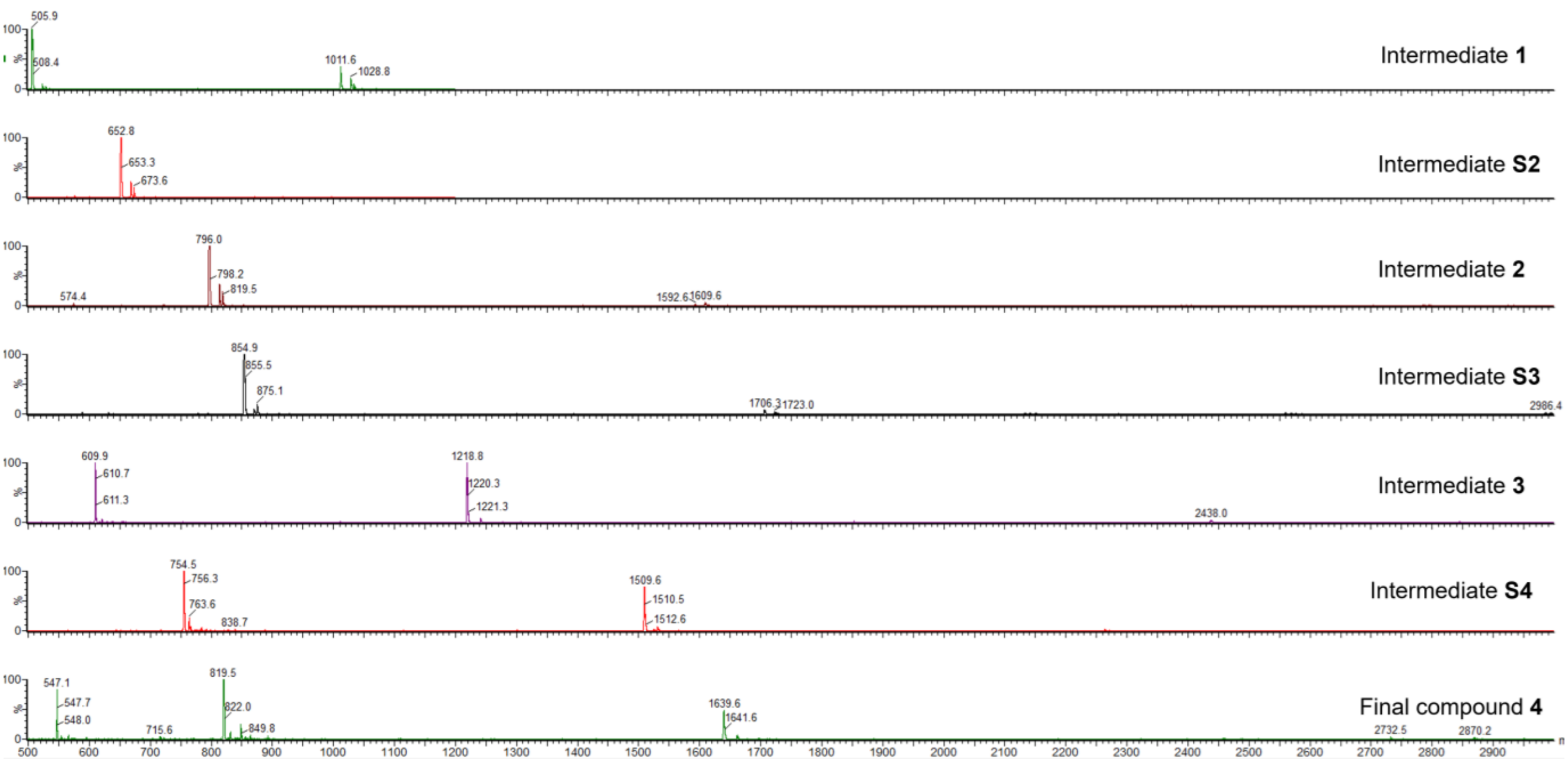

Figure S3

1.4.6. Synthesis of peptide 5 (GLP-1R agonist)

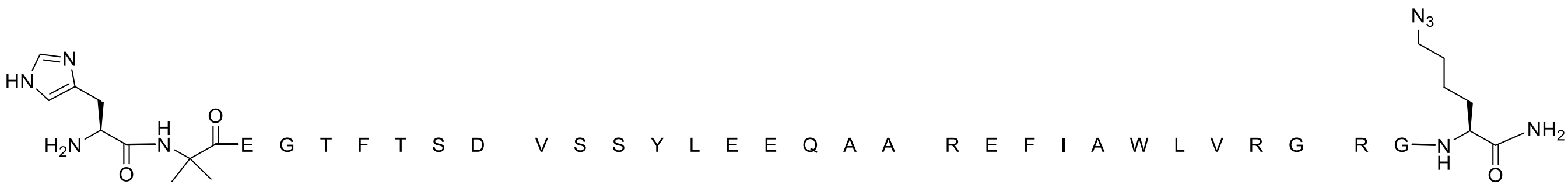

5; GLP-1R agonist

Compound **5** has been obtained by automated peptide synthesis as described above (*1.1. General information*) as a white solid (83 mg, 23%).

Analytical UV-LCMS-HPLC R_t = 7.07 min (5–95% B over 15 min)
Purity: 69%, determined by analytical UV-LCMS-HPLC at 220 nm
LRMS compound molecular formula (M): $C_{161}H_{245}N_{49}O_{49}$
m/z calcd. for $[M+4H]^{4+}$: monoisotopic 913.2119, predicted most abundant 913.4628, ***m/z* found for $[M+4H]^{4+}$: 913.7**

1.4.7. Synthesis of compound 6

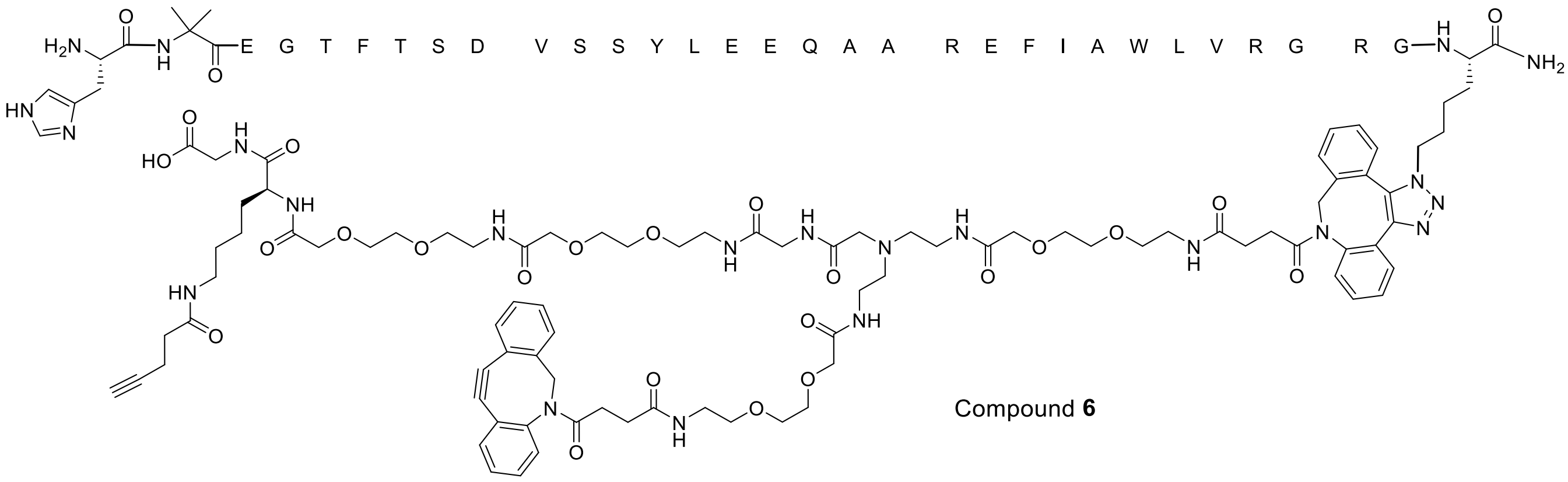

Compound **6**

To a solution of linker **4** (7.0 mg, 4.3 µmol, 2.5 eq.) in anhydrous DMSO (500 µL) was added dropwise a solution of GLP-1R agonist **5** (6.24 mg, 1.7 µmol) pre-dissolved in anhydrous DMSO (500 µL). The reaction mixture was stirred at room temperature under an inert atmosphere for 30 minutes. The crude product was purified by preparative UV-LCMS-HPLC (gradient 25–55% solvent B). Collected fractions were immediately frozen and lyophilised to afford peptide **6** (2.94 mg, 33%) as a white solid, obtained as a mixture of regioisomers (Figure S1).[4] <u>*Note:*</u> *Delayed freezing and lyophilisation may lead to hydrolysis of the DBCO moiety in the product.*

Analytical HPLC R_t = 9.43 min (5–95% B over 15 min),
Purity: 80%, determined by analytical HPLC at 220 nm
LRMS compound molecular formula (M): $C_{244}H_{352}N_{62}O_{71}$
m/z calcd. for $[M+5H]^{5+}$: monoisotopic 1058.3241, predicted most abundant 1058.7254, ***m/z* found for $[M+5H]^{5+}$: 1058.9**
m/z calcd. for $[M+H]^{+}$: monoisotopic 5287.5912, predicted most abundant 5289.5980, ***m/z* deconvoluted for $[M+H]^{+}$: 5288**

1.4.8. Synthesis of peptide 7 (AmylinR agonist)

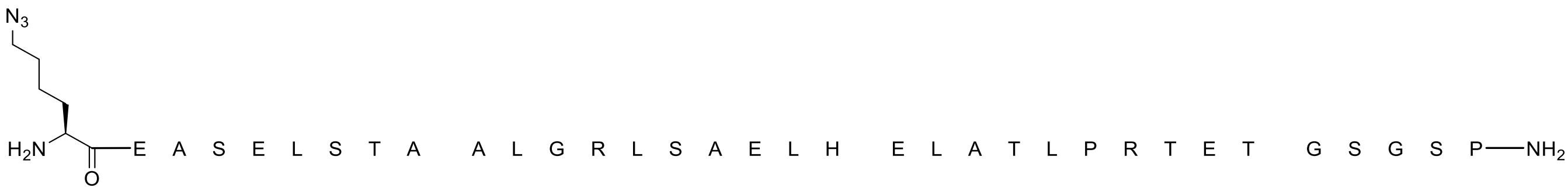

7; AmylinR agonist

Compound **7** has been obtained by automated peptide synthesis as described above (*1.1. General information*) as a white solid (25 mg, 7%).

Analytical UV-LCMS-HPLC R_t = 6.67 min (5–95% B over 15 min),
Purity: 94%, determined by analytical UV-LCMS-HPLC at 220 nm
LRMS compound molecular formula (M): $C_{147}H_{246}N_{46}O_{53}$
m/z calcd. for $[M+4H]^{4+}$: monoisotopic 876.9565, predicted most abundant 877.2033, ***m/z* found for $[M+4H]^{4+}$: 887.1**

1.4.9. Synthesis of DualAgo 8

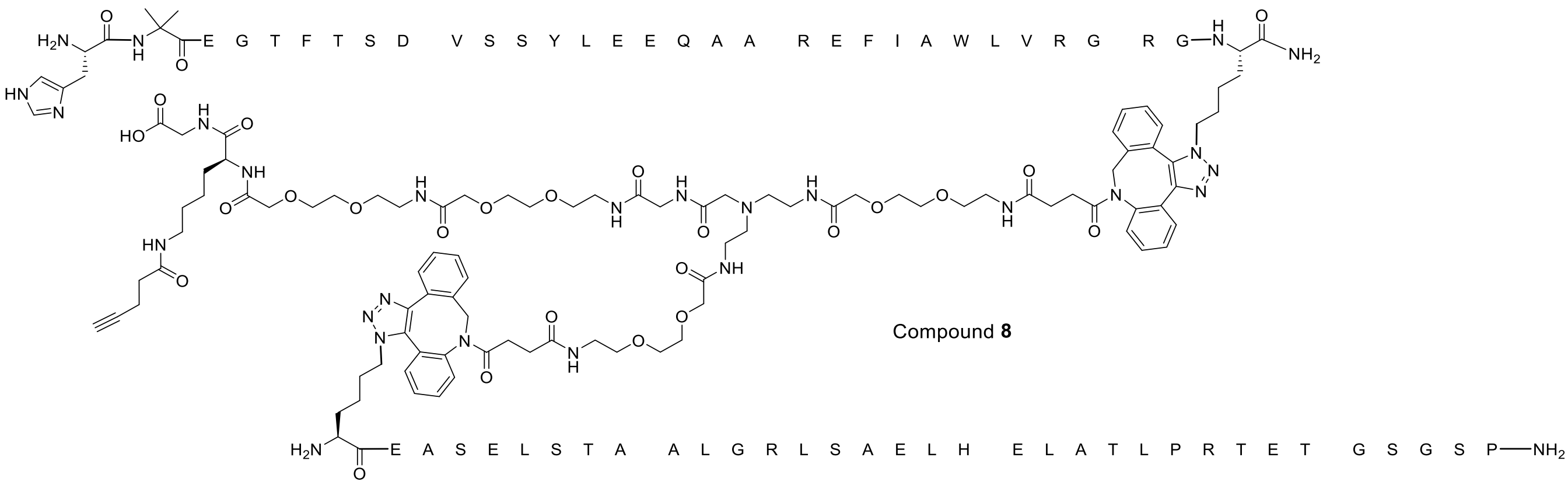

Peptide **6** (8.83 mg, 1.67 µmol) and amylinR agonist **7** (7.03 mg, 2.00 µmol, 1.2 eq.) were placed in a vial and dissolved in anhydrous DMSO (200 µL). The mixture was stirred at room temperature under an inert atmosphere for 16 hours. The crude product was purified by preparative UV-LCMS-HPLC (gradient 25–55% solvent B) and lyophilised to afford peptide **8** (8.45 mg, 57%) as a white solid, obtained as a mixture of regioisomers (Figure S1).[4]

Analytical HPLC R_t = 8.96 min (5–95% B over 15 min),
Purity: >99%, determined by analytical HPLC at 220 nm
LRMS compound molecular formula (M): $C_{391}H_{598}N_{108}O_{124}$
m/z calcd. for $[M+8H]^{8+}$: monoisotopic 1099.8049, predicted most abundant 1100.1811, ***m/z* found for $[M+8H]^{8+}$: 1100.3**
m/z calcd. for $[M+H]^{+}$: monoisotopic 8791.3881, predicted most abundant 8794.3982, ***m/z* deconvoluted for $[M+H]^{+}$: 8795**

1.4.10. Synthesis of lipidic chain S9

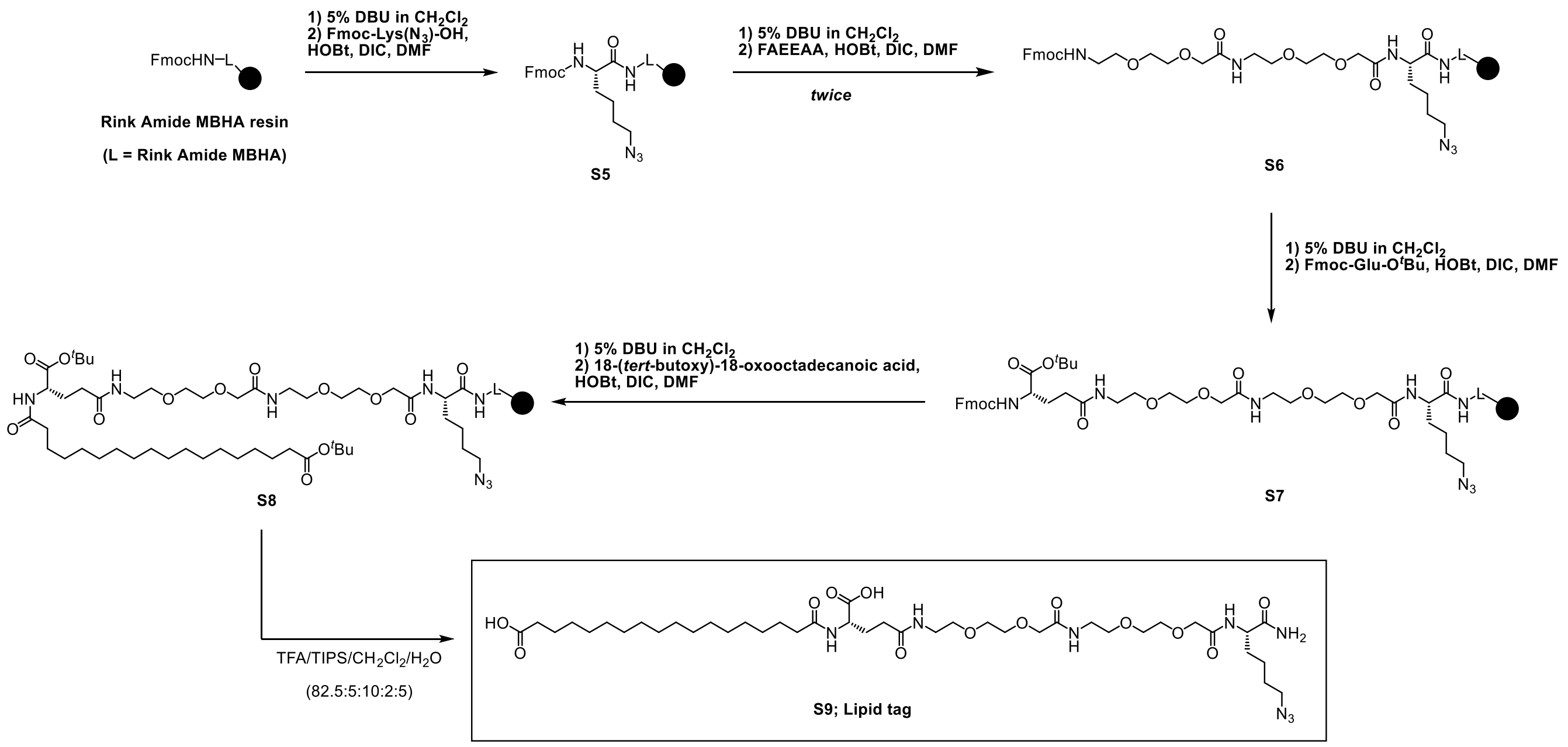

Table S2. Reagents table for SPS of lipidic chain S9

Cmpd.	Carboxylic acid used	CAS (carboxylic acid)	MW	Conc. [M]	n [mmol]	m [mg]	Calcd. *m/z* $[M+H]^+$
**S5**	Fmoc-Lys(N_3)-OH	159610-89-6	394	0.2	0.5	197	395
**S6**	{2-[2-(Fmoc-amino)ethoxy]ethoxy}acetic acid (FAEEAA)	166108-71-0	385	0.2	0.5	193	684
**S7**	Fmoc-Glu-O^{t}Bu	84793-07-7	425	0.2	0.5	212	813
**S8**	18-(tert-butoxy)-18-oxooctadecanoic acid	843666-40-0	370	0.2	0.5	185	888

Resin scale 250 mg (Rink Amide MBHA resin, loading 0.36 mmol/g). Compounds have been prepared according to the *1.3.1. General Procedure **A** (amide coupling)* and *1.3.2. General Procedure **B** (manual Fmoc deprotection).* After deprotection steps, the resin was washed with CH_2Cl_2 (3 × 2.5 mL) and DMF (3 × 2.5 mL). Amide couplings were performed using HOBt.H_2O (0.2 M; 0.5 mmol, 77 mg), DIC (0.2 M; 0.5 mmol, 77 µL), and the respective acid (0.2 M, 0.5 mmol), dissolved in DMF (2.5 mL) for 4 hours or in the case of the last coupling step 16 hours. After coupling steps, the resin was washed with DMF (3 × 2.5 mL) and CH_2Cl_2 (3 × 2.5 mL). The final product was cleaved off the resin in a cleavage cocktail TFA/TIPS/CH_2Cl_2/H_2O (82.5:5:10:2.5, 20 mL) for 2 h, precipitated in (cold) diethyl ether, and centrifuged. Upon centrifugation, the residual solvent was decanted, and the crude product was purified by preparative UV-LCMS-HPLC (gradient 45-75% B) to yield **S9** as a white solid (58 mg, 73%).

Analytical HPLC R_t = 11.51 min (5–95% B over 15 min)
Purity: 97%, determined by analytical HPLC at 220 nm
LRMS compound molecular formula (M): $C_{41}H_{74}N_8O_{13}$
m/z calcd. for $[M+H]^+$: monoisotopic 887.5448, ***m/z* found for $[M+H]^+$: 887.6**

▪ ***Crude UV-LCMS traces of linker intermediates (upon cleavage from the resin)***

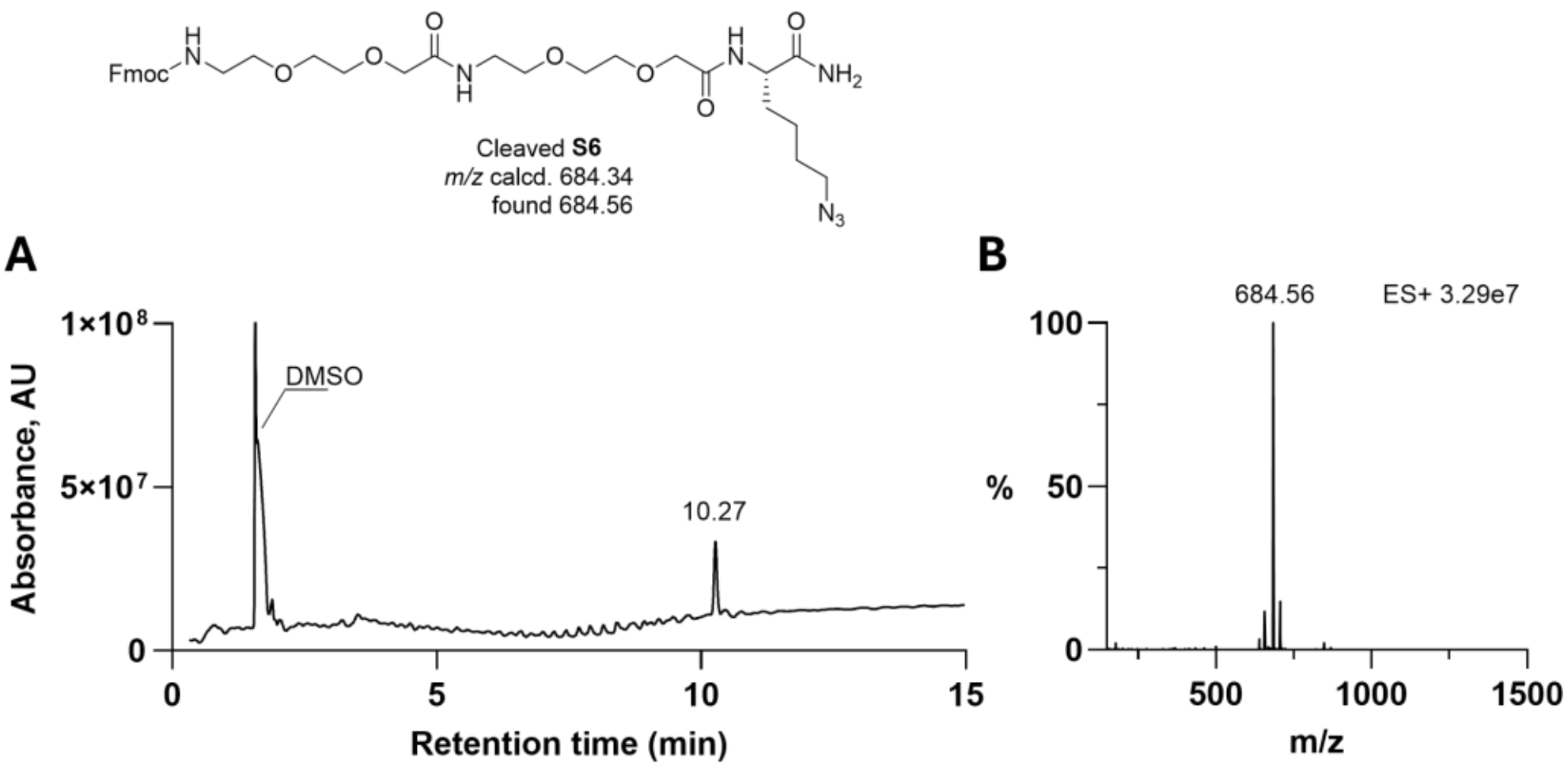

Figure S4. A. Crude analytical UV-LCMS-HPLC (5–95%B) trace. **B.** Crude LRMS ion abundance at R_t = 10.27 min.

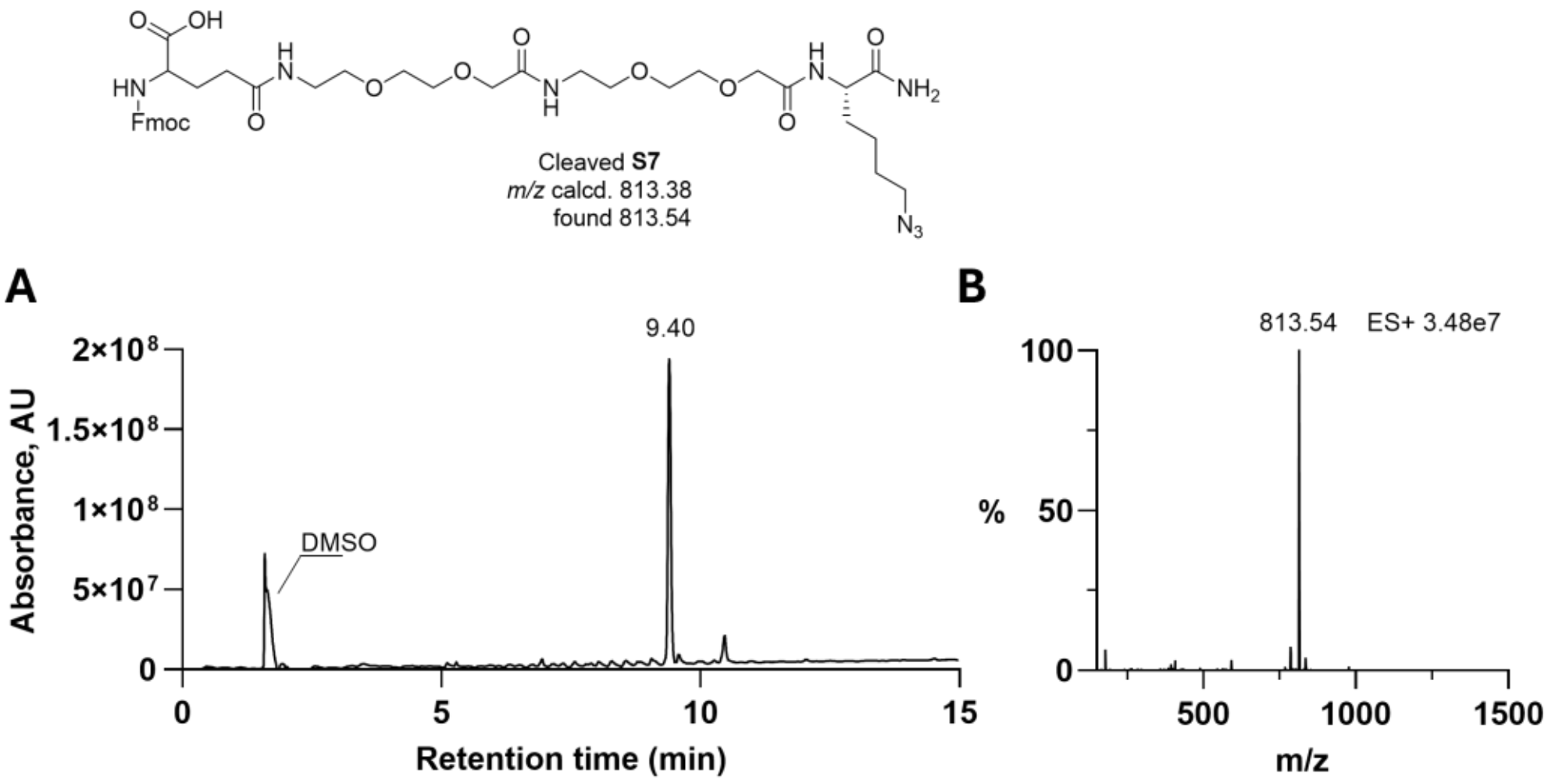

Figure S5. A. Crude analytical UV-LCMS-HPLC (5–95%B) trace. **B.** Crude LRMS ion abundance at R_t = 9.40 min.

1.4.11. Synthesis of final compound 9

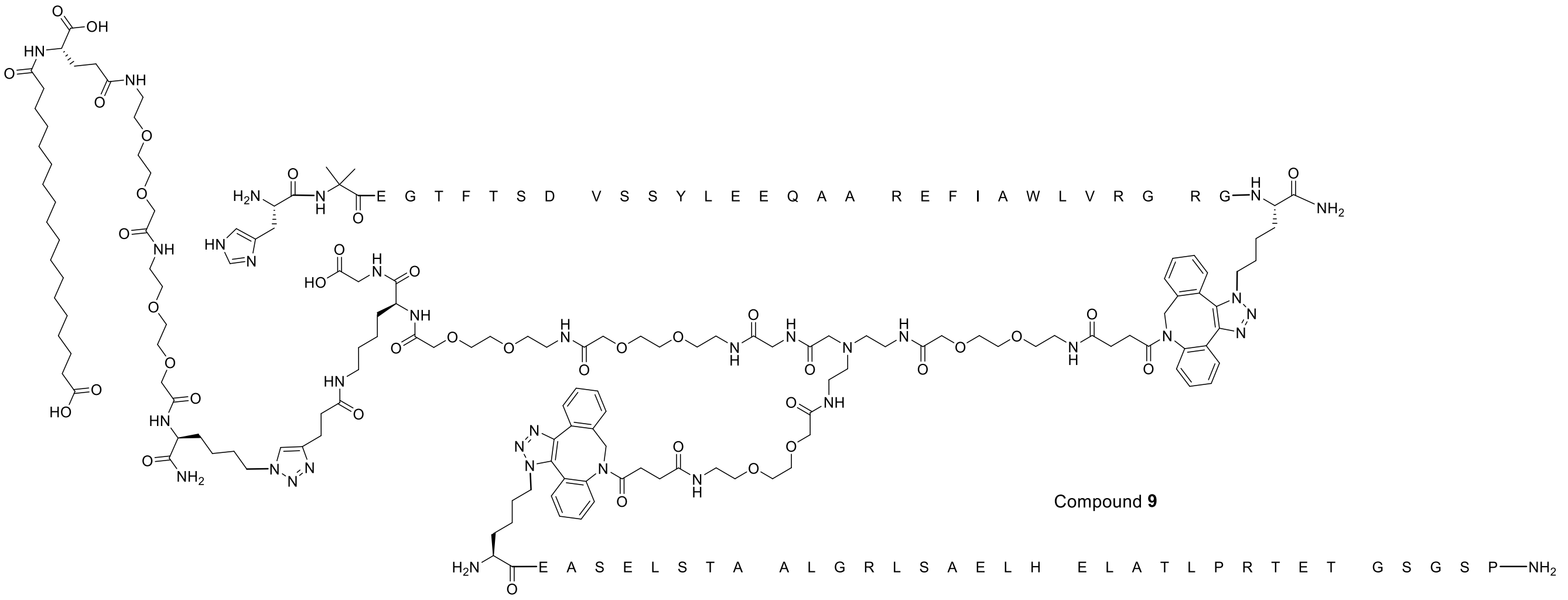

Compound **9** was synthesised according to the *1.4.3. General procedure **C** (CuAAC reaction)* starting with compound **8** (1.15 mg, 0.13 µmol) and a lipid tag **S9** (0.17 mg, 0.20 µmol). The product was purified by preparative UV-LCMS-HPLC (gradient 5-95% B) and lyophilised to yield final compound **9** (0.6 mg, 48%) as a white solid, mixture of regioisomers (Figure S1).[4]

Analytical HPLC R_t = 9.28 min (5–95% B over 15 min)

Purity: 98%, determined by analytical HPLC at 220 nm

LRMS compound molecular formula (M): $C_{432}H_{672}N_{116}O_{137}$

m/z calcd. for $[M+8H]^{8+}$: monoisotopic 1210.5221, predicted most abundant 1210.9983, ***m/z* found for $[M+8H]^{8+}$: 1211.2**

m/z calcd. for $[M+H]^{+}$: monoisotopic 9677.9256, predicted most abundant 9680.9357, ***m/z* deconvoluted for $[M+H]^{+}$: 9682**

1.4.12. Synthesis of final compound 10

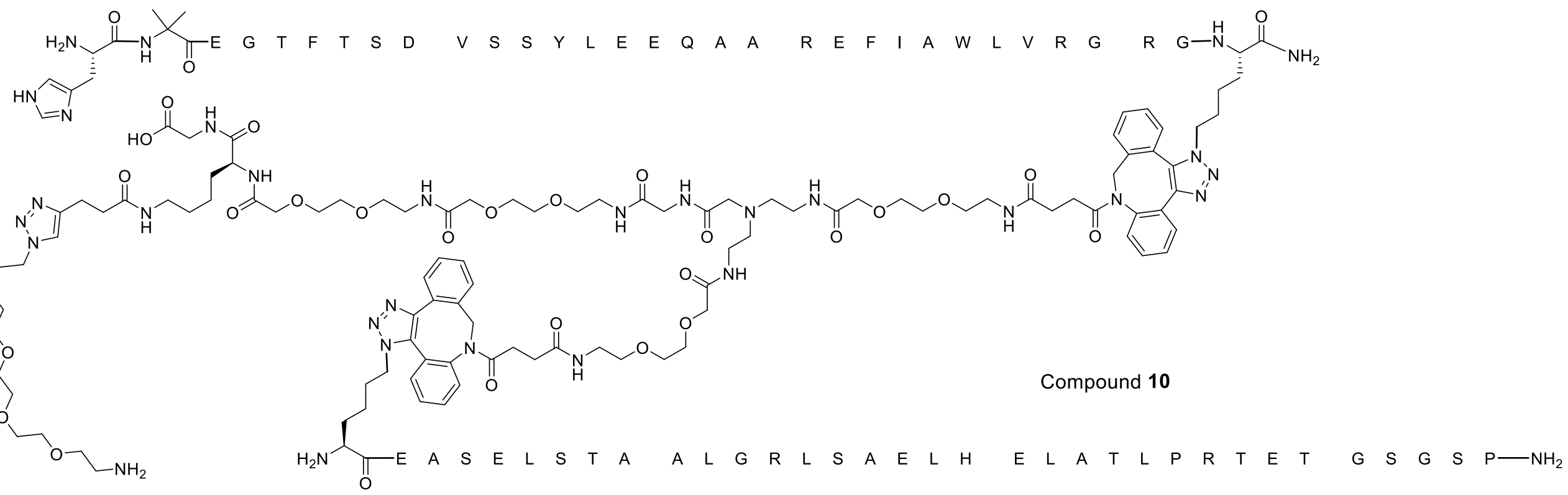

Compound **10** was synthesised according to the *1.4.3. General procedure **C** (CuAAC reaction)* starting with compound **8** (2.11 mg, 0.24 µmol) and commercially available 14-azido-3,6,9,12-tetraoxatetradecan-1-amine (CAS: 951671-92-4; 0.094 mg, 0.36 µmol). The crude product was purified by preparative UV-LCMS-HPLC (gradient 5-95% B) and lyophilised to yield final compound **10** (0.91 mg, 42%) as a white solid, mixture of regioisomers (Figure S1).[4]

Analytical HPLC R_t = 9.19 min (5–95% B over 15 min)

Purity: 96%, determined by analytical HPLC at 220 nm

LRMS compound molecular formula (M): $C_{401}H_{620}N_{112}O_{128}$

m/z calcd. for $[M+9H]^{9+}$: monoisotopic 1006.8456, predicted most abundant 1007.1801, ***m/z* found for $[M+9H]^{9+}$: 1007.4**

m/z calcd. for $[M+H]^{+}$: monoisotopic 9053.5522, predicted most abundant 9056.5623, ***m/z* deconvoluted for $[M+H]^{+}$: 9058**

1.4.13. Synthesis of final compound 11

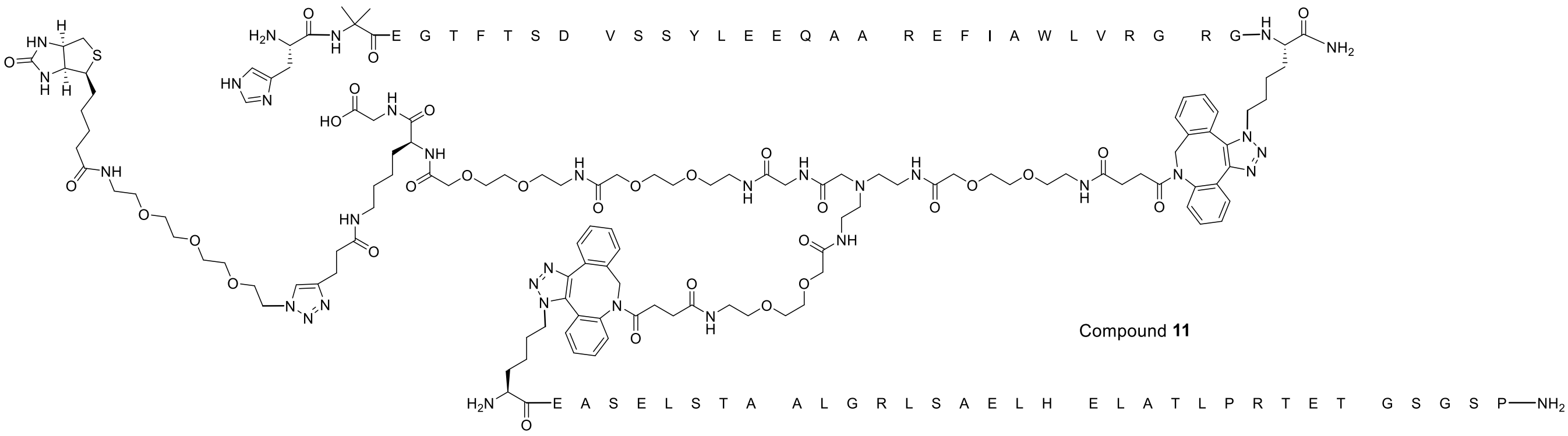

Compound **11**

Compound **11** was synthesised according to the *1.4.3. General procedure **C** (CuAAC reaction)* starting with compound **8** (2.20 mg, 0.23 µmol) and commercially available biotin-PEG$_3$-azide (CAS: 875770-34-6; 0.133 mg, 0.30 µmol). The crude product was purified by preparative UV-LCMS-HPLC (gradient 5-95% B) and lyophilised to yield final compound **11** (0.79 mg, 37%) as a white solid, mixture of regioisomers (Figure S1).[4]

Analytical HPLC R_t = 8.77 min (5–95% B over 15 min)

Purity: 95%, determined by analytical HPLC at 220 nm

LRMS compound molecular formula (M): $C_{409}H_{630}N_{114}O_{129}S$

m/z calcd. for $[M+8H]^{8+}$: monoisotopic 1155.2910, predicted most abundant 1155.7081, ***m/z* found for $[M+8H]^{8+}$: 1156.0**

m/z calcd. for $[M+H]^{+}$: monoisotopic 9235.6035, predicted most abundant 9238.6137, ***m/z* deconvoluted for $[M+H]^{+}$: 9239**

1.4.14. Synthesis of final compound 12

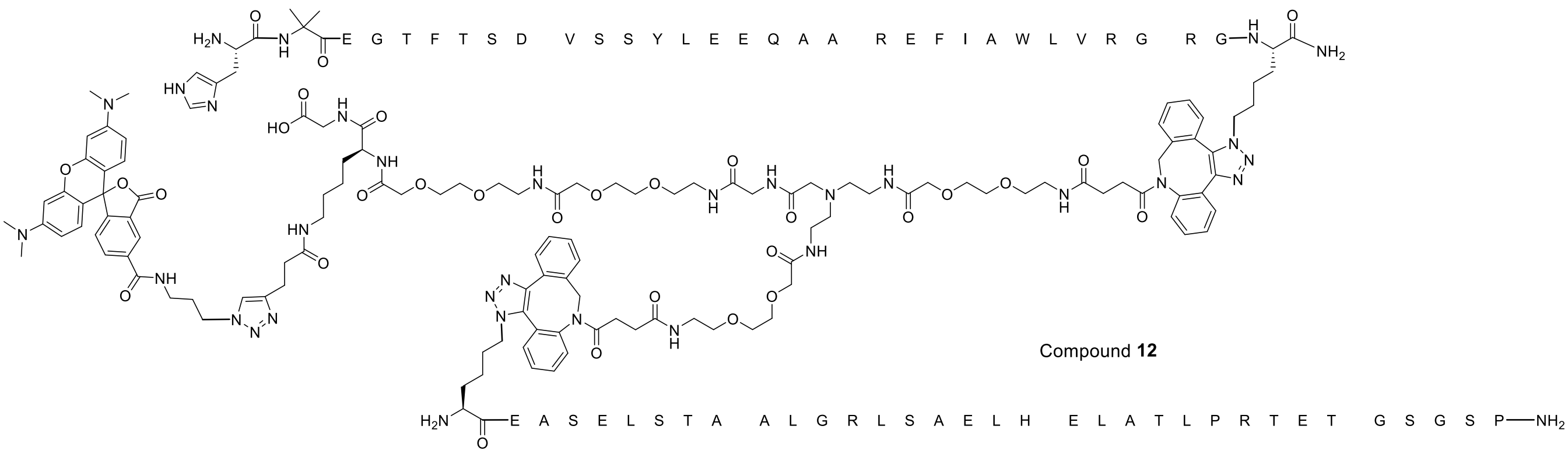

Compound **12**

Compound **12** was synthesised according to the *1.4.3. General procedure **C** (CuAAC reaction)* starting with compound **8** (2.00 mg, 0.25 µmol) and TAMRA azide, 5-isomer (CAS: 825651-66-9; 0.13 mg, 0.25

µmol, 1 eq). The crude product was purified by preparative UV-LCMS-HPLC (gradient 25-55% B) and lyophilised to yield final compound **12** (0.52 mg, 22%) as a magenta solid, mixture of regioisomers (Figure S1).[4]

Analytical HPLC R_t = 8.82 min (5–95% B over 15 min)
Purity: 90%, determined by analytical HPLC at 220 nm
LRMS compound molecular formula (M): $C_{419}H_{626}N_{114}O_{128}$
m/z calcd. for $[M+8H]^{8+}$: monoisotopic 1163.8320, predicted most abundant 1164.2083, ***m/z* found for $[M+8H]^{8+}$: 1164.3**
m/z calcd. for $[M+H]^{+}$: monoisotopic 9303.6053, predicted most abundant 9306.6154, ***m/z* deconvoluted for $[M+H]^{+}$: 9308**

1.4.15. Synthesis of DOTA tag S11

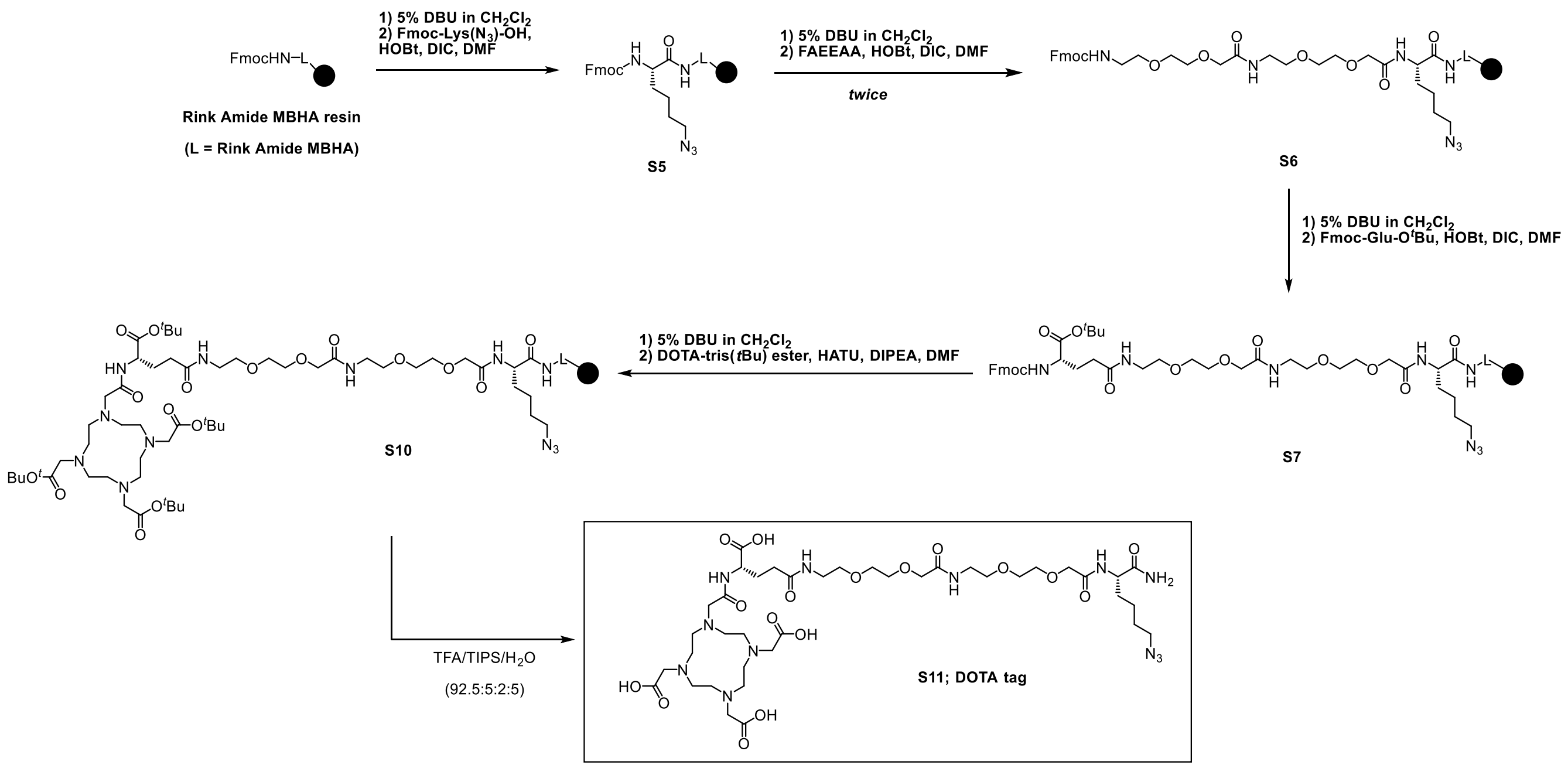

Table S3. Reagents table for SPS of DOTA tag S11

Cmpd.	Carboxylic acid used	CAS (carboxylic acid)	MW	Conc. [M]	n [mmol]	m [mg]	Calcd. *m/z* $[M+H]^+$
**S5**	Fmoc-Lys(N_3)-OH	159610-89-6	394	0.2	0.3	135	394
**S6**	{2-[2-(Fmoc-amino)ethoxy]ethoxy}acetic acid (FAEEAA)	166108-71-0	385	0.2	0.3	116	684
**S7**	Fmoc-Glu-O^{t}Bu	84793-07-7	425	0.2	0.3	89	813
**S10**	DOTA-tris(^{t}Bu) ester	137076-54-1	572	0.15	0.2	129	979

Resin scale 150 mg (Rink Amide MBHA resin, loading 0.36 mmol/g). Compounds have been prepared according to the *1.3.1. General Procedure **A** (amide coupling)* and *1.3.2. General Procedure **B** (manual Fmoc deprotection).* Notably, for amide coupling of DOTA-tris (^{t}Bu) ester, HATU/DIPEA coupling reagents have been used instead of HOBt/DIC. After deprotection steps, the resin was washed with CH_2Cl_2 (3 × 2.5 mL) and DMF (3 × 2.5 mL). Amide couplings were performed using HOBt.H_2O (0.2 M;

0.3 mmol, 46 mg), DIC (0.2 M; 0.3 mmol, 46 µL), and the respective acid (0.2 M, 0.3 mmol), dissolved in DMF (1.5 mL) for 4 hours. The final coupling was conducted with HATU (0.15 M, 0.2 mmol, 86 mg), DIPEA (0.3 M, 0.45 mmol, 78 µL) and 0.15 M DOTA-tris(^{t}Bu) ester (137076-54-1, 0.15 M, 0.2 mmol, 129 mg) for 2 hours. After coupling steps, the resin was washed with DMF (3 × 2.5 mL) and CH_2Cl_2 (3 × 2.5 mL). The final product was cleaved off the resin in a cleavage cocktail TFA/TIPS/H_2O (92.5:5:2.5, 10 mL) for 4 hours, precipitated in (cold) diethyl ether and centrifuged. Upon centrifugation, the residual solvent was decanted, and the crude product was purified by preparative UV-LCMS-HPLC (gradient 10-60% B) to yield **S11** as a white solid (37 mg, 70%).

Analytical HPLC R_t = 5.50 min (5–95% B over 15 min)
Purity: 95%, determined by analytical HPLC at 220 nm
LRMS compound molecular formula (M): $C_{39}H_{68}N_{12}O_{17}$
m/z calcd. for $[M+H]^+$: monoisotopic 977.4898, ***m/z* found for $[M+H]^+$: 977.45**

1.4.16. Synthesis of final compound 13

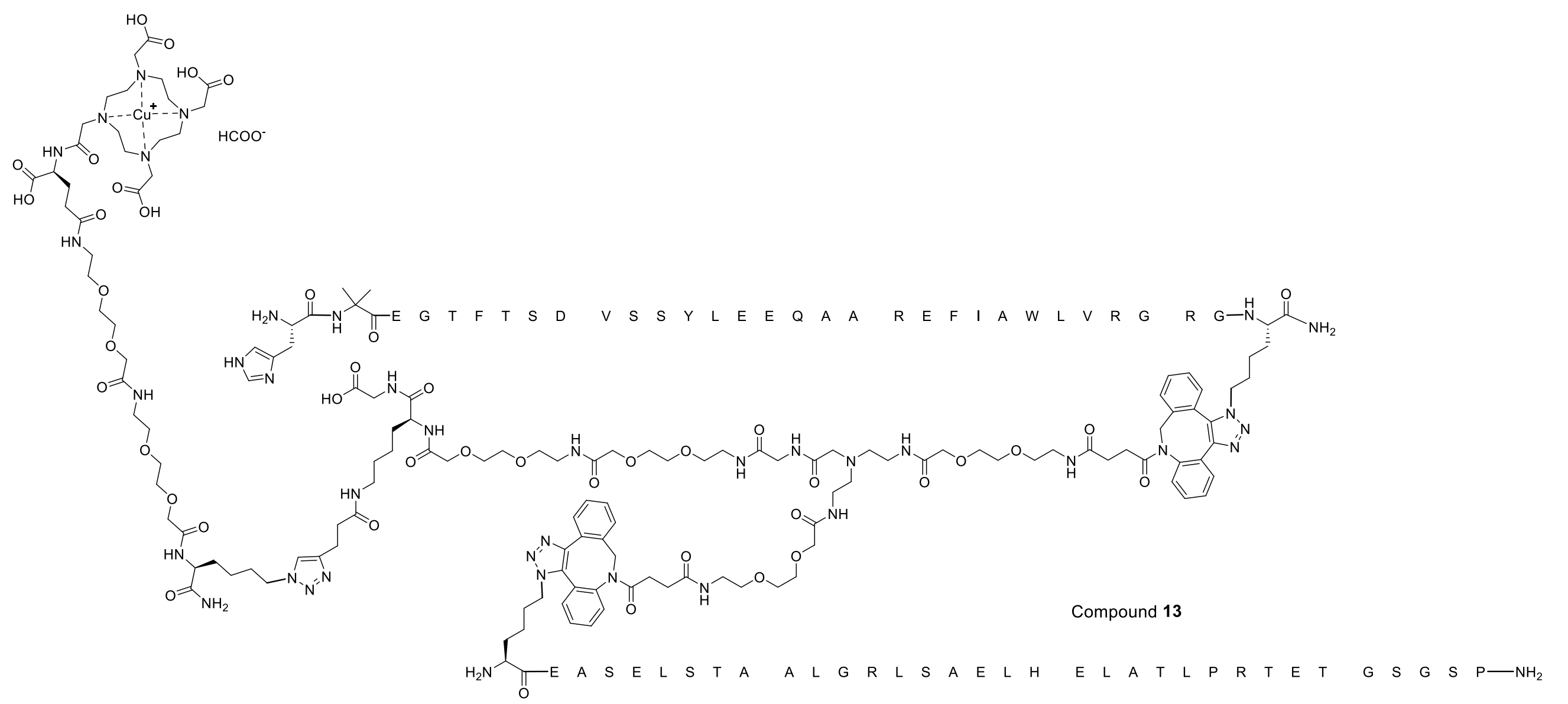

Compound **13**

Compound **13** was synthesised according to the *1.4.3. General procedure **C** (CuAAC reaction)* starting with compound **8** (2.00 mg, 0.23 µmol) and compound **S11** (0.29 mg, 0.30 µmol). The crude product was purified by preparative UV-LCMS-HPLC (gradient 5-95% B) and lyophilised to yield final compound **13** (0.90 mg, 40%) as an off-white solid, copper chelate formic acid salt, mixture of regioisomers (Figure S1).[4]

Analytical HPLC R_t = 8.67 min (5–95% B over 15 min)
Purity: 86%, determined by analytical HPLC at 220 nm
LRMS peptide ion molecular formula (M): $C_{430}H_{666}N_{120}O_{141}Cu^+$
m/z calcd. for $[M+7H]^{8+}$: monoisotopic 1229.6054, predicted most abundant 1229.9817, ***m/z* found for $[M+7H]^{8+}$: 1230.2**
m/z calcd. for $[M]^+$: monoisotopic 9829.7924, predicted most abundant 9832.8035, ***m/z* deconvoluted for $[M]^+$: 9834**

1.4.17. Synthesis of final compound 14

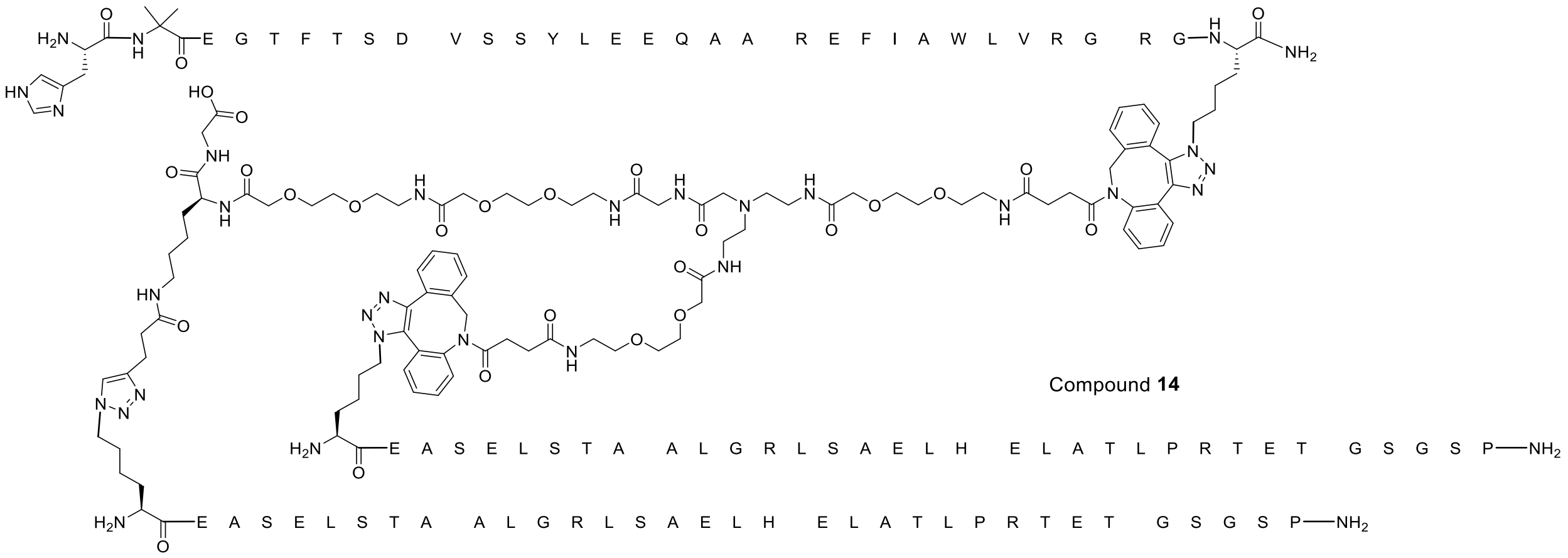

Compound **14**

Compound **14** was synthesised according to the *1.4.3. General procedure **C** (CuAAC reaction)* starting with compound **8** (1.26 mg, 0.14 µmol) and compound **7** (0.6 mg, 0.17 µmol, 1.2 eq.). The reaction mixture was stirred at room temperature for 10 hours upon which was the crude product purified by preparative UV-LCMS-HPLC (gradient 25-55% B) and lyophilised to yield peptide **14** (0.70 mg, 40%) as a white solid, mixture of regioisomers (Figure S1).[4]

Analytical HPLC R_t = 8.63 min (5–95% B over 15 min)

Purity: 82%, determined by analytical HPLC at 220 nm

LRMS compound molecular formula (M): $C_{538}H_{844}N_{154}O_{177}$

m/z calcd. for $[M+11H]^{11+}$: monoisotopic 1118.9282, predicted most abundant 1119.2018, ***m/z* found for $[M+11H]^{11+}$: 1119.2**

m/z calcd. for $[M+H]^{+}$: monoisotopic 12298.1369, predicted most abundant 12301.1470, ***m/z* deconvoluted for $[M+H]^{+}$: 12301**

2. Analytical HPLC chromatograms and LRMS analysis

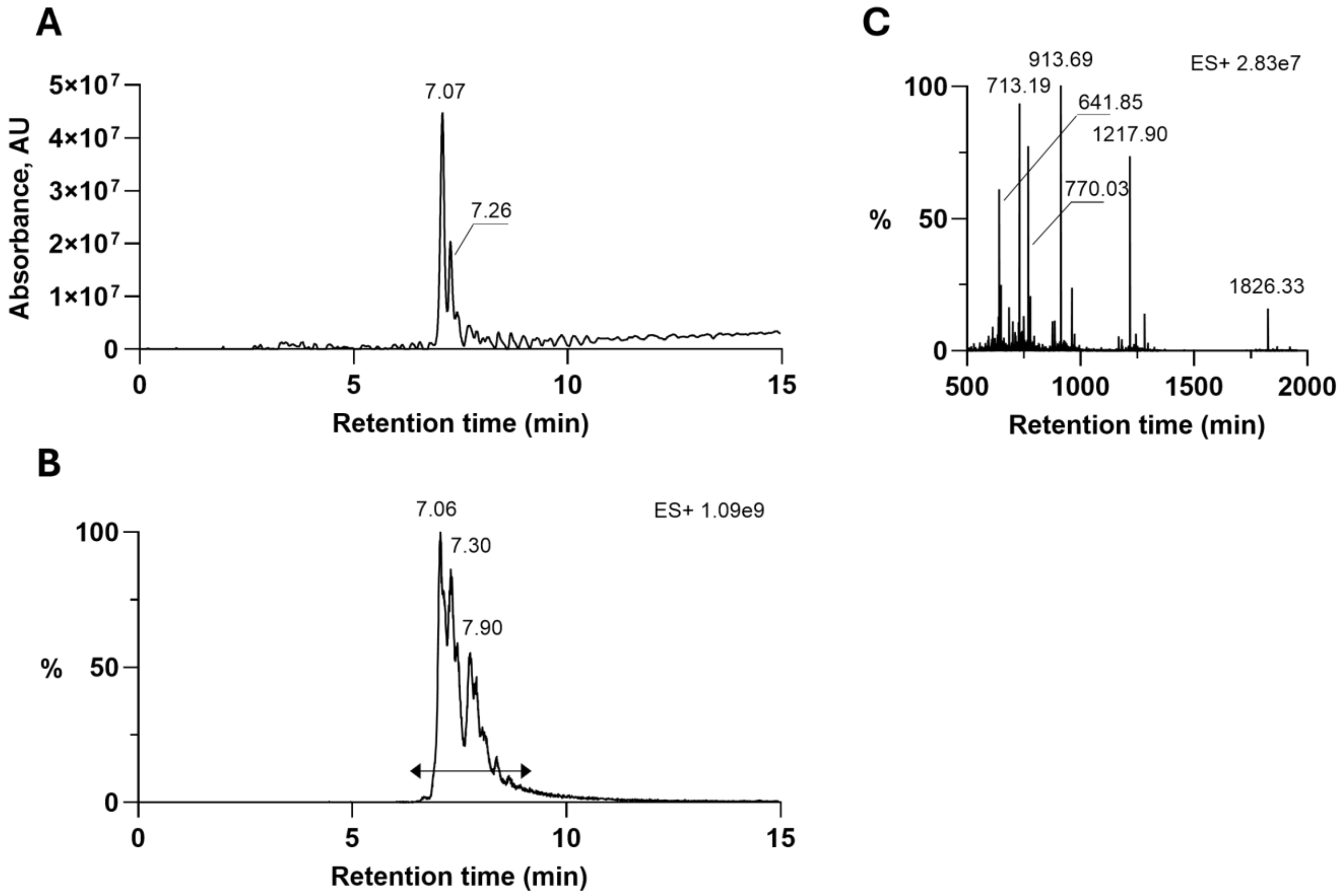

Figure S6. Characterisation of compound **5**. **A.** Analytical UV-LCMS-HPLC chromatogram R_t = 7.07 min (5–95% B over 15 min). **B.** LRMS total ion count trace. **C.** LRMS ion abundance at R_t = 7.06 min.

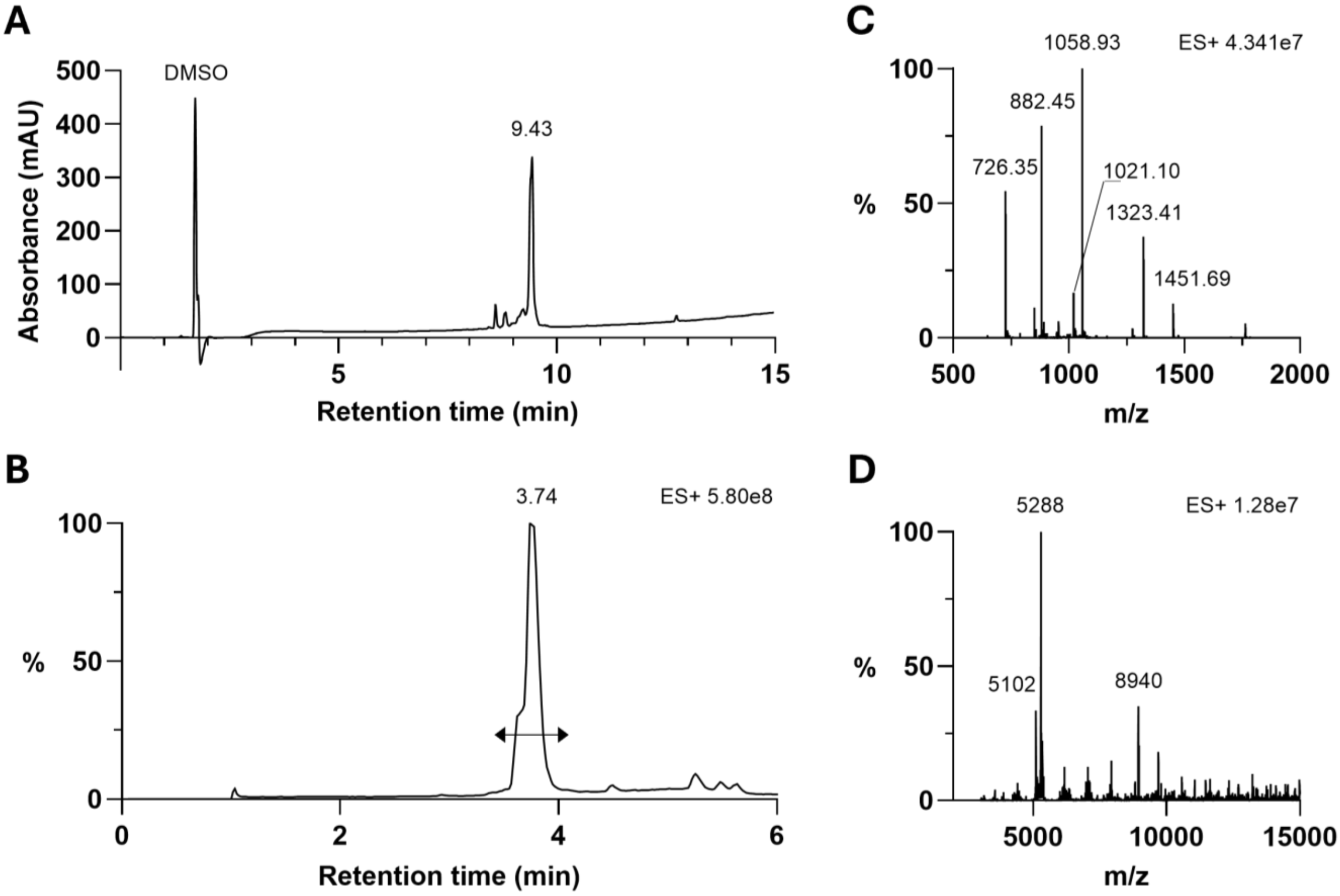

Figure S7. Characterisation of compound **6**. **A.** Analytical HPLC chromatogram R_t = 9.43 min (5–95% B over 15 min, 220 nm). **B.** LRMS total ion count trace (Peptide LCMS, Section 1.2). **C**. LRMS ion abundance at R_t = 3.74 min, **D.** LRMS deconvoluted trace.

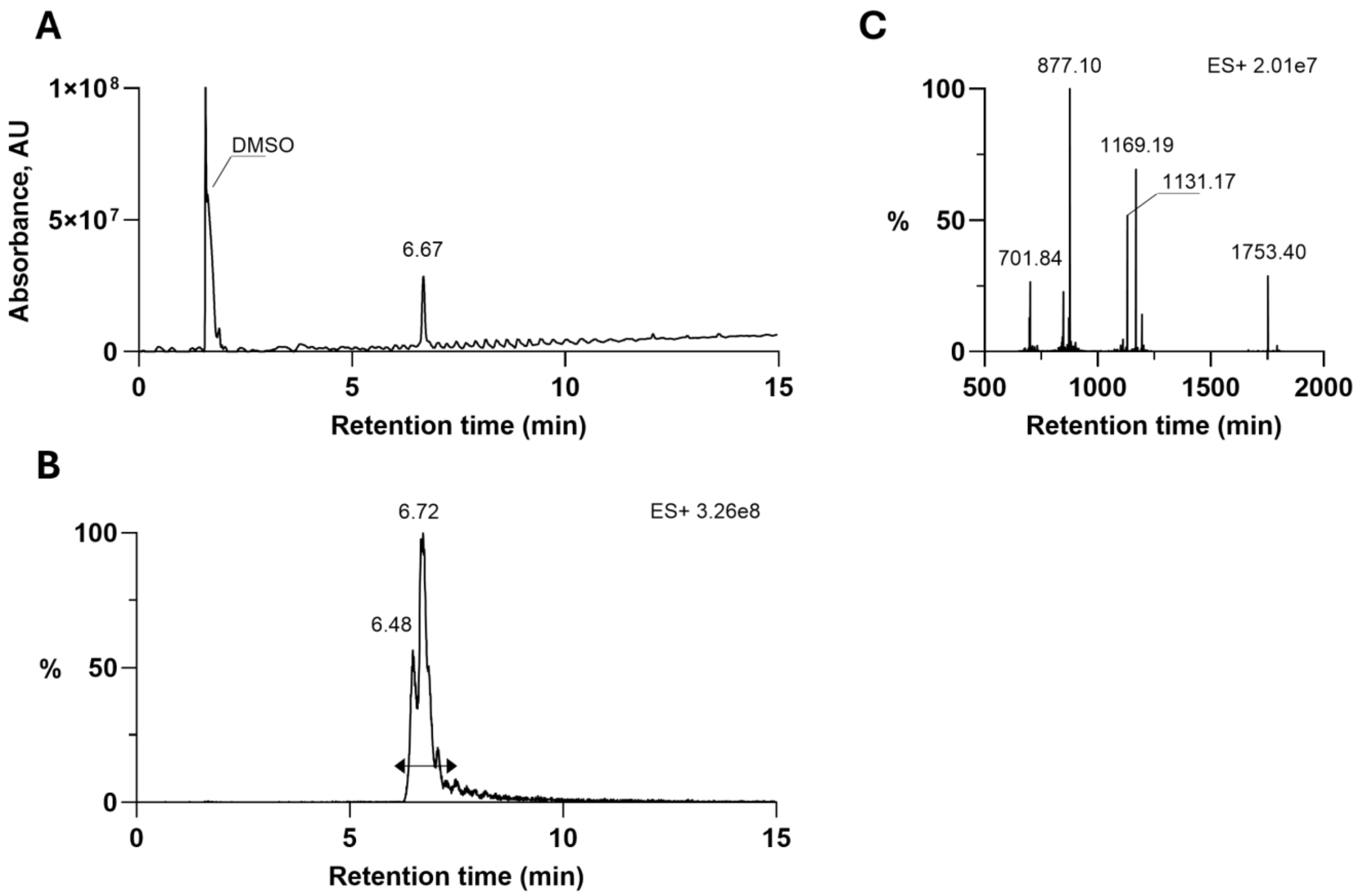

Figure S8. Characterisation of compound **7**. **A.** Analytical UV-LCMS-HPLC chromatogram R_t = 6.67 min (5–95% B over 15 min). **B.** LRMS total ion count trace. **C.** LRMS ion abundance at R_t = 6.67 min.

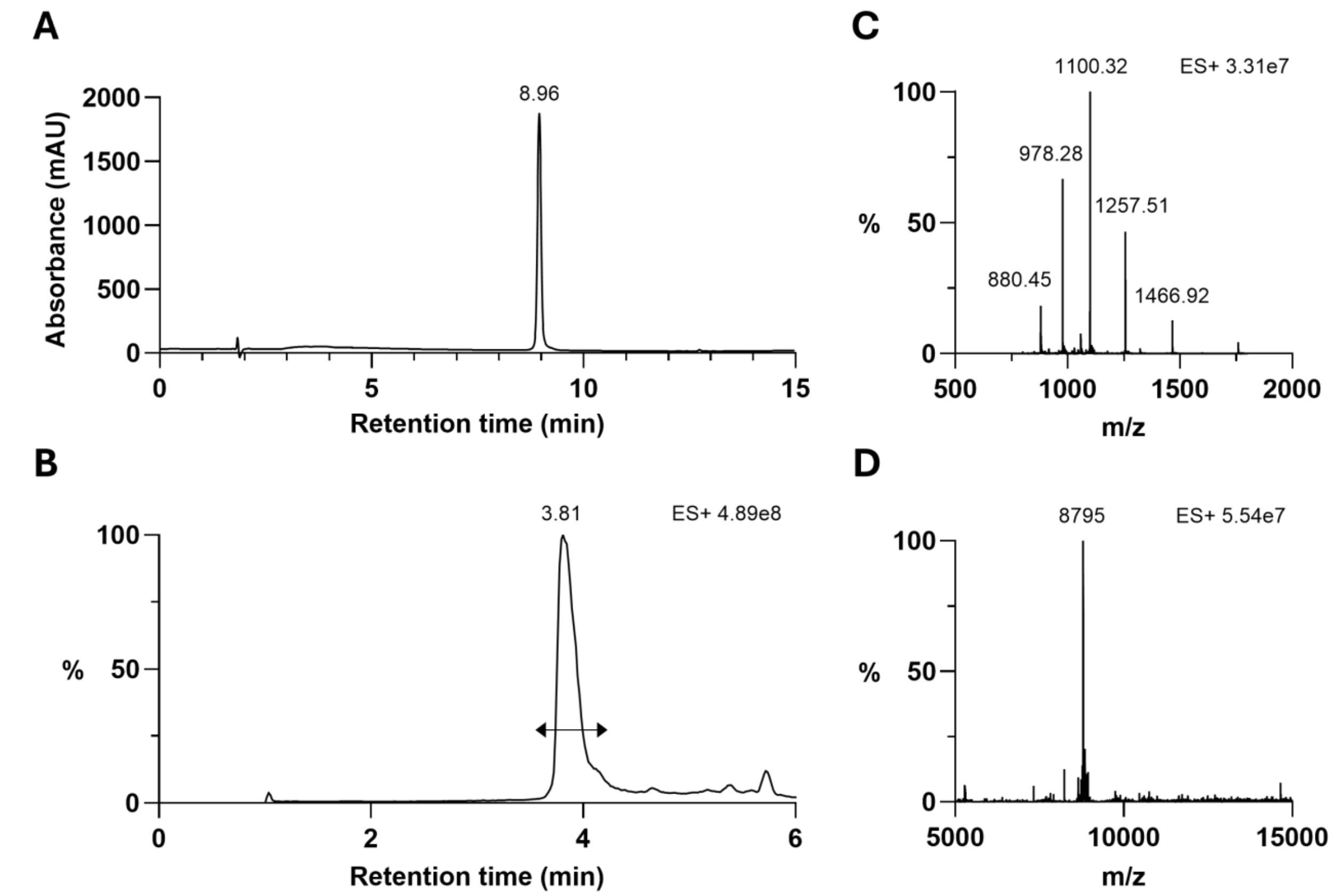

Figure S9. Characterisation of compound **8**. **A.** Analytical HPLC chromatogram R_t = 8.96 min (5–95% B over 15 min, 220 nm). **B.** LRMS total ion count trace (Peptide LCMS, Section 1.2). **C.** LRMS ion abundance at R_t = 3.81 min. **D.** LRMS deconvoluted trace.

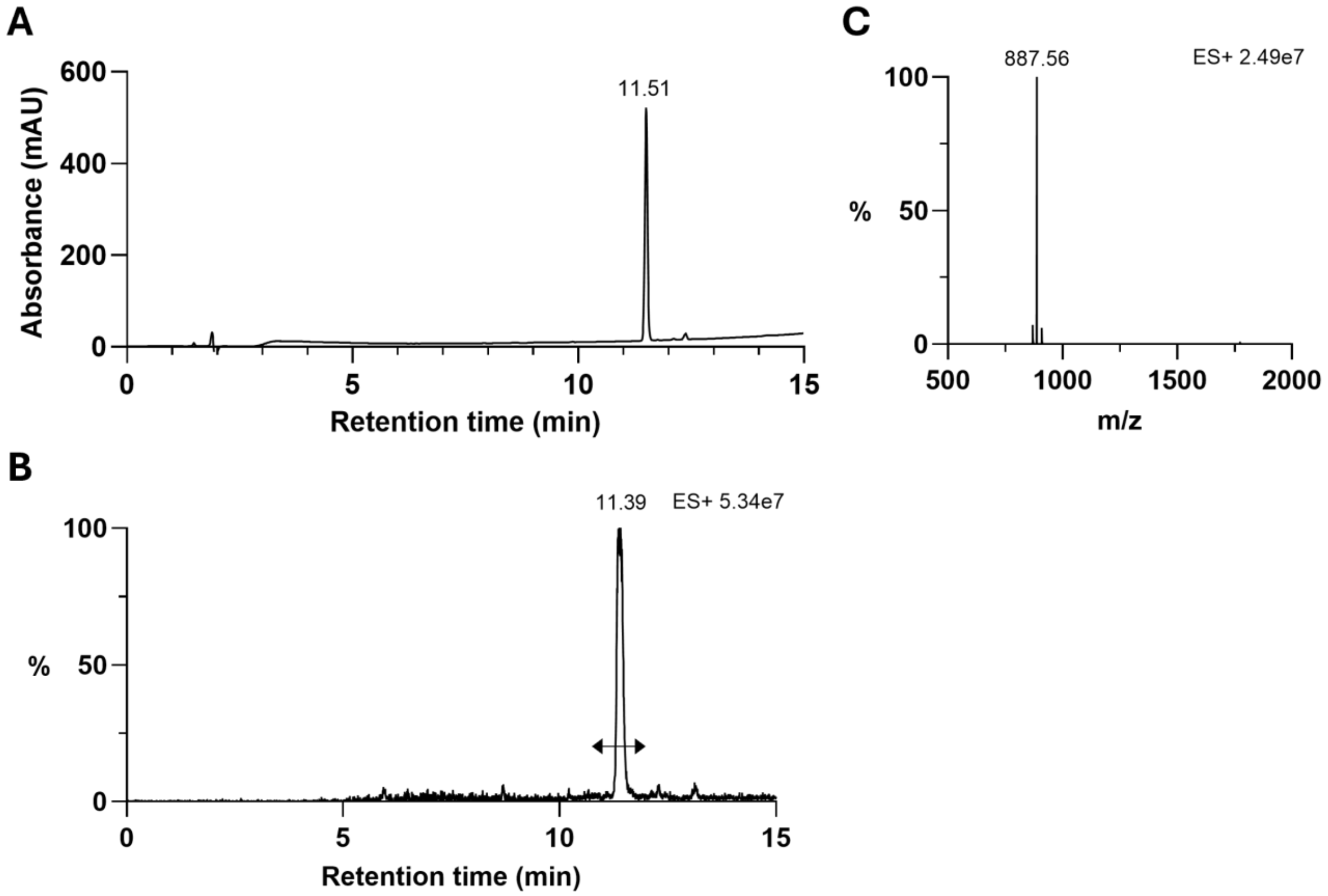

Figure S10. Characterisation of compound **S9**. **A.** Analytical UV-LCMS-HPLC chromatogram R_t = 11.51 min (5–95% B over 15 min, 220 nm). **B.** LRMS total ion count trace. **C.** LRMS ion abundance at R_t = 11.39 min.

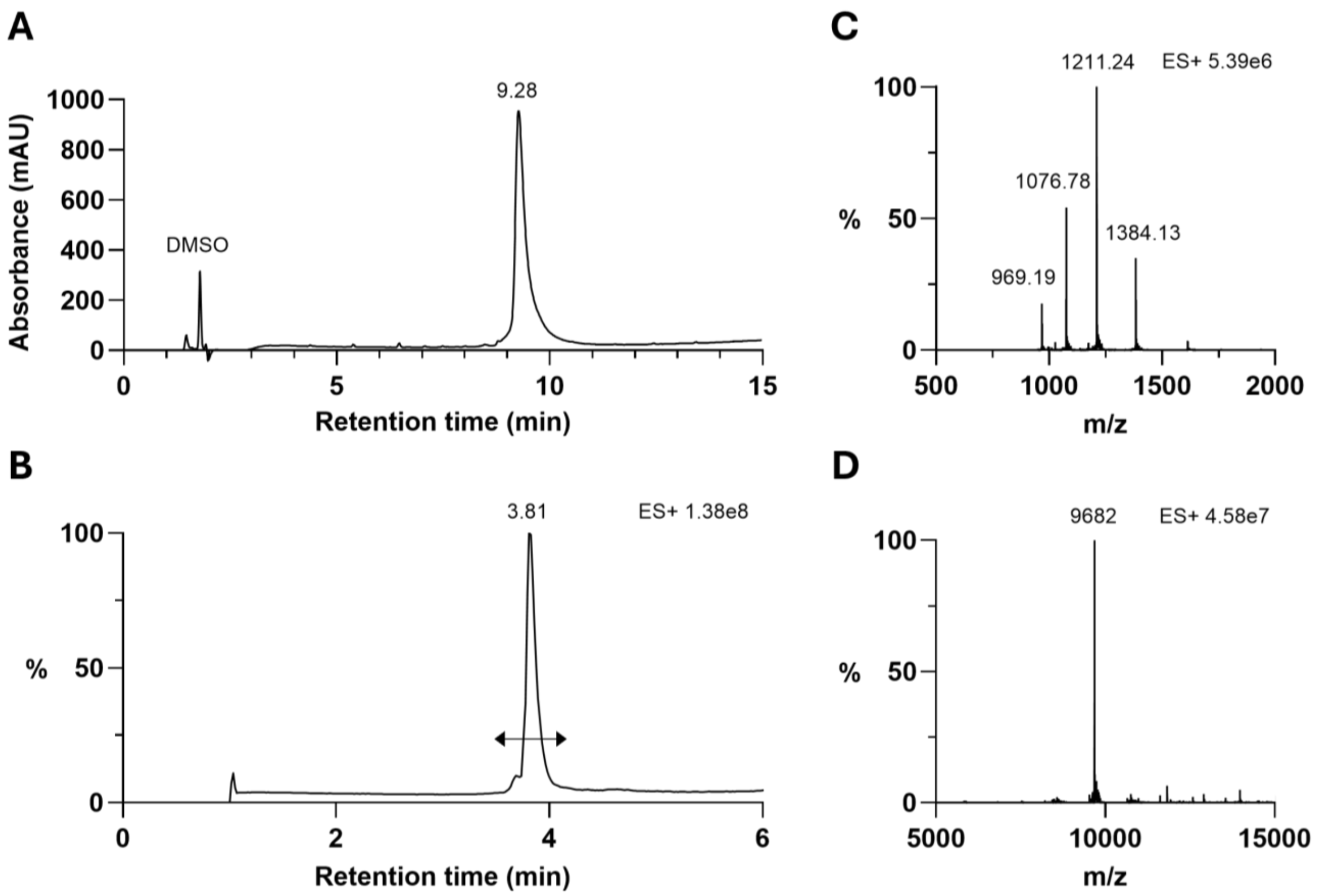

Figure S11. Characterisation of compound **9**. **A.** Analytical HPLC chromatogram R_t = 9.28 min (5–95% B over 15 min, 220 nm). **B.** LRMS total ion count trace (Peptide LCMS, Section 1.2). **C.** LRMS ion abundance at R_t = 3.81 min. **D.** LRMS deconvoluted trace.

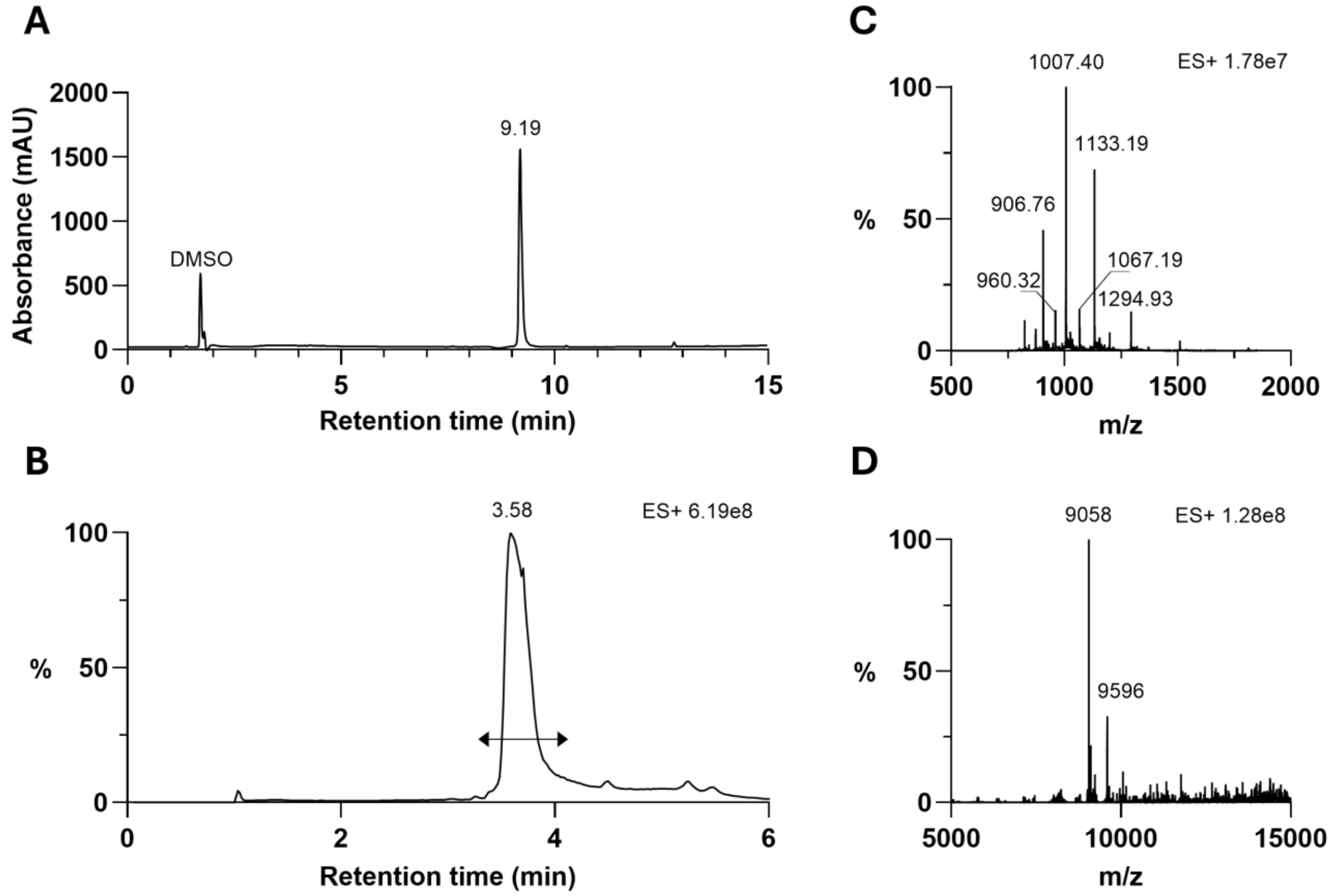

Figure S12. Characterisation of compound **10**. **A.** Analytical HPLC chromatogram R_t = 9.19 min (5–95% B over 15 min, 220 nm). **B.** LRMS total ion count trace (Peptide LCMS, Section 1.2). **C.** LRMS ion abundance at R_t = 3.58 min. **D.** LRMS deconvoluted trace.

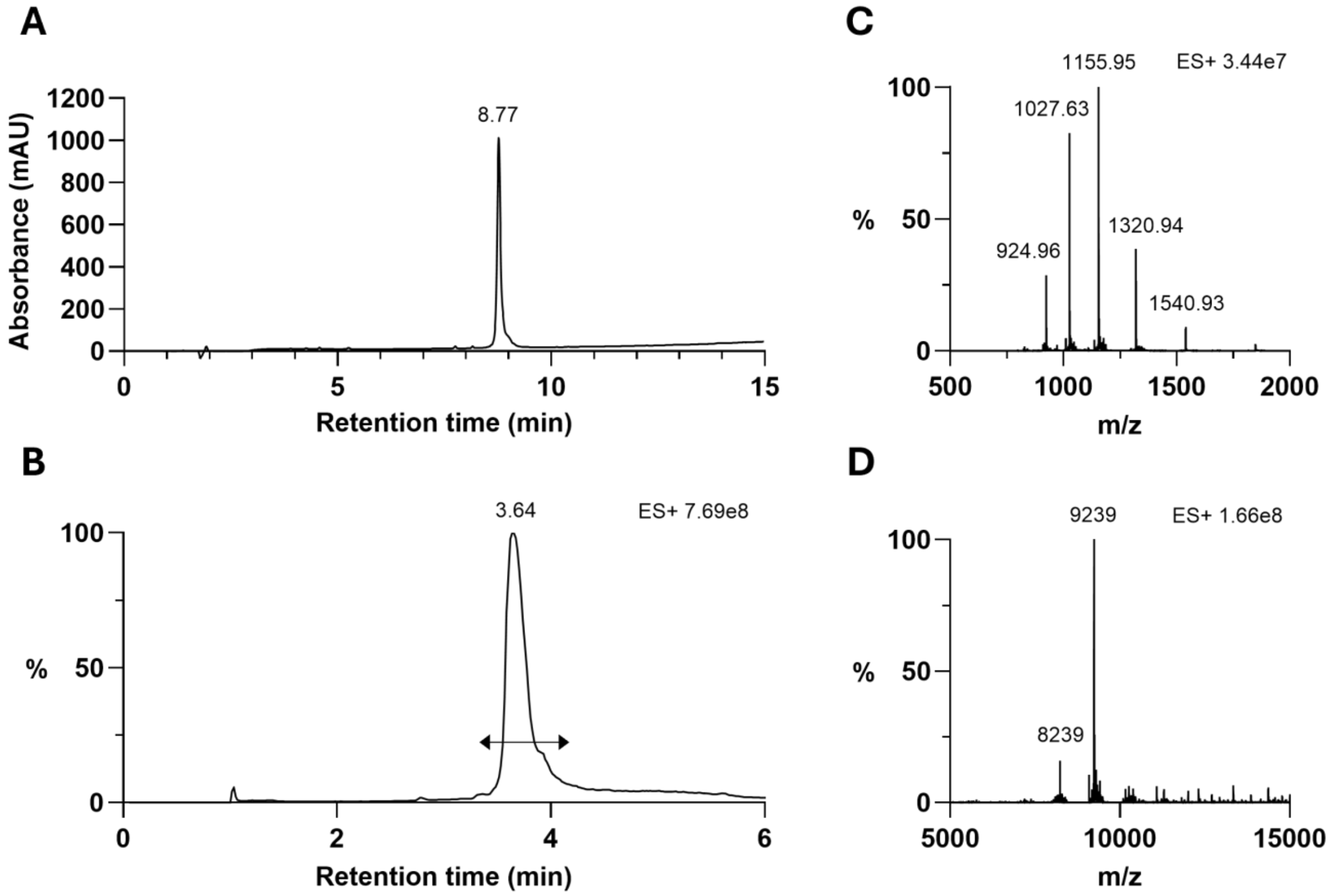

Figure S13. Characterisation of compound **11**. **A.** Analytical HPLC chromatogram R_t = 8.77 min (5–95% B over 15 min, 220 nm). **B.** LRMS total ion count trace (Peptide LCMS, Section 1.2). **C.** LRMS ion abundance at R_t = 3.81 min. **D.** LRMS deconvoluted trace.

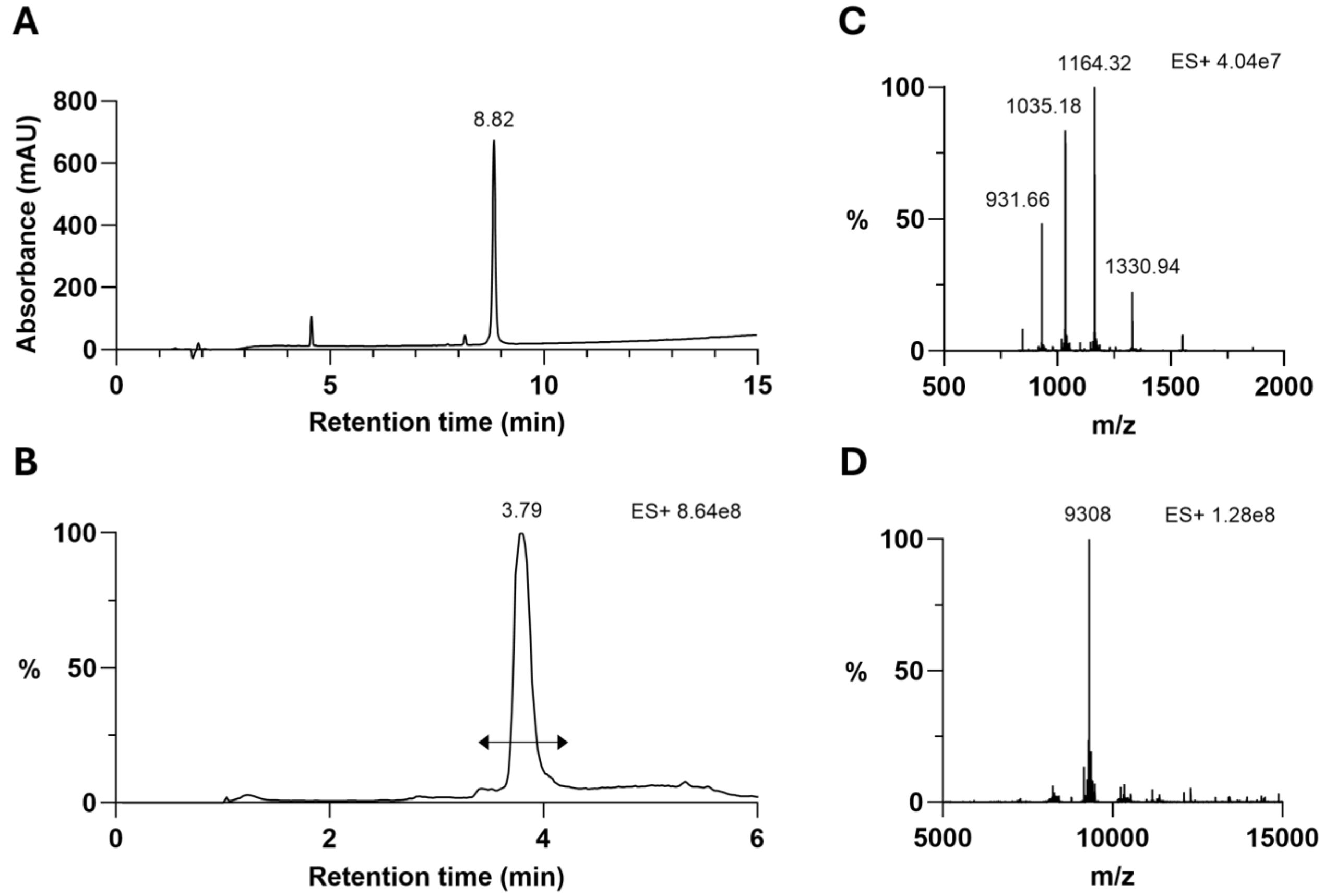

Figure S14. Characterisation of compound **12**. **A.** Analytical HPLC chromatogram R_t = 8.82 min (5–95% B over 15 min, 220 nm). **B.** LRMS total ion count trace (Peptide LCMS, Section 1.2). **C.** LRMS ion abundance at R_t = 3.79 min. **D.** LRMS deconvoluted trace.

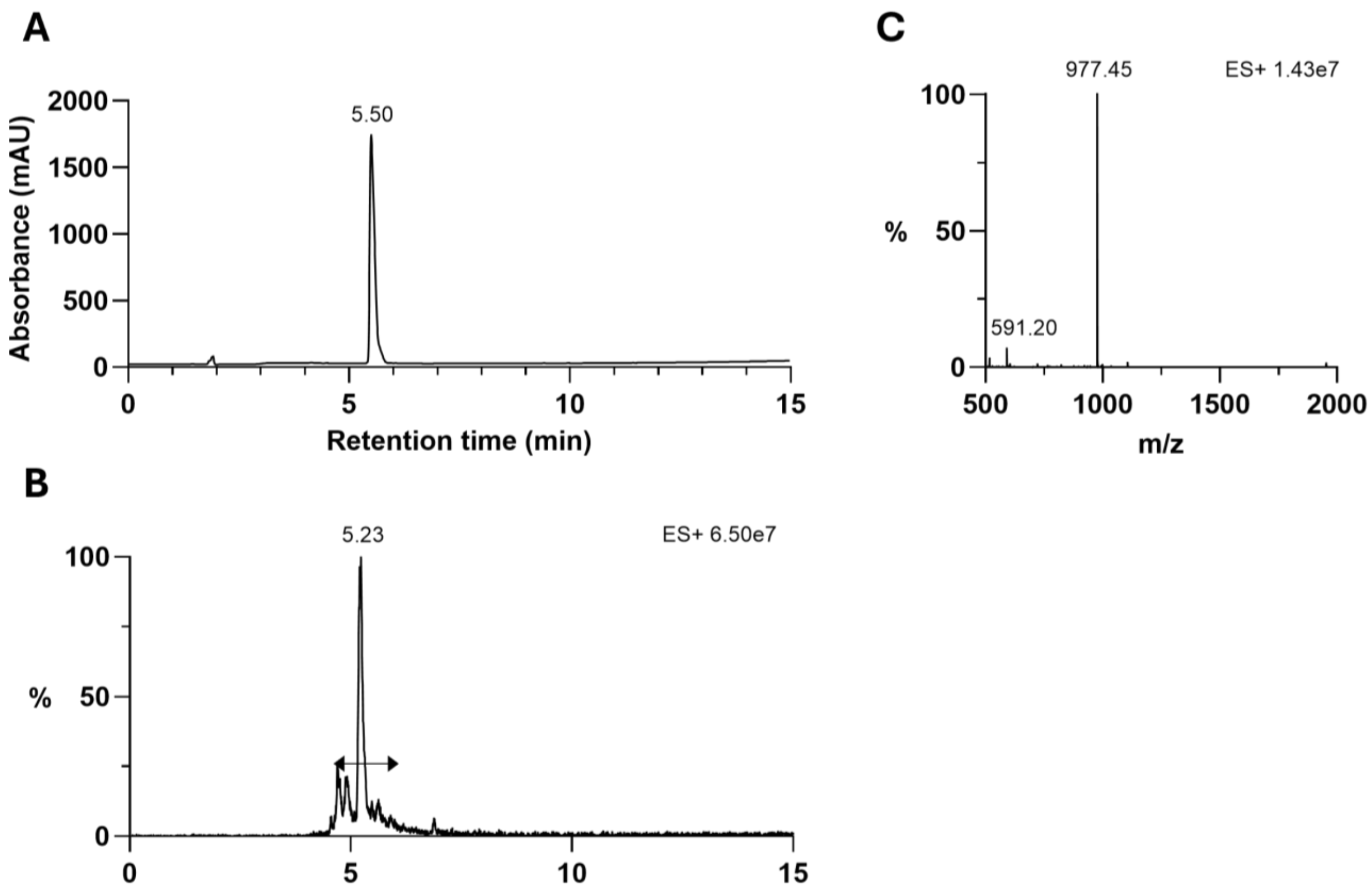

Figure S15. Characterisation of compound **S11**. **A.** Analytical UV-LCMS-HPLC chromatogram R_t = 5.50 min (5–95% B over 15 min, 220 nm). **B.** LRMS total ion count trace. **C.** LRMS ion abundance at R_t = 5.23 min.

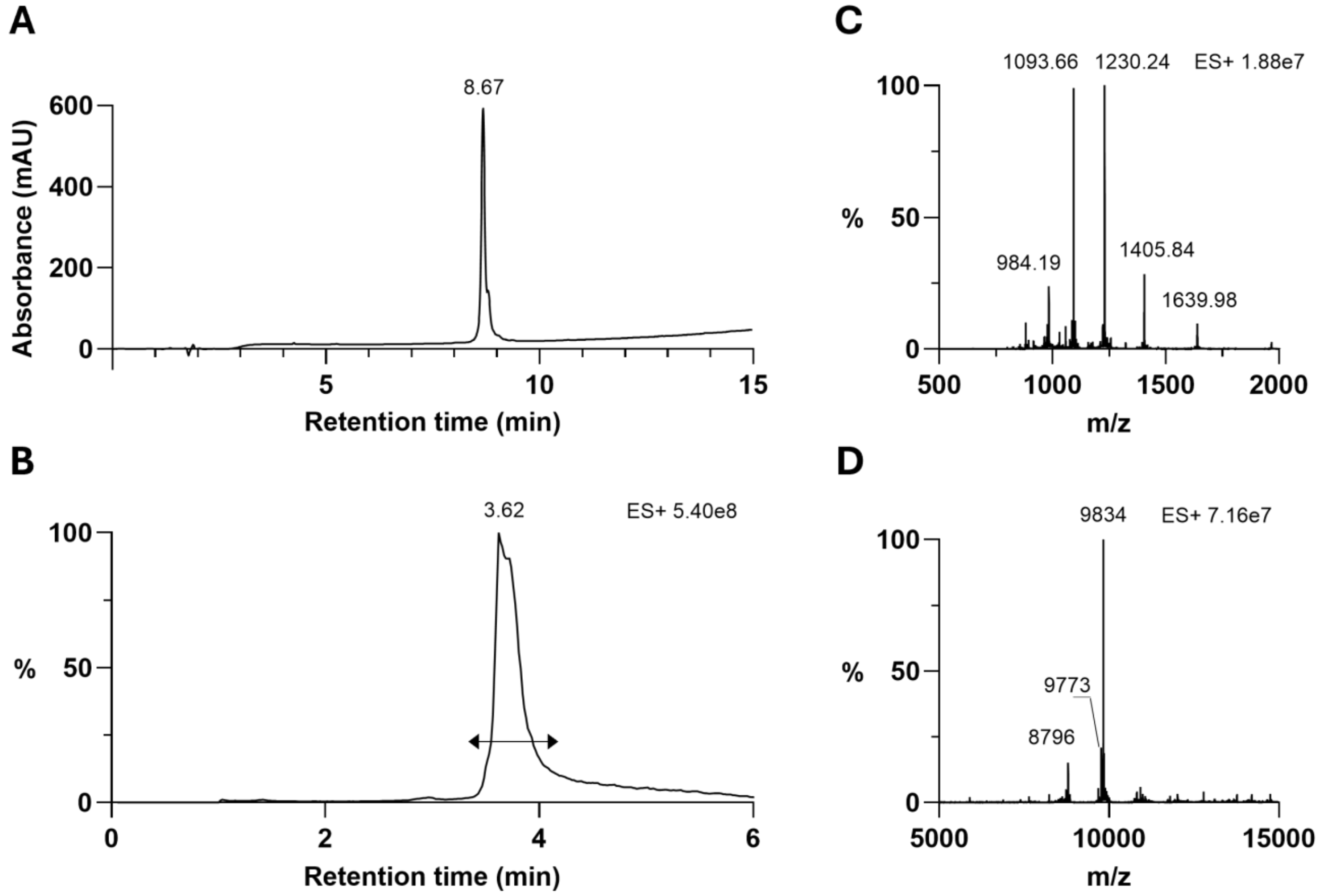

Figure S16. Characterisation of compound **13**. **A.** Analytical HPLC chromatogram R_t = 8.67 min (5–95% B over 15 min, 220 nm). **B.** LRMS total ion count trace (Peptide LCMS, Section 1.2). **C.** LRMS ion abundance at R_t = 3.62 min. **D.** LRMS deconvoluted trace.

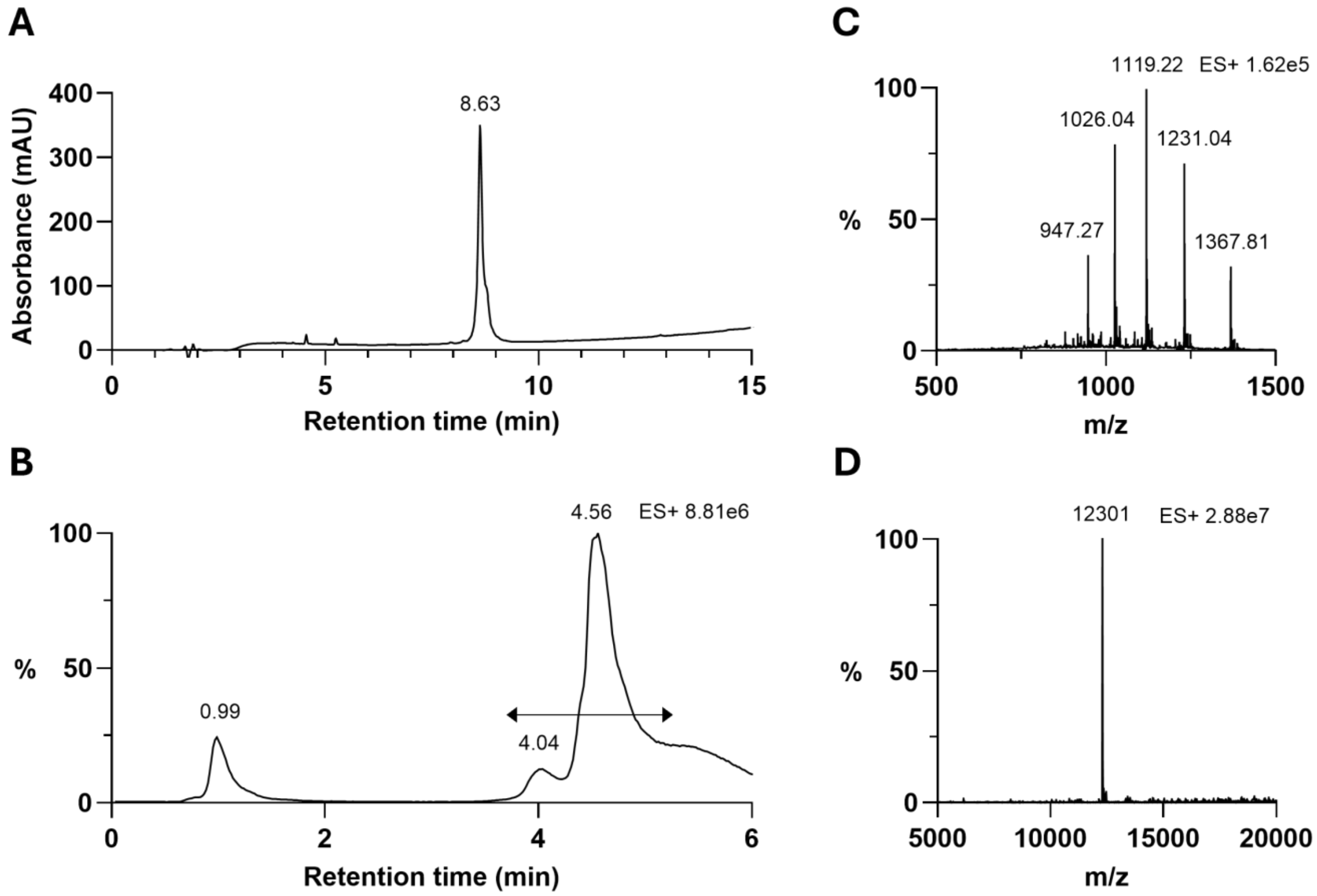

Figure S17. Characterisation of compound **14**. **A.** Analytical HPLC chromatogram R_t = 8.63 min (5–95% B over 15 min, 220 nm). **B.** LRMS total ion count trace (Peptide LCMS, Section 1.2). **C.** LRMS ion abundance at R_t = 4.56 min. **D.** LRMS deconvoluted trace.

2.1. Analytical UV-LCMS-HPLC of crude reaction mixtures

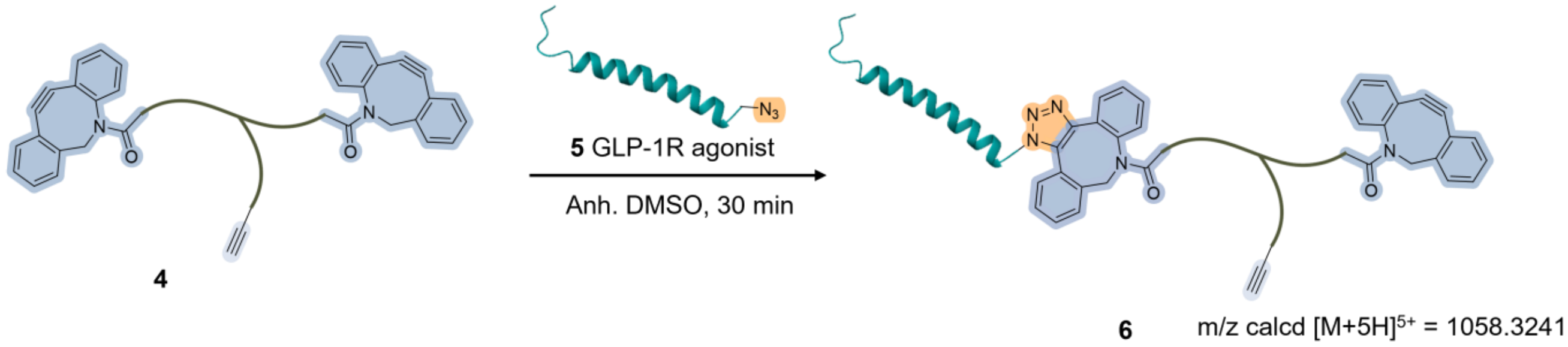

Reaction leading to the formation of compound **6** (as outlined in the Section 1.4.7.) was analysed before purification using analytical UV-LCMS-HPLC.

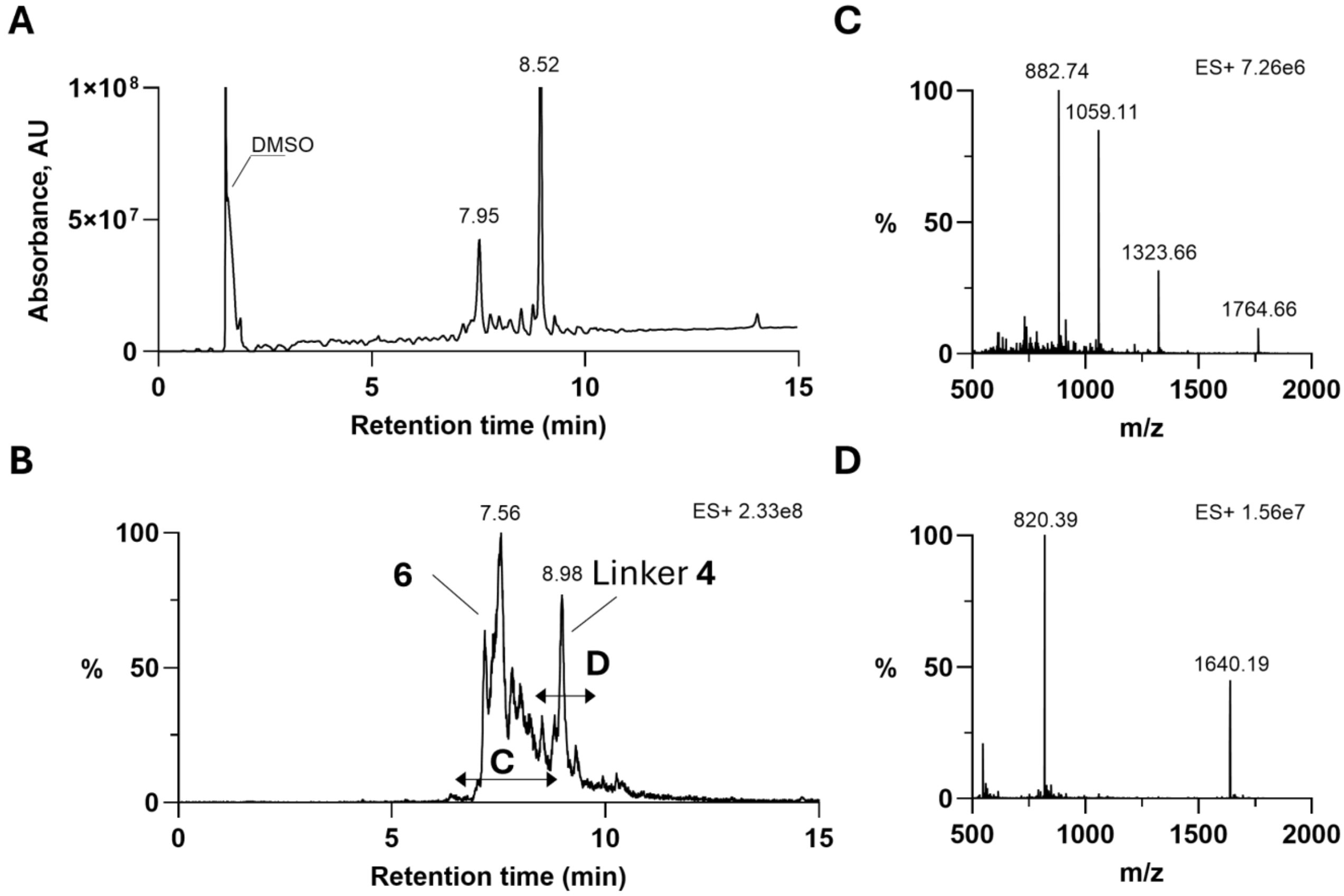

Figure S18. Analysis of crude **6**. **A.** Analytical UV-HPLC-LCMS chromatogram R_t (**6**) = 7.95 min, R_t (**4**) = 8.52 min (5–95% B over 15 min, 220 nm). **B.** LRMS total ion count trace. **C.** LRMS ion abundance at R_t = 7 – 8.8 min. **D.** LRMS ion abundance at R_t = 9 – 9.3 min

3. Compounds stability studies

Linker **4** (0.5 mg/ mL) in 1X PBS, pH 7.4, 0.5% DMSO was incubated either at room temperature (18 °C) or at 37 °C for 6 days. At times t = 0, 1, 2, 4, 8, 24, 48, 72, 96, and 120 h, aliquots were taken and flash frozen in liquid nitrogen. Similarly, Compound **8** (1 mg/mL) in 1X PBS, pH 7.4 was incubated at 37˚C for 24h. At times t = 0, 1, 2, 4, 8, and 24h aliquots were taken. Just before analysis, aliquots were thawed and 50 µL of each were mixed with equal volume of caffeine (625 µM) and analysed via analytical HPLC (5-95% B over 15 min, 254 nm). The experiments were performed in three independent repeats.

Further, compound **5**, compound **7**, and compound **8** (200 µM) were incubated in a solution of 25% human serum and 75% PBS at 37˚C. At time points t = 0, 1, 2, 4, 8, and 24h, aliquots were collected and immediately quenched with an equal volume of cold acetonitrile. Samples were placed on ice for 10 minutes, then centrifuged at maximum speed for 5 minutes. The resulting supernatant was collected for analysis. The experiment was performed in three independent repeats for compound **5** and **7**. Due to limited material availability, the experiment was performed in duplicate. To analyse the data, the ratio of integrated area under the peak for linker **4** vs caffeine at 254 nm was calculated for each time point. The stability percentage was calculated by normalising the linker vs caffeine integrated area under the peak ratio at time 0 h to 100%. Data from the three repeats was plotted as a mean ± SD. Curves were fitted by nonlinear regression using a four-parameter logistic model. No statistical comparisons were performed.

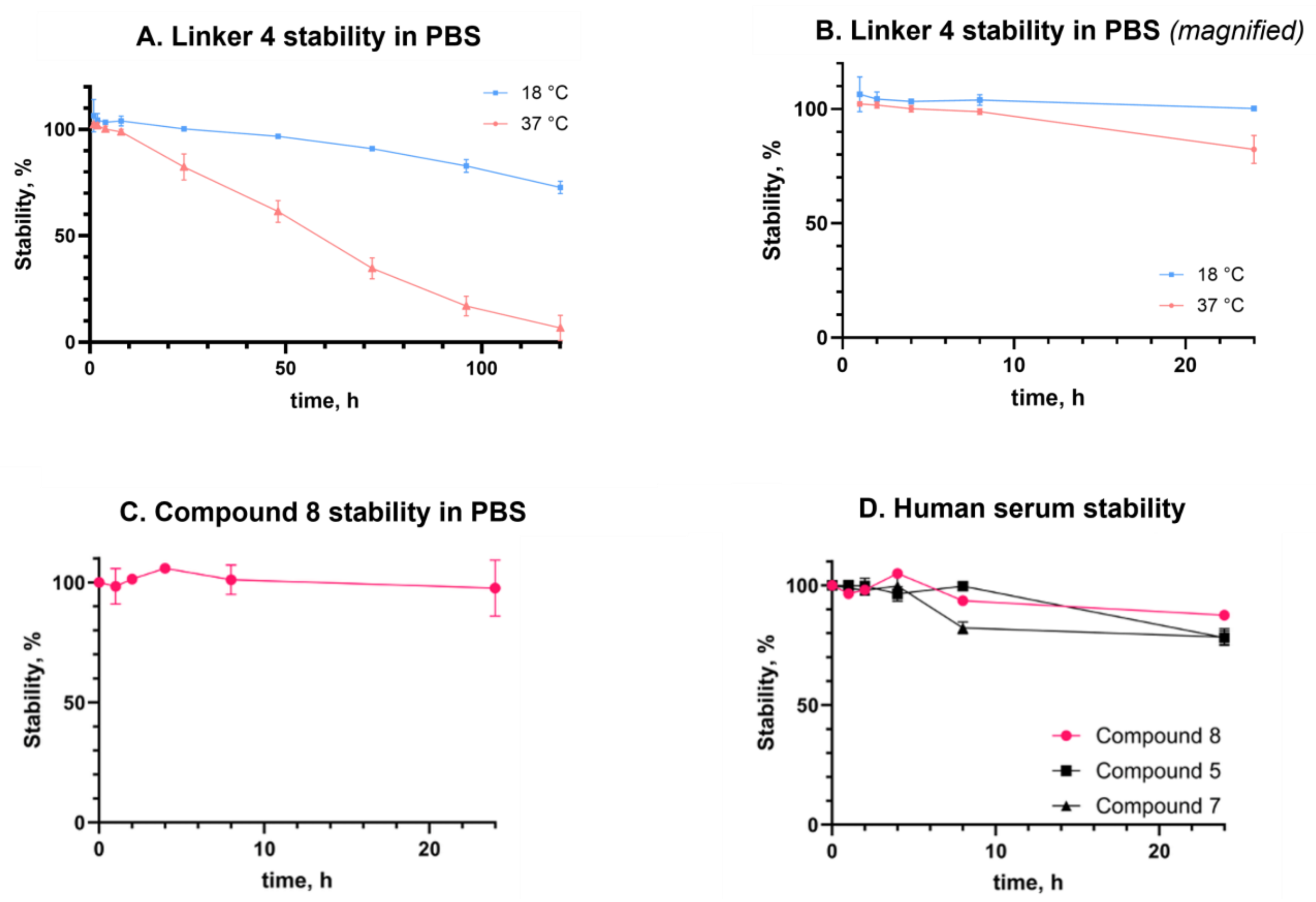

Figure S19. A. Linker **4** stability in PBS, pH 7.4 at 18 °C and 37 °C. Data from three repeats was plotted as a mean ± SD. Curves were fitted by nonlinear regression using a four-parameter logistic model. No statistical comparisons were performed. Some error bars may not visible due to being smaller than the

data point symbols on the graph. **B.** Linker **4** stability in PBS at pH 7.4, magnified between time 0 h and 24 h. C. Compound **8** stability in PBS. **D.** Human serum stability of compounds **5, 7** and **8.**

4. ICP-MS analysis

All samples were analysed on a Perkin Elmer Nexion 350D ICP-MS (Inductively Coupled Plasma – Mass Spectrometer), in the Department of Earth Sciences at the University of Cambridge, UK. The calibration standards were prepared as an external calibration using serial dilutions of a multielement standard SPS-SW2 (synthetic surface water solution, LGC Promochem, Sweden) in high purity 1% HNO_3 (~0.2M) (teflon distilled in house) consisting of calibration standards (blank, 100x, 10x, and 2x diluted from stock). The ICP-MS internal standards were 10 ppb Rh, In and Re and each sample was prepared in 1% HNO_3, added online with a t-piece and mixing tube prior to the nebuliser. An independently prepared quality control standard was used to verify the calibration was accurate (BCR-2, USGS, Colorado USA) and a secondary QC standard of a 10ppb multielement standard from QMX Labs (Thaxted, England) was analysed throughout the run to check for calibration accuracy (approx 5%-10%) with a similar precision. Instrumental drift was less than 10% measured for the raw intensity of the internal standards during the entire analytical run. Solutions were analysed using a Micromist FM05 microconcentric nebuliser using a pumped flow rate of 80 ul/min (Glass Expansion, Australia) and a quartz cyclonic baffled spray chamber with nickel sampler and skimmer cones. All elements were run in Helium KED mode with a He gas flow in the collision cell at a flow rate of 4 ml/min. ICP-MS sensitivity in this configuration was 1.5 x 105 cps/ppb In with CeO/Ce ratios less than 1%. Concentrations results were calculated using the Syngistix 3.5 software with a simple linear calibration line and intercept set to zero. The raw intensities were blank subtracted and internal standard normalised before calibration calculations were performed. All results (unknowns and standards) were accurately corrected for any dilutions by volume. Total copper content was reported as determined by the ^{65}Cu isotope, with results for ^{63}Cu matching within the method error of 5-10%. This indicates low concern about potential polyatomic ICP-MS interferences that might bias one of the isotopes too high in complex sample matrices.

Table S4. Copper content in final compounds as determined by ICP-MS

Cmpd.	^{63}Cu, µg/g	Total Cu, µg/g Determined by ^{65}Cu
**9**	10.3	10.1
**10**	15.5	14.8
**11**	10.7	10.9
**12**	11.2	11.3
**13**	1716.3	1738.9
**14**	2180.4	2219.6

5. Biological evaluation

- ***Cell Culture***

CHO-GLP1R cells were cultured at 37˚C, 95% humidity, 5%CO_2 in DMEM /F-12 (Gibco, #311320033) supplemented with 10% FBS (Gibco, #A5256701), 400 µg/mL Hygromycin B (Gibco, # 10687010), and 500ug/mL Geneticin (Gibco, #10131035). cAMP Hunter™ CHO-K1 CALCRL-RAMP3 Gs cells were cultured at 37˚C, 95% humidity, 5%CO_2 with AssayComplete™ Cell Culture Kit-107 (Eurofins, 92-3107G). Cells were cryopreserved at a density of 1 x 10^6 cells/mL in medium supplemented with 10% DMSO.

- ***cAMP accumulation***

Construct activity levels were assessed by quantifying intracellular cAMP levels following receptor activation. cAMP accumulation was measured with the HTRF cAMP Gs Dynamic Detection kit (Revvity, 62AM4PEB) in CHO cells stably overexpressing human GLP1R and in cAMP Hunter™ CHO-K1 CALCRL-RAMP3 Gs cells (Eurofins, #95-0169C2, RRID: CVCL_KU86). The assay relies on a Förster resonance energy transfer (FRET) signal generated by the binding of a monoclonal cAMP Europium Cryptate-labelled antibody (Europium donor) to a d2-labeled cAMP (red acceptor). Upon receptor activation, endogenous cAMP is generated and binds to the antibody, resulting in a loss of signal.

First, incubation buffer was prepared from HBSS (Gibco, #14025092) supplemented with 20 mM HEPES (pH 7.4) (Gibco, #15630080), 0.1% BSA (Sigma-Aldrich, #A9418), and 0.5 mM IBMX (Sigma-Aldrich, #410957). Next, compounds 1-6 and their respective controls were diluted in incubation buffer and dispensed onto a 384-well low-volume plate using a Labcyte ECHO (non-acoustic dispenser) for a final well volume of 5uL. Afterwards, CHO-GLP1R and cAMP Hunter™ CHO-K1 CALCRL-RAMP3 Gs cell vials were defrosted and their medium removed. 5uL per well of cell suspension containing either 1000 CHO-GLP1R cells or 100 cAMP Hunter™ cells were dispensed into the 384-well low-volume plate prepared earlier. For the CHO-GLP1R cell line, white 384-well low-volume plates (Greiner, #4513) were used, whilst black 384-well low-volume plates (Greiner, #4514) were used for the cAMP Hunter™ cells. The test compounds and cells were then incubated for 30 minutes at room temperature. The assay was performed according to the manufacturer's protocol with an incubation period of 60 minutes. CHO-GLP1R cells were provided by AstraZeneca and consisted of CHO cells stably expressing human GLP1R. Where applicable, the parental CHO-K1 cell line is listed in Cellosaurus as CHO-K1 (RRID:CVCL_0214). To our knowledge, the specific AstraZeneca-provided CHO-GLP1R derivative used in this study does not have a separate RRID.

A Pherastar Plus (BMG) plate reader was used to measure acceptor (665 nm) and donor (620 nm) emissions. To analyse the data, the ratio of 665 nm/620 nm * 10^4 was calculated for each well. Afterwards, the delta F (%) was calculated using the formula: Delta F (%) = (Ratio sample – Ratio Negative Control)/Ratio Negative Control × 100. The negative control wells, representing basal cAMP levels, contained cells, incubation buffer, monoclonal cAMP Europium Cryptate-labelled antibody, and

lysis buffer. The resulting data was analysed with a 4-Parameter Logistical Analysis in PRISM and the EC_{50} values for each sample were calculated.

- ***Microscopy***

Receptor internalisation was qualitatively assessed using fluorescent confocal microscopy to track the cellular localisation of compound **12**, which was fluorescently labelled with TAMRA. CHO-GLP1R cells were used as a receptor-expressing model system, while wild-type CHO cells served as a negative control to distinguish GLP-1R-dependent cellular uptake from non-specific cell-associated signal.

Wild-type CHO (RRID: CVCL_S544) and GLP1R-CHO cells were seeded at a density of 10,000 cells per well in a 96-well PhenoPlate (Revvity, #6055302). The cells were grown in a humidified incubator at 37°C with 5% CO_2. After 24 hours, the culture medium was replaced with phosphate-buffered saline (PBS; Gibco, #10010023) supplemented with 1% fetal bovine serum (FBS; Gibco, #A5256701). Afterwards, cells were either left untreated or treated with 60 nM of compound **12** for 5 minutes at 37°C.

Following treatment, all wells were washed three times with fresh culture medium. Cells were then fixed by adding 25 µL of 16% paraformaldehyde (Thermo Scientific, #043368.9M) to the existing 75 µL of medium in each well, resulting in a final concentration of 4% paraformaldehyde. The plates were incubated for 20 minutes at room temperature and then washed three times with PBS.

Cells were then stained with a solution containing 0.1% Triton X-100 (Sigma-Aldrich, #X100), Hoechst 33342 (1:10,000 dilution; Thermo Fisher Scientific, #62249) to label the nuclei, and CellMask Deep Red (1:200,000 dilution; Invitrogen, #C10046) to stain the entire cell. After a 15-minute incubation at room temperature, the cells were washed three times with PBS.

Images were acquired using either ImageXpress HT.ai (Molecular Devices) microscope in spinning-disc confocal mode with a X20 water immersion lens (N.A. 1.2) or with a Zeiss CellDiscovery 7 microscope in Airyscan multiplex mode with X25 water immersion lens (N.A. 1.2). Further processing and deconvolution of Airyscan mutliplex images was performed with Zen software (Zeiss). Image rendering was performed using a combination of Fiji and Zeiss Arivis software. The microscopy images were used as a qualitative comparison between receptor-negative and receptor-expressing cells.

5.1. cAMP accumulation results

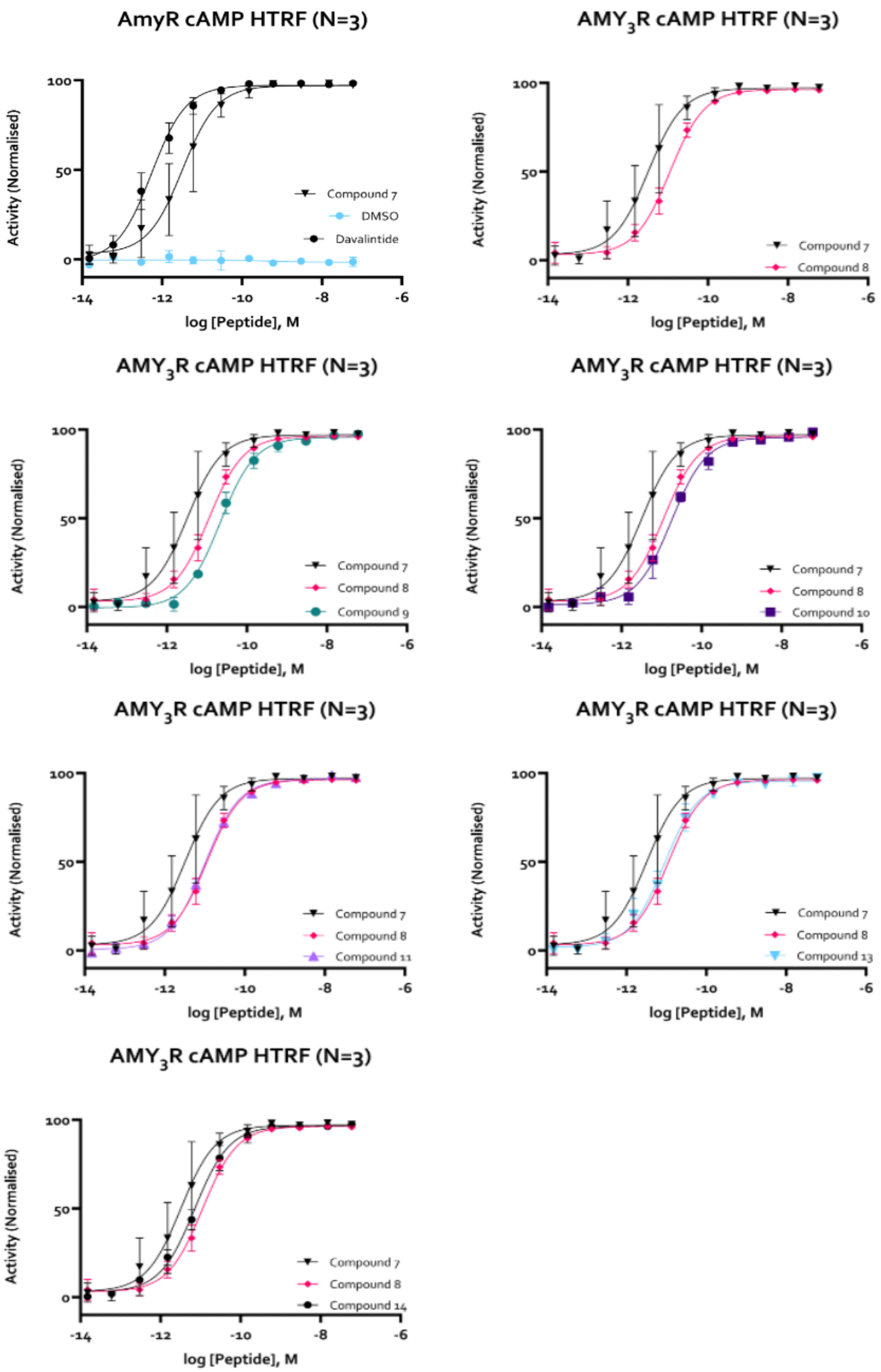

Figure S20. HTRF cAMP Gs Dynamic Detection assay comparing AmylinR agonist **7**, Compounds **8–11**, **13–14**, Davalintide (positive control), and DMSO (negative control) in cAMP HunterTM CHO-K1 CALCRL-RAMP3 Gs cells. Data are presented as mean ± SD from three independent experiments (n = 3), each performed in technical triplicate. Curves were fitted by nonlinear regression using a four-parameter logistic model. No statistical comparisons were performed.

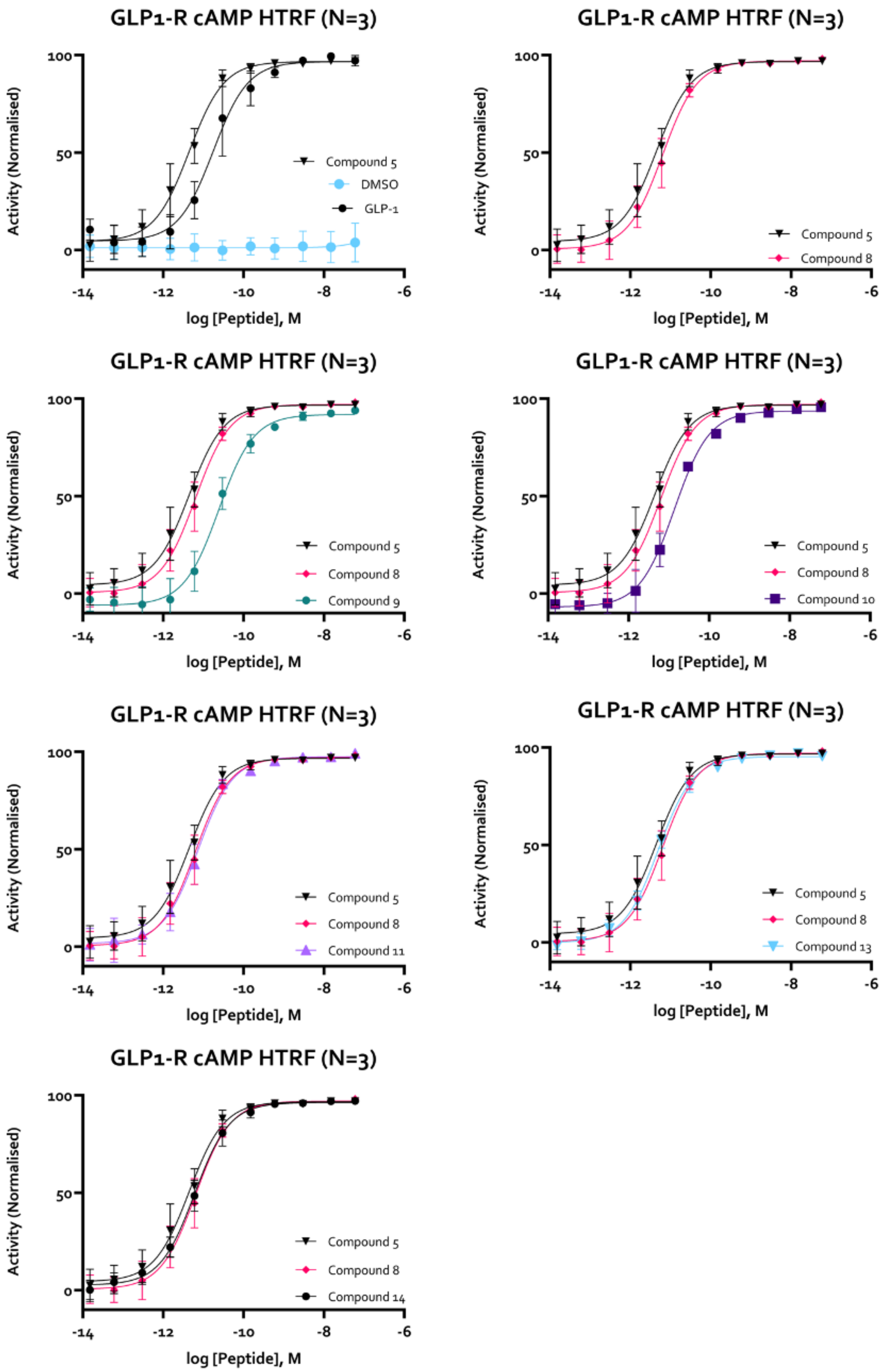

Figure S21. HTRF cAMP Gs Dynamic Detection assay comparing GLP1-R agonist **5**, Compounds **8–11**, **13–14**,, GLP-1 (positive control), and DMSO (negative control) in CHO-GLP1R cells. Data are presented as mean ± SD from three independent experiments (n = 3), each performed in technical triplicate. Curves were fitted by nonlinear regression using a four-parameter logistic model. No statistical comparisons were performed.

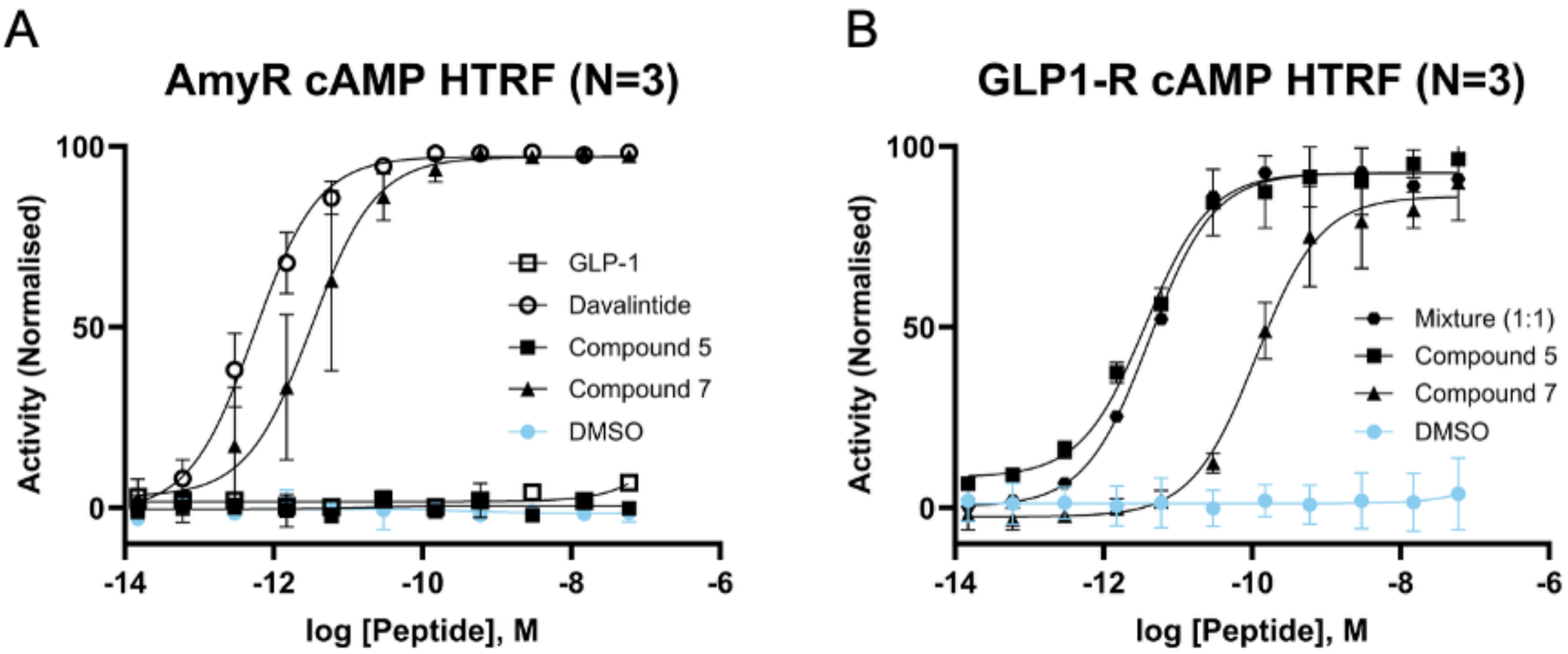

Figure S22. Selectivity controls for GLP-1R and Amy3R agonists. **A.** cAMP accumulation in CHO-Amy3R cells following treatment with Davalintide (positive control), compound **5** (GLP-1R agonist), compound 7 (AmylinR agonist), GLP-1 (negative control), and DMSO. Only compound **7** and Davalintide activated the Amy3R, confirming specificity; compound **5** and GLP-1 showed no meaningful activity. **B.** cAMP accumulation in CHO-GLP1R cells treated with compound **5**, compound **7**, and a 1:1 molar mixture of compounds **5** and **7**. Compound **7** showed weak activation at high concentrations, consistent with low-level AmylinR expression. However, the 1:1 mixture did not significantly enhance potency over compound **5** alone, confirming that GLP-1R drives the observed response in this assay. Data are presented as mean ± SD from three independent experiments (n = 3), each performed in technical triplicate. Curves were fitted by nonlinear regression using a four-parameter logistic model. No statistical comparisons were performed.

6. References

[1] G. Zhang, R. S. Annan, S. A. Carr, T. A. Neubert, "Overview of Peptide and Protein Analysis by Mass Spectrometry" *Current Protocols in Protein Science* **2010**, *62*, 16.1.1-16.1.30.

[2] S. Krajcovicova, T. Wharton, C. L. Driscoll, T. A. King, M. R. Howarth, D. R. Spring, "A platform for SpyCatcher conjugation to native antibodies" *Chem. Sci.* **2025**, *16*, 10602–10609.

[3] S. A. McNelles, J. L. Pantaleo, A. Adronov, "Highly Efficient Multigram Synthesis of Dibenzoazacyclooctyne (DBCO) without Chromatography" *Org. Process Res. Dev.* **2019**, *23*, 2740–2745.

[4] M. F. Debets, S. S. van Berkel, S. Schoffelen, F. P. J. T. Rutjes, J. C. M. van Hest, F. L. van Delft, "Aza-dibenzocyclooctynes for fast and efficient enzyme PEGylation via copper-free (3+2) cycloaddition" *Chem. Commun.* **2010**, *46*, 97–99.
